# A Flexible, Perfluorinated Analog of Aluminum Fumarate Metal–Organic Framework

**DOI:** 10.1002/chem.202500130

**Published:** 2025-05-29

**Authors:** Virginia Guiotto, Maria Sole Notari, Diletta Morelli Venturi, Alberto Ricchebuono, Melissa Castagnoli, Christoph Meier, Francesca Nardelli, Lucia Calucci, Matteo Signorile, Marco Taddei, Valentina Crocellà, Ferdinando Costantino

**Affiliations:** ^1^ Dipartimento di Chimica Centro di Riferimento NIS Unità di Ricerca INSTM Università degli Studi di Torino Via G. Quarello 15/A and Via P. Giuria 7 I‐10125 Torino Italy; ^2^ Dipartimento di Chimica, Biologia e Biotecnologie Unità di Ricerca INSTM Università di Perugia Via Elce di Sotto 8 06123 Perugia Italy; ^3^ Institute of Inorganic Chemistry Christian‐Albrechts University of Kiel Max‐Eyth Straße 2 Kiel Germany; ^4^ Kiel Nano, Surface and Interface Science KiNSIS Christian‐Albrecht University of Kiel Christian‐Albrechts‐Platz 4 24118 Kiel Germany; ^5^ Istituto di Chimica dei Composti Organo Metallici Unità di Ricerca INSTM Consiglio Nazionale delle Ricerche Via Giuseppe Moruzzi 1 56124 Pisa Italy; ^6^ Centro per l'Integrazione della Strumentazione Scientifica dell'Università di Pisa (CISUP) Lungarno Pacinotti 43/44 56126 Pisa Italy; ^7^ Dipartimento di Chimica e Chimica Industriale Unità di Ricerca INSTM Università di Pisa Via Giuseppe Moruzzi 13 56124 Pisa Italy

**Keywords:** advanced characterization, CO_2_ adsorption, flexibility, perfluorinated MOFs, S‐shaped isotherms

## Abstract

Herein, we report the synthesis of Al‐TFS, a novel aluminum metal–organic framework (MOF) based on tetrafluorosuccinic acid (H_2_TFS), of formula Al(OH)(TFS)·1.5H_2_O, introducing a new member to the family of perfluorinated MOFs. The structure of the MOF, solved *ab‐initio* from laboratory powder X‐ray diffraction data, displays analogies with that of the commercially available Al‐fumarate (Basolite A520). The structure is composed of 1D infinite OH‐bridged AlO_6_ octahedra chains connected by the dicarboxylic linkers, designing rhombic channels decorated by fluorine atoms. Upon water removal, the MOF undergoes a phase transition leading to a moderate expansion of the unit cell. Volumetric analysis revealed the presence of *ultra‐*micropores with a size lower than 4 Å. Gas sorption measurements demonstrated for Al‐TFS a slightly higher CO_2_ selectivity compared to N_2_ and CH_4_ than the Al‐fumarate analog, with peculiar shapes of the isotherms suggesting a dynamic response of the framework to CO_2_ adsorption. Using different complementary techniques (in situ infrared spectroscopy, powder X‐ray diffraction, solid‐state nuclear magnetic resonance spectroscopy, gas/vapor sorption), and density functional theory simulations, the flexibility of the new MOF was disclosed, highlighting the significant impact of fluorination in tailoring materials with structural flexibility and peculiar adsorption properties.

## Graphical abstract

A novel flexible perfluorinated Al‐MOF is discovered, highlighting the impact of linker fluorination on structural flexibility and sorption properties.

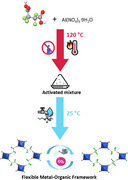

## 1 Introduction

The chemistry of metal–organic frameworks (MOFs) is nowadays strongly oriented toward practical applications for industry,^[^ ^1^, ^2^, ^3^ ^]^ climate change remediation,^[^ ^4^, ^5^, ^6^ ^]^ catalysis,^[^ ^7^, ^8^ ^]^ and biomedical purposes.^[^ ^9^, ^10^ ^]^ In this context, MOFs based on simple, commercially available linkers and earth‐abundant metals are particularly investigated by the scientific community.^[^ ^11^, ^12^, ^13^ ^]^ Aluminum is one of the most attractive metals, as Al^III^ is a very hard cation that can form strong bonds with carboxylic linkers, affording stable MOFs that have been intensively studied and have occasionally reached a moderate success at the industrial level. The most representative examples of Al‐based MOFs originate from the Institute Lavoisier at Versailles (MIL), where famous Al‐based MOFs such as MIL‐53,^[^ ^14^ ^]^ MIL‐96,^[^ ^15^ ^]^ MIL‐100,^[^ ^15^ ^]^ and MIL‐110^[^ ^16^ ^]^ were designed for the first time. These MOFs exhibit distinctive features that have made them the focus of numerous studies. For example, MIL‐53(Al), commercially marketed by the chemical company BASF under the name of Basolite A100, has attracted considerable attention thanks to its unique breathing behavior, allowing the framework to expand or contract in response to external stimuli. The adsorption‐induced flexible mechanism enables its use in several applications concerning gas separation.^[^ ^17^, ^18^, ^19^ ^]^ For instance, Basolite A100 revealed interesting capacities of selective adsorption of hydrogen sulfide, which can be exploited in the purification of natural gas.^[^ ^20^ ^]^ On the other hand, more recent studies have discussed the potential use of MIL‐100 and MIL‐101 for water adsorption applications, given the high hydrolytic stability of Al‐based MOFs.^[^ ^21^, ^22^ ^]^ In addition, thanks to the high surface areas, water is not the only investigated adsorbate, since MIL‐100(Al) has also been tested for CO_2_ capture.^[^ ^23^ ^]^

BASF has also developed another well‐known Al‐based MOF, Al‐fumarate (Al‐FUM), which has been one of the first commercial MOFs under the trade name of Basolite A520.^[^ ^24^, ^25^ ^]^

Al‐FUM, of formula Al(OH)(FUM)·4H_2_O (FUM^2−^ is the fumarate anion) has the same topology of the well‐known Al‐terephthalate MIL‐53 MOF, but it is characterized by a rigid structure (absence of flexibility) and smaller pores.^[^ ^26^ ^]^ Compared to the preliminary synthetic route of Al‐FUM that required the use of *N*,*N*‐dimethylformamide (DMF) as solvent, the synthesis has been optimized by BASF, developing an organic solvent‐free preparation, where DMF has been substituted with water. The sustainable synthesis of Al‐FUM has been a key point to move the production from a small laboratory to an industrial scale.^[^ ^27^ ^]^ Thanks to the good thermal and water stability, high surface area, and low cost of the metal precursor, Basolite A520 has found applications in many fields, including sorption and separation,^[^ ^28^, ^29^, ^30^ ^]^ catalysis,^[^ ^24^ ^]^ desalination,^[^ ^31^ ^]^ removal of phosphates and fluorides from water,^[^ ^32^ ^]^ and as porous filler in mixed matrix membranes.^[^ ^33^ ^]^ In terms of commercial applications, Al‐FUM has been employed to store and deliver natural gas for the automotive industry since it revealed better capacities for storing CH_4_ compared to Basolite C200 (HKUST‐1).^[^ ^27^ ^]^ Furthermore, Al‐FUM is also known for its hydrophilic character and for the excellent hydrothermal stability, and it has been selected as a promising adsorbent for heat transformation applications.^[^ ^34^, ^35^ ^]^ On the other hand, the hydrophilic character of Al‐FUM limits its use as a sorbent for CO_2_ capture from wet streams, since the presence of water has a significant impact on its CO_2_ adsorption capacity.^[^ ^30^ ^]^

A promising field of research in MOF chemistry deals with the functionalization of the framework as a powerful strategy to tune physico‐chemical properties. The synthesis of fluorinated MOFs (F‐MOFs), where fluorine can be present either as a substituent on the organic linker or in the inorganic units, such as metal or semi‐metal fluoride anions, is becoming increasingly relevant.^[^ ^36^, ^37^ ^]^ For instance, the steric hindrance of fluorine atoms can impact the binding angle between metal nodes and organic linker, influencing the pore dimensions of the framework.^[^ ^36^ ^]^ Moreover, due to the high polarity of C─F or metal‐F bonds, F‐MOFs often display good affinity toward species with Lewis acidic character, such as CO_2_, enhancing the adsorption selectivity over less interactive molecules, like N_2_ and CH_4_, which makes them good solid sorbents for gas separations.^[^ ^36^ ^]^ Additionally, decoration of the pore walls with fluorine atoms has been found to significantly modify water adsorption. In some cases, fluorine atoms play a crucial role in reducing the affinity with H_2_O,^[^ ^37^, ^38^ ^]^ while in other cases fluorine atoms become new, additional adsorption sites for this molecule.^[^ ^39^ ^]^ In general, F‐MOFs are gaining more and more interest in the field of gas separations due to their tunable textural properties, enhanced selectivity and enhanced stability.^[^ ^40^ ^]^

We recently reported several perfluorinated analogs, using both aromatic and aliphatic linkers, of benchmark MOFs, such as UiO‐66(Ce),^[^ ^41^ ^]^ MIL‐140A(Ce),^[^ ^41^ ^]^ MOF‐801(Zr),^[^ ^42^ ^]^ and MIL‐53(Al).^[^ ^43^ ^]^ The latter, named F_4_‐MIL‐53(Al), was obtained by employing a solvent‐free synthetic route. The MOF structure is strongly related to that of the well‐known MIL‐53(Al), but its flexible behavior appears to be heavily influenced by the fluorination of the terephthalic linker, and it shows a phase transition purely induced by temperature with an extremely narrow hysteresis.^[^ ^43^ ^]^ On the other hand, F_4_‐MIL‐140A(Ce) features a peculiar cooperative CO_2_ adsorption mechanism that gives rise to a non‐hysteretic step‐shaped isotherm. The role of the perfluorinated linker in this mechanism is crucial, as it involves the concerted rotation of the linker's aromatic rings.

Following the same strategies described for the above‐mentioned MOFs, we sought to prepare a per‐fluorinated analog of Al‐FUM and to explore the impact of fluorine atoms in the linker on the adsorption properties. Fluorination of fumaric acid is not a straightforward synthetic route, as it involves the use of hazardous reagents (gaseous F_2_ and HF), and the product cannot be easily supplied by most chemical companies.^[^ ^44^ ^]^ We therefore decided to employ the cheap and commercially available tetrafluorosuccinic acid (H_2_TFS) as a linker. We recently found that the steric hindrance of the four fluorine atoms in the aliphatic chain makes this linker amenable to adopt a pseudo‐*trans* conformation and behave as a topological analog of fumarate,^[^ ^42^ ^]^ making it promising for obtaining a MOF with the same topology and permanent porosity of Al‐FUM. Actually, the utilization of succinic acid has already been investigated in the synthesis of Al‐succinate (Al‐SUC).^[^ ^45^, ^46^ ^]^ Based on the similar PXRD patterns, Al‐SUC is presumed to display the same structure topology of Al‐FUM. However, it exhibits different properties, including enhanced hydrophobicity, resulting from the different position of the protruding H atoms on the carbon chain.^[^ ^46^ ^]^

Here, we report on the synthesis of the perfluorinated Al‐FUM topological analog, Al‐TFS, and on the detailed characterization of its crystal structure, adsorption properties, thermal stability, and water affinity. The flexible behavior of the new MOF, triggered by both water and carbon dioxide adsorption, was discovered. The structural features and sorption properties of the novel Al‐TFS were compared to those of the commercial Al‐FUM, disclosing the benefits and limitations of introducing fluorine atoms in the MOF structure.

## 2 Results and Discussion

### 2.1 Synthesis and Basic Characterization

**Figure 1:**
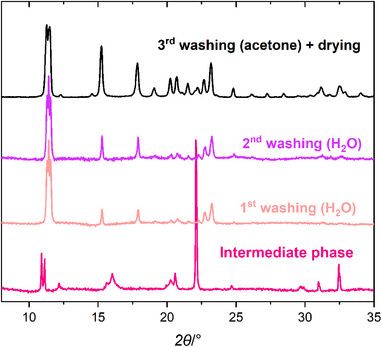
PXRD patterns of the “intermediate phase” after 24 h in oven at 393 K and before the workup (magenta) and of Al‐TFS after the first (light pink) and the second (purple) washing in water for 15 min and after the third washing in acetone (black). The diffraction patterns were recorded on wet materials, except for the black one.

Al‐TFS was synthesized following a “shake and bake” synthetic route, already demonstrated to be effective for the synthesis of Al‐ and Zr‐based MOFs in previous reports.^[^ ^30^, ^43^ ^]^ Briefly, the synthesis was carried out by mixing the solid reagents, namely the Al^III^ precursor [Al(NO_3_)_3_·9H_2_O] and the linker [H_2_TFS], in a mortar in equimolar quantities and heating in a PTFE vessel at 393 K overnight. The formation of the solid was monitored by collecting PXRD patterns at different times before and after the heating/washing treatment. The PXRD patterns obtained on the physical mixture after 2 and 10 min (Figure S1, Supporting Information, orange and light brown patterns) were compared with those of the pure reagents (Figure S2, Supporting Information, bright green and dark yellow curves). The patterns of both mixtures clearly show that no reaction occurred, as they simply display the reflections of unreacted Al(NO_3_)_3_·9H_2_O and H_2_TFS. After 24 h in the oven at 393 K, the “intermediate phase” was formed, and the reagents were completely consumed. Indeed, the PXRD pattern (Figure 1; Figures S1 and S2, Supporting Information, magenta pattern) shows peaks ascribable to the MOF under formation and other peaks for which a search and match analysis in the International Centre for Diffraction Data database gave no acceptable results. It is possible that the observed reflections come from a precursor phase of the MOF, containing both aluminum and the linker, which transforms into the MOF structure after washing. Indeed, Figure 1 shows that pure Al‐TFS forms during the first washing step in water (light pink pattern) and that crystallinity improves after the successive washing steps with water and acetone (purple and black patterns). The final crystalline Al‐TFS was obtained after drying in air at 333 K (Figure 1, black pattern; Figure S3, Supporting Information). Hereafter, this material will be referred to as “as‐synthesized Al‐TFS.”

FE‐SEM images of the final Al‐TFS powder revealed irregular aggregates made of ill‐defined particles (Figure S4, Supporting Information). The TGA curve of as‐synthesized Al‐TFS, reported in Figure S5 (Supporting Information), exhibits a weight loss of 10.1% below 373 K, attributed to the desorption of water and suggesting the formula Al(OH)(TFS)·1.5H_2_O (calculated loss of 10.4%). The weight loss of 78.6% observed from 553 K to 1273 K is due to the decomposition of the framework, which occurs in two distinct steps. The first step, accounting for 57.5%, is associated with the combustion of the organics, with the formation of Al_2_(OH)_3_F_3_ (calculated loss of 58.3%), identified by PXRD analysis of the residue of calcination at 873 K (Figure S6, Supporting Information). In the second step, a further 19.6% weight loss takes place, which can be attributed to the decomposition of Al_2_(OH)_3_F_3_ to volatile AlF_3_ and Al_2_O_3_. The analysis of the TGA residue revealed only the presence of α‐Al_2_O_3_ (Figure S7, Supporting Information), confirming our interpretation. At the end of the analysis, the amount of Al in α‐Al_2_O_3_ was half the original one in the MOF, while the other half had sublimed as AlF_3_. The calculated weight loss accounting for such Al loss during the last step is 21.4%, in good agreement with the experimental one.

ICP‐OES and quantitative ^19^F solution NMR analysis (see Figure S8, Supporting Information, and subsequent text) provided results in good agreement with the proposed formula for Al and TFS^2−^ wt%, respectively. An experimental value of 8.09% aluminum (calc. 10.42%) was obtained by ICP‐OES analysis.

### 2.2 Crystal Structure

**Figure 2:**
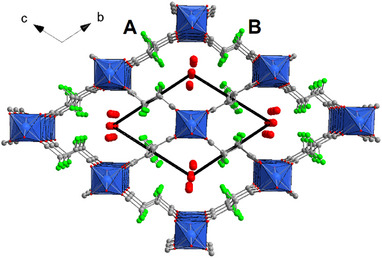
Crystal structure of as‐synthesized Al‐TFS viewed along the *a*‐axis. Color code: Al, blue; C, gray; O, red; F, green.

The crystal structure of as‐synthesized Al‐TFS (Figure 2) was solved ab initio from PXRD data using direct space Monte Carlo methods and the simulated annealing algorithm implemented in the program *FOX*.^[^ ^47^ ^]^ The structure was then refined with the Rietveld method using the program *TOPAS*.^[^ ^48^ ^]^ Structure determination and refinement details, and Rietveld plot are reported in Table S1 and Figure S9 (Supporting Information), respectively. As‐synthesized Al‐TFS crystallizes in the triclinic system in the *P*‐1 space group with cell parameters *a* = 6.631 (2) Å, *b* = 8.69(2) Å, *c* = 8.916(2) Å, *α* = 107.545(2)°, *β* = 109.243(2)°, and *γ* = 103.979(2)° (Figure S10, Supporting Information). The unit cell volume (436.78(2) Å^3^) is about half that of Al‐FUM (990 Å^3^).^[^ ^26^ ^]^ However, since Al‐FUM crystallizes in the monoclinic *P*2_1_/*c* space group, the volume per Al(OH)(L) (where L is the dicarboxylic linker) formula unit is comparable: 218.4 Å^3^ for Al‐TFS and 247.5 Å^3^ for Al‐FUM. Due to the smaller unit cell and the heavier and bulkier linker, the crystallographic density of as‐synthesized Al‐TFS is 1.97 g·cm^−3^, compared to 1.54 g·cm^−3^ of Al‐FUM.

The asymmetric unit of as‐synthesized Al‐TFS contains two crystallographically independent Al atoms sitting on inversion centers, one O atom bridging between two Al atoms (the H atom of the hydroxide cannot be located from PXRD data) and two crystallographically independent half TFS^2−^ fragments (marked with A and B, respectively, in Figure 2), which generate a full fragment each by inversion symmetry, and two O atoms representing adsorbed water molecules.

The crystal structure is composed of infinite 1D chains built from the connection of AlO_4_(OH)_2_ octahedra and running along the *a*‐axis, connected by the TFS^2−^ linkers in the remaining two dimensions. Notably, the *a*‐axis measures 6.631 Å, similarly to the *a*‐axis of Al‐FUM (6.842 Å).^[^ ^26^ ^]^ The connection of the Al‐based 1D units via the TFS^2−^ linkers designs small rhombic channels decorated by F atoms, with the shortest F‐F distance across the channel diagonal of 6.519 Å (measured from the atomic centers) and Al‐Al distances across the channel of 9.3662 and 14.348 Å (Figure S11, Supporting Information). In Al‐FUM, the shortest C‐C distance across the channel diagonal is 9.45 Å and the Al‐Al distances are 12.003 and 12.088 Å (Figure S12, Supporting Information), i.e., the channels are essentially square.

1.5 water molecules per formula unit are located in the channels of as‐synthesized Al‐TFS, with one water molecule (Ow2) occupying a special position corresponding to an inversion center. These water molecules are involved in a network of hydrogen bonds, both between themselves and with the ‐OH groups in the inorganic unit, with O‐O distances ranging between 2.594 and 2.789 Å (Figure S13, Supporting Information). These distances are considerably shorter than those between each water molecule and the F atoms of the linkers, which range from 3.341 to 3.854 Å (Figures S15 and S16, Supporting Information). This structure was additionally optimized by DFT (removing the water associated to the Ow2 atom), showing as the water molecule Ow3 directly interacts with the framework by accepting an H‐bond from the ─OH groups in the inorganic chain (Ow3‐HO distance of 1.717 Å) and donates weaker H‐bonds to F atoms of the linker (Hw‐F distances of 2.331, 2.411, and 2.627 Å), as depicted in Figure S14 (Supporting Information). In comparison, Al‐FUM contains four water molecules per formula unit, due to the larger void space available in its framework.

To monitor the thermal behavior of Al‐TFS, variable temperature PXRD (VT‐PXRD) was performed in the 313–713 K temperature range, collecting patterns every 40 K (Figure S17, Supporting Information). The MOF has undergone a complete phase transition already at 353 K, upon removal of water molecules from the channels, persisting in the new phase until 633 K. Above 633 K, the pattern suffers from peak broadening, and a generalized shift toward higher 2*θ* angles occurs, suggesting that the framework is starting to collapse. These structural changes are irreversible when the temperature is brought back to 303 K. To perform structural analysis of the phase formed above 353 K, Al‐TFS was heated at 393 K overnight inside a capillary that was subsequently sealed and analyzed by PXRD (Figure S18, Supporting Information). The PXRD pattern of the new phase, named “evacuated Al‐TFS”, was indexed with a monoclinic unit cell with the following lattice parameters: *a* = 13.2864(6) Å, *b* = 6.6385(3) Å, *c* = 10.4557(5) Å, *β* = 91.028(2)°, volume = 922.06(6) Å^3^. The analysis of systematic extinctions suggested *P*2_1_/*n* as the most probable space group, which was confirmed by Pawley refinement (Figure S19, Supporting Information). The volume per formula unit is 232.02 Å^3^, corresponding to a 6.4% expansion compared to the as‐synthesized Al‐TFS. Attempts to solve the structure of this phase provided a model consistent with the topology of the as‐synthesized phase and suggesting a conformational change in linker A (Figure S20, Supporting Information). However, Rietveld refinement was not successful, mainly because of the lack of calculated intensity for the −110 reflection (located at 14.87 °2θ), which caused an unacceptably large Rwp value (Figure S20, Supporting Information). This effect could be associated with disorder in the aliphatic chain of the linkers, which is challenging to model based on PXRD data. The structural model found with *FOX* was used as a starting model for DFT optimization, which was performed in the same unit cell, but all symmetry elements were removed (see experimental section for details). DFT optimization converged toward a final structural model consistent with the starting one obtained from *FOX*.

### 2.3 In Situ IR Spectroscopy

In situ IR measurements were collected to disclose the optimal activation procedure of Al‐TFS, performed to clean the surface from possible traces of water or acetone (solvents used during the washing step) prior to adsorption experiments and to further investigate a possible phase transition upon evacuation. The IR spectrum of as‐synthesized Al‐TFS (Figure S21, Supporting Information) is characterized by very intense and broad signals in the 3600–2600 cm^−1^ range, suggesting that the micropores are full of physisorbed water molecules. To evacuate the sample, the IR cell was connected to a vacuum line, and spectra were automatically collected every 180 s, during outgassing at room temperature. Except for the first spectrum in the outgassing sequence, which resembles the initial IR profile (black spectrum), the spectra of Al‐TFS in the high‐frequency region undergo significant changes. The intense and broad signals due to the presence of water quickly disappear, while a narrow component at ≈3700 cm^−1^, ascribed to the stretching vibration of the structural OH group bridging two Al atoms,^[^ ^26^ ^]^ increases. Al‐TFS was evacuated overnight at 353 K to totally remove any trace of water. The spectrum of the activated sample (red curve) is mainly characterized by the narrow ν(μ_2_‐OH) stretching band centered at 3683 cm^−1^ and by the out‐of‐scale signals of the framework vibrational modes in the low frequency region (1900–600 cm^−1^).^[^ ^26^ ^]^ According to these results, all the experiments carried out on evacuated Al‐TFS, and described in the following sections, were preceded by an overnight activation procedure at 353 K under dynamic vacuum.

**Figure 3:**
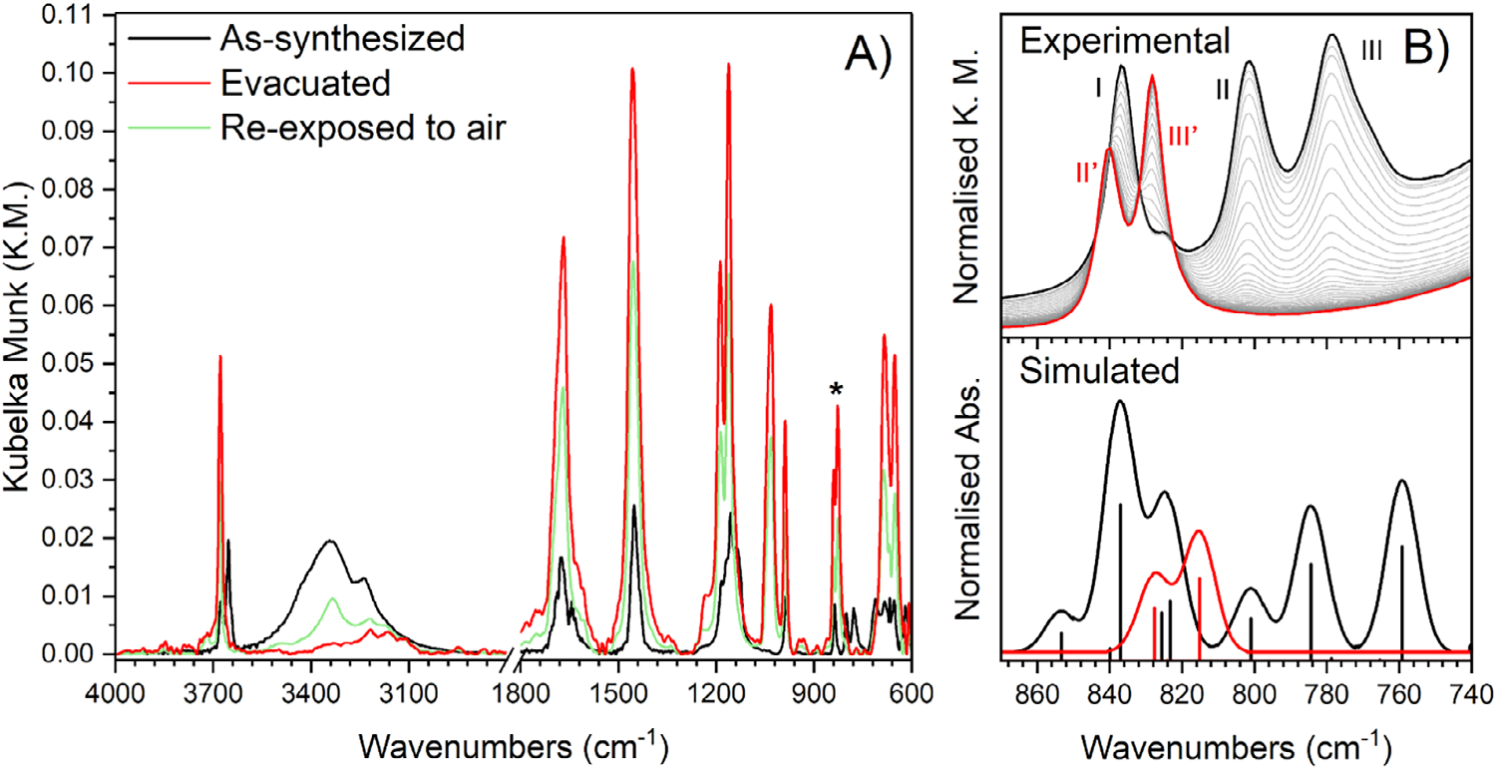
A) DRIFT spectra in the mid‐IR region of as‐synthesized Al‐TFS collected at room temperature (black curve), of evacuated Al‐TFS collected at 353 K (red curve), and of Al‐TFS exposed to air for 3 h at room temperature after the activation procedure (light green curve). The asterisk identifies the main spectral modification that has been attributed to the occurrence of a phase transition after DFT calculations. B) Comparison of the experimental time‐resolved DRIFT spectra with the simulated IR spectra in the range of the phase transition‐sensitive vibrational modes (870–740 cm^−1^). The initial and final states are represented in black and red, respectively. Intermediate states are represented in light grey.

Examples reported in the literature identify IR spectroscopy as a powerful technique to study phase transitions induced by water removal, through the observation of perturbations of specific vibrational modes.^[^ ^49^ ^]^ Unfortunately, the framework vibrations of MOFs fall in the low frequency region of the mid‐IR spectrum, and they are usually out‐of‐range when spectra are collected in transmission. To overcome this issue, in situ experiments were carried out by using a diffuse reflectance IR (DRIFT) apparatus equipped with an environmental chamber to work in a controlled atmosphere. The DRIFT spectra of as‐synthesized and evacuated Al‐TFS, collected by diluting the sample with Si powder, are shown in Figure 3A. As expected, the spectrum of evacuated Al‐TFS (red curve) differs significantly from that of the as‐synthesized MOF (black curve).

To shed light on the nature of the phase transition and its dynamics, a time‐resolved DRIFT experiment was performed, and the IR spectra of the initial and final states were simulated by static DFT calculations. The comparison of the experimental and simulated IR spectra in the 1800–600 cm^−1^ range, along with their discussion, is provided in Section S3 of Supporting Information (see Figure S22, Supporting Information). After a detailed analysis of the computed vibrational modes, two main diagnostic bands were identified for the phase transition in the 870–740 cm^−1^ region. A comparison of the experimental and simulated spectra in the above‐mentioned spectral range is reported in Figure 3B. Bands II and III, located at 759 and 784 cm^−1^ in the experimental spectrum of as‐synthesized Al‐TFS (black curve), are assigned to two wagging modes of the carboxylate groups, sharply decoupled by any vibration of the water molecules network. Band I (at 839 cm^−1^) arises from an overlap of contributions of different vibrational modes dominated by the presence of water in interaction with the ─OH group and the alkyl chains of Al‐TFS. Upon water desorption, band I vanishes in the experimental spectrum (red curve) and only two contributions are still present in the same region (band II’ and III’ at 828 and 840 cm^−1^, respectively). According to the simulated spectra, these two bands are assigned to the same carboxylate wagging modes of bands II and III, oscillating at a slightly higher frequency due to their different chemical environment after the phase transition occurs. Once proven that the doublets of IR bands II, III and II’, III’ identify the as‐synthesized and evacuated phase, respectively, it is important to highlight that the coexistence of these two phases during the phase transition clearly classifies it as a first‐order transition.^[^ ^50^ ^]^ This finding implies that the proposed conformational change of linker A, associated with the phase transition, is an activated process. As a last remark, the computed Δ*G*_ads_ of −32.5 kJ mol^−1^ per H_2_O molecule is consistent with a hydrogen bonding interaction and justifies the room temperature reversibility of the whole process.

### 2.4 Solid‐State NMR Spectroscopy

**Figure 4:**
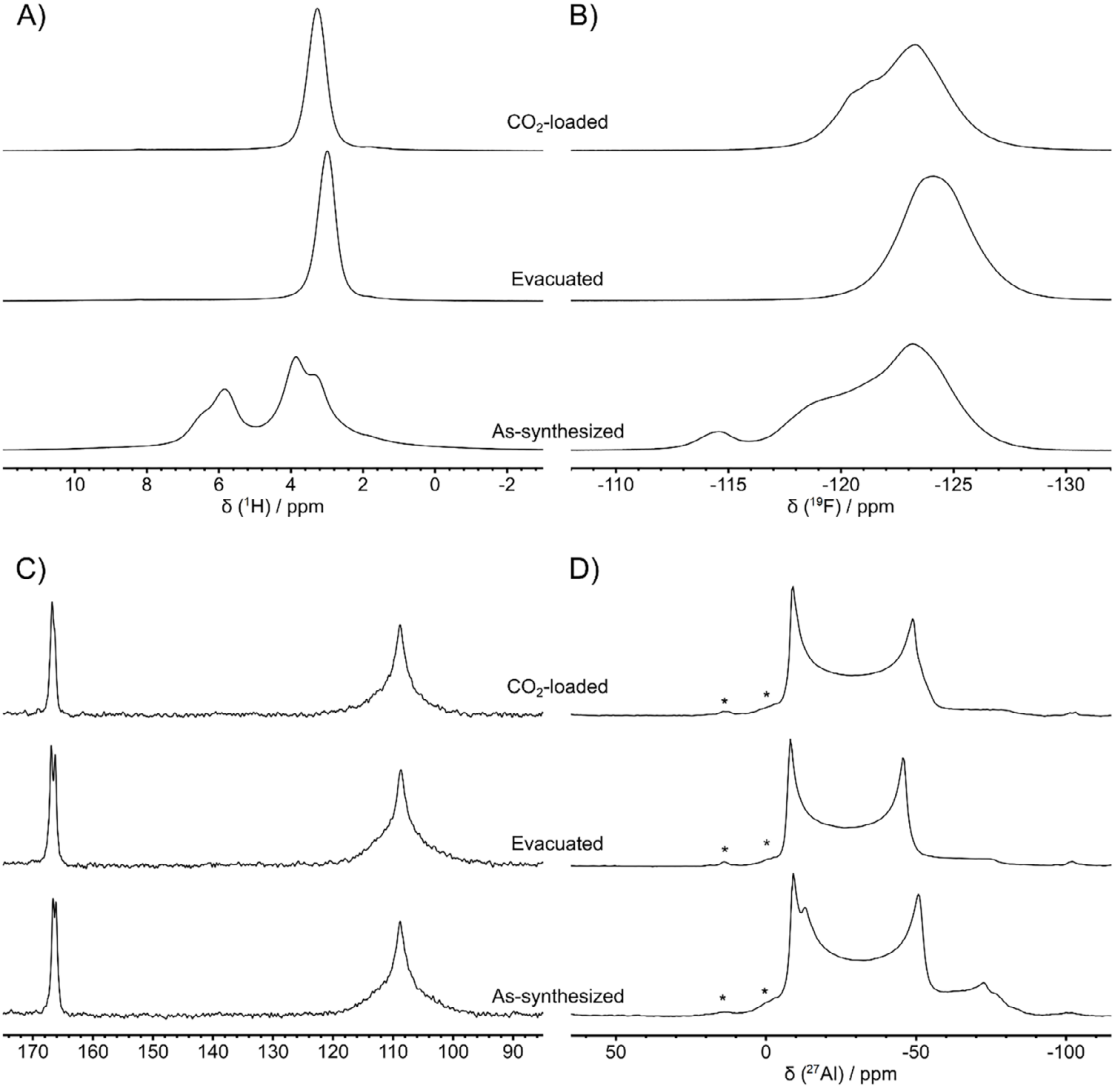
A) ^1^H DE MAS spectra, B) ^19^F DE MAS spectra, C) ^19^F‐^13^C CP MAS spectra, and D) ^27^Al DE MAS spectra of as‐synthesized, evacuated, and CO_2_‐loaded Al‐TFS. Asterisks in panel D indicate Al hydroxide/oxide impurities.^[^ ^26^ ^]^ All spectra were recorded at a spinning frequency of 15 kHz.

To obtain further insight at the atomic level into the structure of as‐synthesized and evacuated Al‐TFS, solid‐state NMR (SSNMR) spectroscopy experiments were carried out exploiting ^1^H, ^19^F, ^13^C, and ^27^Al nuclei. As shown in Figure 4A, the ^1^H DE MAS (direct excitation magic angle spinning) spectrum of evacuated Al‐TFS shows a single isotropic signal at 2.99 ppm, ascribable to the μ_2_‐OH group bridging Al atoms in the structure, whereas four different signals (with isotropic chemical shifts, δ_iso_, of 3.30, 3.90, 5.82, and 6.45 ppm) are observed in the ^1^H spectrum of as‐synthesized Al‐TFS, arising from protons of μ_2_‐OH groups and water molecules experiencing hydrogen bonds. The presence of structural water molecules in as‐synthesized Al‐TFS also results in evident changes of the ^19^F and ^27^Al DE MAS spectra (Figure 4B,D). As far as ^19^F spectra are concerned, a single signal is observed for evacuated Al‐TFS (Figure S23, Supporting Information) with the isotropic peak centered at −124.1 ppm, whereas multiple signals are found for the as‐synthesized MOF with isotropic peaks in the −128 to −112 ppm spectral region (Figure S23, Supporting Information; Figure 4B), ascribable to fluorine atoms differently interacting with H_2_O molecules. ^27^Al DE MAS spectra of both Al‐TFS phases show signals typical of octahedrally coordinated Al,^[^ ^51^ ^]^ with spectral features arising from a single environment for the evacuated phase and from multiple environments for the as‐synthesized one (Figure 4D). The deconvolution of the ^27^Al spectrum of evacuated Al‐TFS (Figure S24, Supporting Information) gives a δ_iso_ value of 4.2 ppm and a quadrupolar coupling constant (C_Q_) of 9.57 MHz, with null asymmetry parameter (η_Q_). On the other hand, for as‐synthesized Al‐TFS three sub‐spectra are necessary to reproduce the experimental spectrum (Figure S24, Supporting Information), featured by δ_iso_ of 4.1, 4.4, and 5.1 ppm and C_Q_ of 10.02, 11.90, and 12.20 MHz, respectively, and η_Q_ = 0. The chemical shift and quadrupolar interaction parameters determined for evacuated Al‐TFS are similar to those reported for F_4_‐MIL53(Al).^[^ ^43^ ^]^ Hydration results in larger C_Q_ values for the as‐synthesized MOF, in agreement with data reported for Al‐FUM and MIL‐53(Al).^[^ ^14^, ^26^, ^52^ ^]^ The multiple ^27^Al environments detected for as‐synthesized Al‐TFS are most probably associated to Al sites in sample regions with different degree of hydration. Slight changes in ^13^C chemical shift are also observed by looking at the ^19^F‐^13^C (Figure 4C) and ^1^H‐^13^C (Figure S25, Supporting Information) cross polarization (CP) MAS spectra of the two Al‐TFS phases. In particular, for both evacuated and as‐synthesized Al‐TFS, ‐CF_2_‐ carbons give a broad signal centered at δ_iso_ = 108.8 ppm, while carboxylic carbons show two signals at δ_iso_ of 166.1 and 166.6 ppm for the as‐synthesized MOF and at δ_iso_ = 166.3 and 166.9 ppm for the evacuated one. These findings suggest a slightly different coordination of carboxylate groups, ascribable to Al‐TFS flexibility and/or crystallographic inequivalence of linkers. On the other hand, the small shift of carboxylate carbon signals together with the observation of null η_Q_ for the ^27^Al quadrupolar tensor suggest the absence of significant conformational changes of the carboxylate groups due to interactions with water in the as‐synthesized sample. The higher relative intensity of the carboxylate group signal(s) with respect to that of the ‐CF_2_‐ signal in ^1^H‐^13^C CP MAS spectra (Figure S25, Supporting Information) results from the shorter distance between ^1^H nuclei in μ_2_‐OH groups and carboxylate carbons.

Multinuclear SSNMR spectroscopy was also employed to highlight structural changes induced by CO_2_ adsorption by recording ^1^H, ^19^F, ^13^C, and ^27^Al experiments on an Al‐TFS sample loaded with CO_2_ at 1 bar pressure at room temperature. A downfield shift of ^1^H and ^19^F isotropic signals is observed upon CO_2_ adsorption, whereas the ‐CF_2_‐ ^13^C signal remains practically unchanged and signals of carboxylic carbons are slightly shifted to δ_iso_ values of 166.4 and 166.8 ppm (Figure 4; Figures S23 and S25, Supporting Information). Adsorption of CO_2_ affects the ^27^Al spectrum too (Figure 4D; Figure S24, Supporting Information), which still shows a signal typical of an octahedral environment but with a δ_iso_ value of 4.1 ppm and a quadrupolar coupling constant (C_Q_) of 9.87 MHz, with null asymmetry parameter (η_Q_). The increase of C_Q_ compared to evacuated Al‐TFS can be ascribed to a reduction of symmetry of the Al environment. These findings suggest that CO_2_ adsorption is associated with a rearrangement of the MOF structure, mainly affecting the OH groups bridging Al atoms, which also influences the Al environment, as well as the fluorine atoms of the linkers. It must be noted that, although strong interactions were detected between the μ_2_‐OH groups of Al‐FUM and CO_2_, leading to anisotropic dynamics of the gas inside the MOF, no changes of the Al environment were observed.^[^ ^53^ ^]^ The different behavior of Al‐TFS upon CO_2_ adsorption compared to Al‐FUM could be attributed to the flexible nature of its framework. However, at room temperature, the observed interactions of CO_2_ with the μ_2_‐OH groups and linkers of Al‐TFS are not strong enough to enable magnetization transfer from ^1^H or ^19^F nuclei of the framework to ^13^C nuclei of CO_2_ molecules in ^1^H‐^13^C or ^19^F‐^13^C CP MAS spectra, respectively. Since polarization transfer occurs through dipolar interactions, CP spectra can be employed to gather information on molecular dynamics and spatial proximity between nuclei. At short contact times, only ^13^C close in space to ^1^H (or ^19^F) nuclei are detected, whereas at longer contact times polarization transfer occurs across longer internuclear distances. However, this transfer is not observed for molecular fragments subjected to fast molecular dynamics, which modulates and weakens the dipolar interactions. In our case, the signal of CO_2_^13^C is not observed in the CP MAS spectra of CO_2_‐loaded Al‐TFS, even at long contact time (8 ms), whereas it is detected in the ^13^C DE MAS spectrum (Figure 4C; Figures S25, Supporting Information). This behavior is most likely due to the highly dynamic motion of CO_2_.

### 2.5 Textural Properties of Al‐TFS

**Figure 5:**
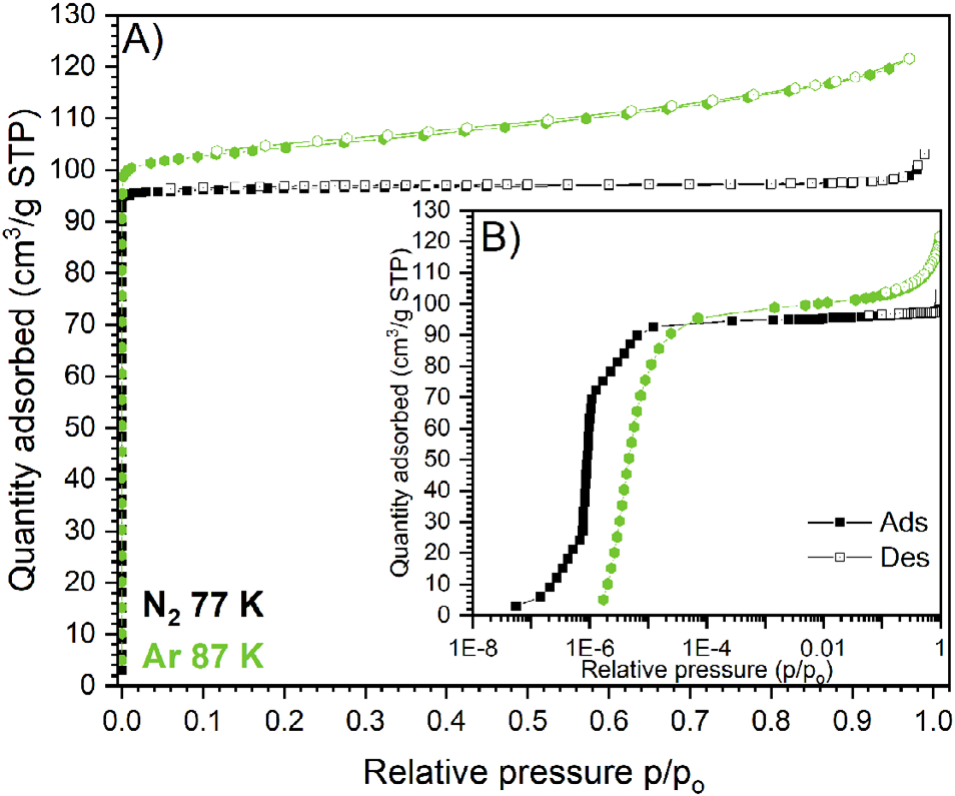
Adsorption/desorption isotherms of Al‐TFS collected using N_2_ (black curve) and Ar (green curve) at 77 and 87 K, respectively. A) Linear scale; B) Semi‐logarithmic scale.

The characterization of the textural properties of Al‐TFS was performed by measuring volumetric adsorption isotherms using N_2_ and Ar at 77 and 87 K, respectively (Figure 5). Since the sample is activated to remove physisorbed water, as previously reported, the starting point before each adsorption measurement is the evacuated phase. At first glance, both isotherms can be classified as Type I(a), typical of ultra‐microporous materials. However, by observing the plot in semi‐logarithmic scale (Figure 5B), a change in the slope of the N_2_‐isotherm at ≈10^−6^ p/p_o_, which may indicate a possible conformational change of the framework, is evident. It is worth noting that the step‐shaped isotherm is only induced by N_2_ adsorption, whereas the totally inert Ar exhibits a standard type I(a) isotherm. The computed BET specific surface areas (linear fit reported in Figure S26, Supporting Information) are 417 ± 5 m^2^·g^−1^ (for N_2_ at 77 K) and 378.8 ± 0.2 m^2^·g^−1^ (for Ar at 87 K). The BET fits of the two isotherms were validated by the “BET surface Identification” (BETSI) software.^[^ ^54^ ^]^ The IUPAC report of 2015 recommends using Ar when the surface presents functional groups or narrow micropores, since the quadrupolar moment of N_2_ molecules can orient differently upon interaction with the surface, leading to an overestimation of the monolayer capacity and thus of the BET area.^[^ ^55^ ^]^ In a recent paper, Datar et al. reported that MOFs with relatively low surface area (<1500 m^2^·g^−1^) have the highest deviation between the BET areas calculated from Ar and N_2_, and often those materials cannot satisfy all four Rouquerol consistency criteria for BET model applicability.^[^ ^56^ ^]^ This is confirmed for Al‐TFS, since the BET specific surface area calculated from the N_2_ isotherm at 77 K meets only three out of four criteria. For this reason, the BET specific surface area derived from the Ar isotherm at 87 K is to be considered the most accurate. The Al‐TFS specific surface area computed with Ar at 87 K is much lower than that of Al‐FUM (379 m^2^·g^−1^ vs 880 m^2^·g^−1^).^[^ ^57^ ^]^ Such difference can be easily noticed out by comparing the Ar isotherms of the two MOFs (Figure S27, Supporting Information). The specific surface areas of both Al‐TFS phases were also evaluated theoretically using the *Zeo++* software. A screening with different probe diameters was carried out (see Table S2, Supporting Information), highlighting a good agreement between the experimental and the theoretical results using a probe with a diameter of 2.8 Å.

The pore size distribution (PSD) of Al‐TFS was tentatively derived by applying the NL‐DFT method to the experimental Ar adsorption isotherm and selecting the pore geometry and the fitting model with the lowest standard deviation. PSD and cumulative pore volume (CPV) plots are reported in Figure S28 (Supporting Information), together with the goodness of fit curve. Although the overlap of theoretical and experimental isotherms is poor (Figure S28, Supporting Information, right), the corresponding PSD still detects a unique family of micropores of 3.7 Å, with a total pore volume of 0.13 cm^3^·g^−1^. Again, the experimental result was supported by theoretical data obtained from *Zeo++*. Indeed, PSD and CPV of both Al‐TFS phases were calculated with a probe diameter of 2.8 Å (Figure S29, Supporting Information). Both experimental and theoretical calculations identified a unique family of micropores. *Zeo++* was also used to predict the textural properties of Al‐FUM, recognizing a unimodal distribution of micropores with a larger size (5.5 Å). The smaller micropore size established for Al‐TFS is reasonable, considering the steric hindrance of the fluorine atoms pointing toward the center of the pores. It is noteworthy that the textural properties of Al‐TFS differ significantly from those of Al‐SUC. The latter MOF exhibits a hysteretic Type IV isotherm with N_2_ at 77 K and a pore size of ≈5 nm, indicative of a MOF with a mesoporous structure.^[^ ^45^ ^]^

### 2.6 CO_2_ Sorption Properties of Al‐TFS

**Figure 6:**
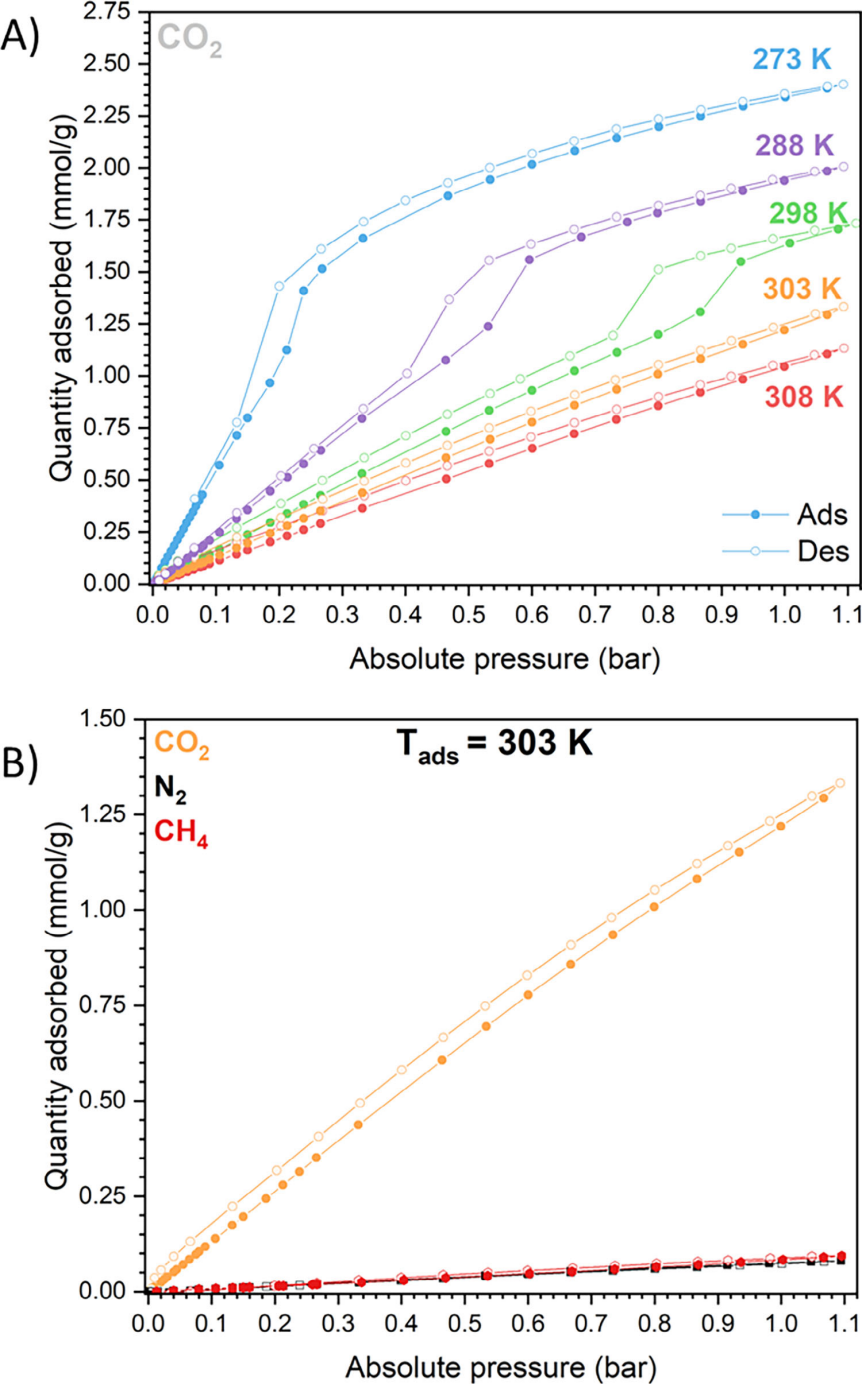
A) Adsorption/desorption CO_2_ isotherms of Al‐TFS collected in the 273–308 K temperature range (0–1 bar); B) Comparison between adsorption measurements performed at 303 K using CO_2_ (orange), N_2_ (black) and CH_4_ (red) as adsorptive.

**Figure 7:**
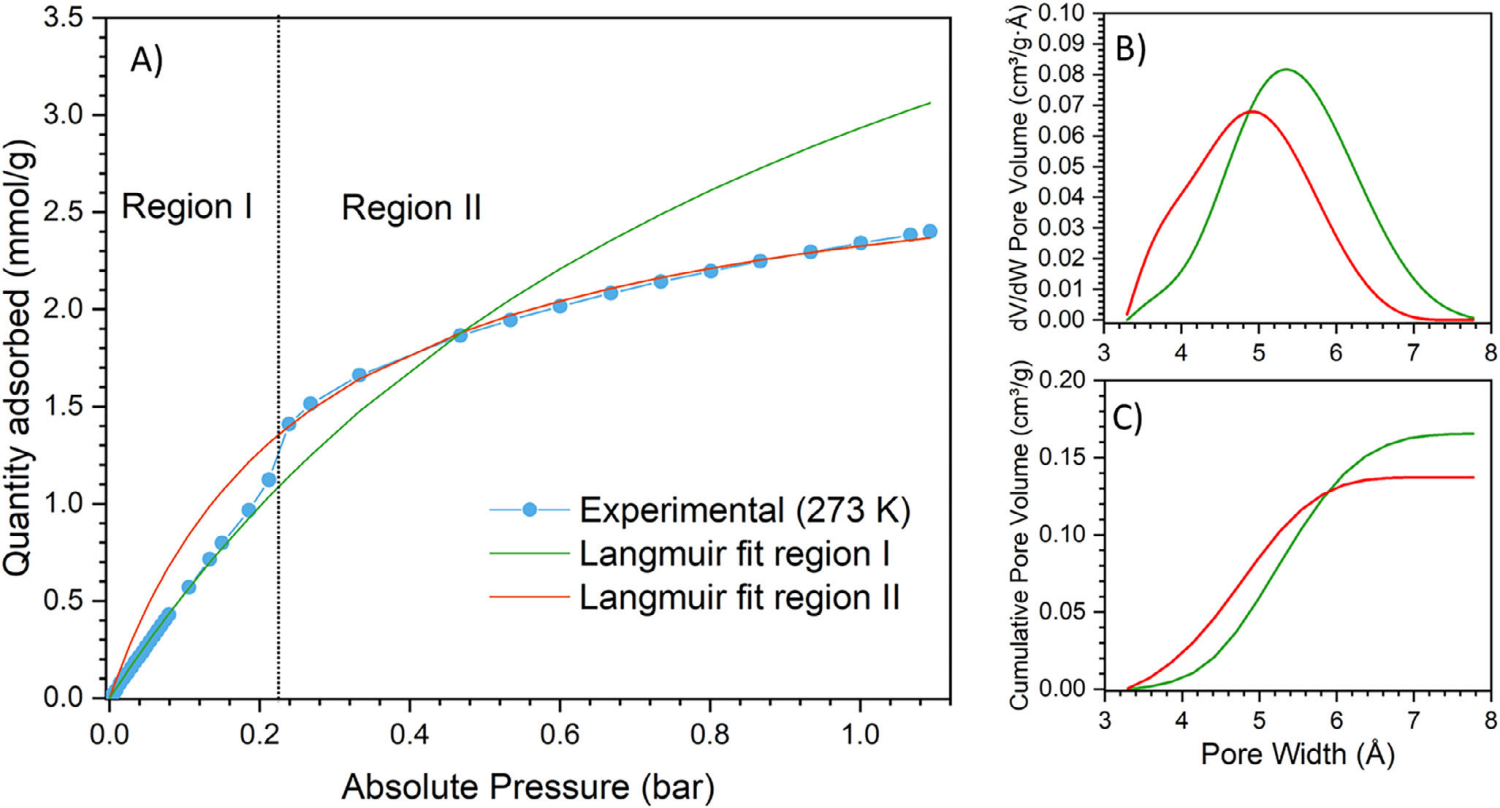
A) CO_2_ adsorption isotherm (light blue curve and symbols) separated into two regions (red and green curves) fitted with two different SSL equations. PSD (B) and CPV (C) curves obtained by NL‐DFT of the fitted isotherms in region I (green) and II (red) of the experimental curve.

The CO_2_ adsorption capacity of Al‐TFS was evaluated in the 0–1 bar range by collecting consecutive adsorption/desorption isotherms at different temperatures (273–308 K). The sequence of CO_2_ isotherms is reported in Figure 6A. At 1 bar and 273 K, the CO_2_ uptake is ≈2.4 mmol·g^−1^, and it decreases by increasing the temperature, remaining above 1 mmol·g^−1^ at 303 K. The total CO_2_ uptake of Al‐TFS at 1 bar and 273 K is lower compared to the one reported in the literature for Al‐FUM (3.7 mmol·g^−1^).^[^ ^58^ ^]^ However, the whole set of CO_2_ isotherms has a peculiar shape and requires careful discussion. The isotherm collected at the lowest temperature (273 K) exhibits a step at ≈0.3 bar with a moderate hysteresis. Two main regions can be identified: i) a linear range up to 0.2 bar and ii) a Langmuir‐type region, not approaching a plateau at these working conditions. By fitting the two regions with two different single‐site Langmuir equations, the corresponding interaction energy can be estimated by calculating the Henry's constants – *K*_H_ (see Figure 7A; Table S3, Supporting Information). As expected, the CO_2_ interaction strength is higher in region II (above 0.3 bar). Given the Al‐TFS flexibility already exhibited upon water removal, the peculiar step‐shaped trend of CO_2_ isotherms can be due to an analogous deformation of the evacuated phase triggered by CO_2_ adsorption, as suggested by the SSNMR measurements on CO_2_ loaded Al‐TFS. By increasing the temperature of the analysis, the isotherm step moves toward higher absolute pressures and the hysteresis becomes even more pronounced (see isotherms at 288 and 298 K in Figure 6A). At 303 K, the linear region of the isotherm (region I) dominates the entire pressure range. The shift of the isotherm inflection point toward higher pressures by increasing the temperature agrees with what is reported for other flexible MOFs, whose deformation is induced by adsorption.^[^ ^17^, ^59^, ^60^ ^]^

Since Al‐TFS is characterized by ultra‐micropores, CO_2_ at 273 K may be a good alternative to Ar at 87 K for the PSD evaluation using NL‐DFT.^[^ ^55^ ^]^ Figure S30 (Supporting Information) shows that, even with a good agreement between the experimental and the theoretical isotherms, the PSD plot reveals a trimodal distribution, consistent with the existence of three different families of micropores. This outcome does not agree with the results obtained by both the structure refinement and the *Zeo++* software. The discrepancy could arise from the non‐existence of proper kernels of theoretical isotherms to describe the pore size modification induced by adsorption. For this reason, the two curves obtained by the fitting of regions I and II of the experimental isotherm (green and red curves in Figure 7A) were used as input to simulate the pore width and the accessible volume of both Al‐TFS phases by NL‐DFT. The PSD and CPV plots reported in Figure 7B,C (and the corresponding NL‐DFT goodness of fit graph reported in Figure S31, Supporting Information) suggest a slight contraction of the micropores in region II, namely after the change in the slope of the isotherm (*p* > 0.2 bar). Furthermore, the presence of a moderate hysteresis in the adsorption/desorption isotherms at 273 K (Figure 6A) proves that the phase transition induced by CO_2_ adsorption involves a modification of the cell volume, as already detected by PXRD for the water‐induced transition.

A PXRD pattern of Al‐TFS under 1.7 bar CO_2_ was therefore collected at room temperature, finding that the adsorption of CO_2_ does induce a structural rearrangement, responsible for the step in the isotherm. The resulting phase, named “CO_2_‐loaded Al‐TFS,” displays a PXRD pattern that differs from both those of the as‐synthesized and evacuated phases (Figure S32, Supporting Information). Indexing of the PXRD pattern of CO_2_‐loaded Al‐TFS suggested a monoclinic unit cell, whose lattice parameters were refined with the Pawley method (Figure S33, Supporting Information) in the *P*2_1_ */n*, space group obtaining a Rwp value of 5.06: *a* = 13.913(1) Å, *b* = 6.6513(5) Å, *c* = 9.874(1) Å, *β* = 90.797(3)°, volume = 913.7(1) Å^3^. The lattice parameters suggest that this phase is similar to the evacuated Al‐TFS, with a slight expansion of the *a* axis and a slight shrinkage of the *c* axis, and that major conformational rearrangements can be excluded upon adsorption of CO_2_. The cell volume of CO_2_‐loaded Al‐TFS is slightly smaller than that of evacuated Al‐TFS (913.7 vs 922.1 Å^3^, respectively, corresponding to a 0.9% contraction upon CO_2_ adsorption), in line with the previously discussed apparent shrinking of pores above the step in the CO_2_ isotherm. The subtle structural rearrangements induced by CO_2_ adsorption are further supported by the spectral changes observed by multinuclear SSNMR experiments.

The CO_2_ isosteric heat of adsorption (Q_st_) was computed (Figure S34, Supporting Information) by applying the Clausius–Clapeyron equation to the adsorption isotherms at different temperatures (298, 303, and 308 K). The adsorption enthalpy Δ*H*_ads_ = −Q_st_ was then calculated from the linear regression of the ln(p) versus 1/T plot. The resulting average Q_st_ value is slightly higher (29.0 kJ mol^−1^) than that reported in the literature for Al‐FUM (23.0 kJ mol^−1^).^[^ ^61^ ^]^ This is in good agreement with other examples of fluorinated MOFs, where the dipolar character of C‐F bonds increases the affinity toward CO_2_, compared to the hydrogenated counterparts.^[^ ^36^, ^43^ ^]^

CO_2_/CH_4_ and CO_2_/N_2_ selectivity values were also estimated at 303 K based on IAST, predicting multi‐component isotherms starting from the experimental single‐component ones reported in Figure 6B. The selected mixtures are: i) biogas (50% CO_2_ – 50% CH_4_) and ii) coal‐derived flue gas (15% CO_2_ – 85% N_2_). For both gas compositions, Al‐TFS has a selectivity between 15 and 20 (Figure S35, Supporting Information, left). CO_2_/N_2_ selectivity of Al‐TFS and Al‐FUM was compared, employing the N_2_ isotherm of Al‐FUM at 303 K reported in the literature.^[^ ^30^ ^]^ As shown in Figure S35 (Supporting Information), both MOFs are characterized by rather poor selectivity values, which may limit their applications in real carbon capture operations. However, the IAST calculation revealed that, at low pressure, Al‐FUM has a higher selectivity, but over 0.7 bar the trend reverses. It is worth noting that, at 303 K, the partial pressure of CO_2_ in the simulated mixtures is below the step of the isotherm. For this reason, if the CO_2_ partial pressure exceeds the position of the step, i.e., in a pressure swing adsorption process, the selectivity could increase.

### 2.7 H_2_O Sorption Properties of Al‐TFS

**Figure 8:**
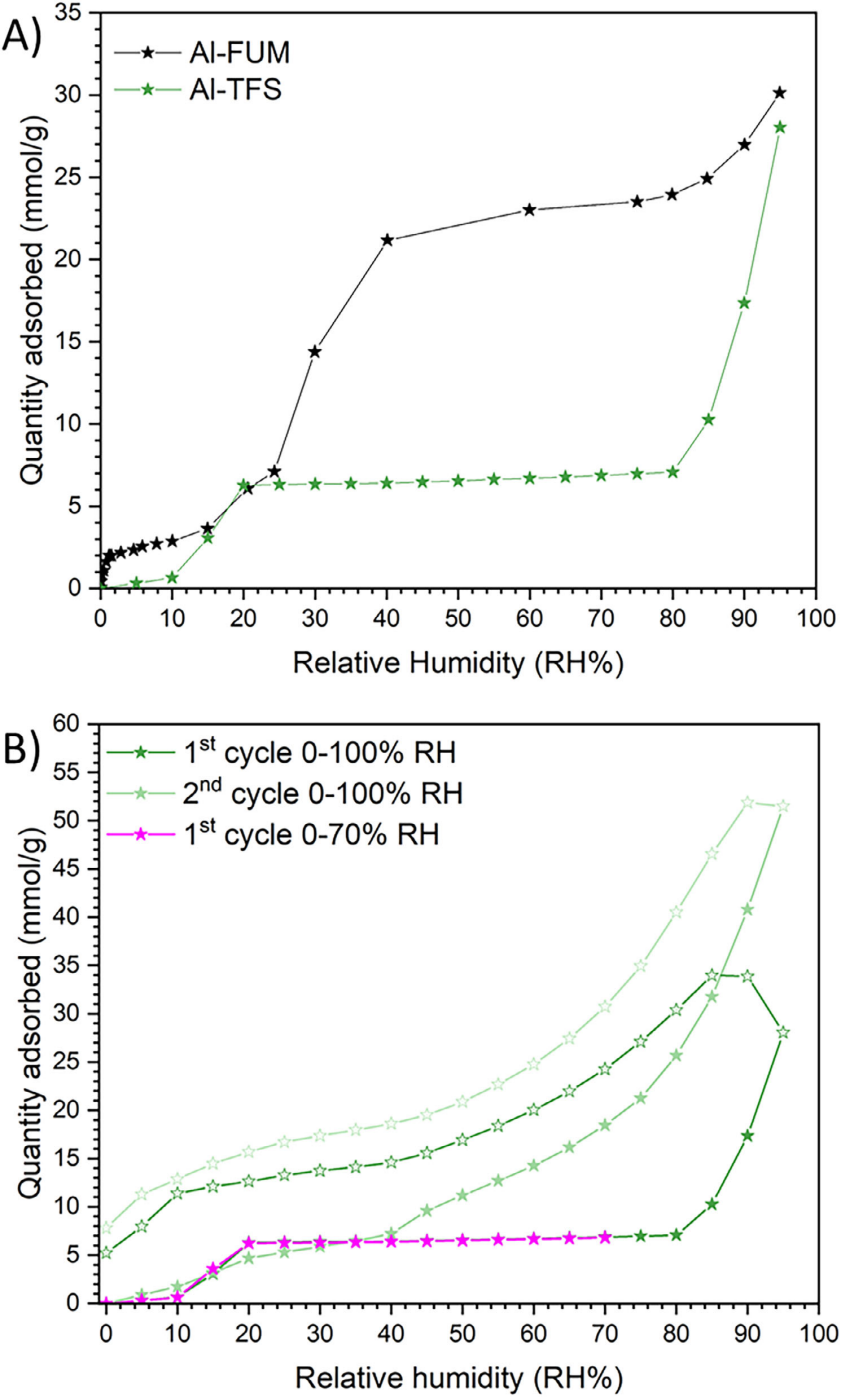
A) Comparison between H_2_O adsorption isotherms of Al‐FUM^[^ ^62^ ^]^ (black curve) and Al‐TFS (green curve) at 303 K. B) H_2_O adsorption (full stars) and desorption (empty stars) isotherms collected at 303 K on Al‐TFS. Primary (first cycle) and secondary (second cycle) isotherms obtained in the whole (0–100%) relative humidity range (dark green and light green curves, respectively); primary (first cycle) isotherm performed up to 70% of relative humidity (magenta curve). Please note that the desorption curve (magenta empty stars) is not visible since totally superimposed to the adsorption isotherm (magenta full stars).

H_2_O adsorption properties of Al‐TFS were also investigated. Figure 8A reports the comparison between the H_2_O isotherms of Al‐FUM and Al‐TFS at 303 K. As reported in the literature, Al‐FUM displays a peculiar H_2_O isotherm with a two‐step profile (see black curve in Figure 8A), characterized by a first interaction probably involving the organic linker and a second adsorption after 0.2 p/p_o_, where water clustering starts.^[^ ^62^, ^63^ ^]^ This behavior turns into a large adsorption capacity at intermediate relative pressures (≈24 mmol·g^−1^ at 40% of RH), an easy desorption and a good stability upon several sorption/desorption cycles.^[^ ^62^ ^]^ In contrast, the H_2_O isotherm of Al‐TFS (green curve) exhibits a single step at ≈0.1–0.2 p/p_o_ and a constant uptake of ≈5 mmol·g^−1^ up to 0.8 p/p_o_ (RH 80%), where a steep increase of the adsorbed amount occurs. As observed in Figure 8B, for Al‐TFS neither the desorption branch of the first adsorption cycle until 100% of RH (empty green curves) nor the secondary adsorption/desorption isotherm (full and empty light green curve) coincide with the adsorption profile of the primary isotherm in the whole RH range, suggesting that the material is not stable upon H_2_O adsorption until 100% of RH.

To further investigate the material stability upon H_2_O adsorption, a new (primary) adsorption/desorption isotherm was collected at 303 K only up to 70% of RH (magenta curve in Figure 8B), i.e., before the steep final increase of the adsorbed amount observed in the primary adsorption isotherm of Figure 8A. The lack of hysteresis in this isotherm suggests that Al‐TFS is stable upon water adsorption only if RH does not exceed 70%. The stability of Al‐TFS in different RH conditions was also investigated by performing PXRD after each sorption analysis. Figure S36 (Supporting Information) shows that the Al‐TFS structure is stable upon H_2_O adsorption in the 0–70% RH range, whereas a general loss of crystallinity (proved by a clear peak broadening and a reduction of the signal/noise ratio) is observed after H_2_O adsorption in the 80–100% RH region. At such high RH values, the sample likely undergoes a partial amorphization. The lower stability to humidity of Al‐TFS compared to Al‐FUM is likely due to the much higher acidity of H_2_TFS (pK_a1_ estimated to be ≈0.4, based on the value reported for the structurally similar perfluoropropanoic acid)^[^ ^64^ ^]^ than that of H_2_FUM (pK_a1_ = 2.9).^[^ ^65^ ^]^ This higher acidity translates into a significantly weaker coordination strength of the corresponding TFS^2‐^ conjugate base, making the COO‐Al bonds in Al‐TFS more susceptible to hydrolysis. These results reveal that Al‐TFS, despite having fluorine‐lined pores, is hydrophilic and becomes saturated above 20% RH. The complete saturation observed at low RH can be ascribed to the presence of Al‐OH groups confined within narrow, hydrophilic, ultra‐microporous pockets compared to the same groups pointing at the larger cavities of Al‐FUM, which probably create a less confined environment.

## 3 Conclusion

In this paper, a novel perfluorinated Al‐based MOF (Al‐TFS) was synthesized for the first time, following a simple “shake and bake” route. The formation of crystalline Al‐TFS involves two steps: i) formation of an intermediate phase; ii) precipitation of the crystalline MOF upon washing. Through the complementary use of different characterization techniques, the main features of Al‐TFS were revealed and compared to those of its protonated topological analog Al‐FUM (commercially known as Basolite A520).

The fluorinated aliphatic chains decorating the walls of Al‐TFS channels are responsible for major differences in the textural properties and adsorption behavior compared to Al‐FUM. Indeed, due to the steric hindrance of fluorine atoms, Al‐TFS is characterized by narrower pores with an unexpected, peculiar adsorption‐induced flexible behavior. The peculiar inflection point detected on the CO_2_ isotherms reveals a slight contraction of the micropore size and volume, as confirmed by the application of the NL‐DFT method to the experimental data. This behavior is supported by the structural changes detected in the PXRD patterns collected in CO_2_ atmosphere. The CO_2_‐induced Al‐TFS flexibility may be exploited in terms of CO_2_/N_2_ selectivity that, in contrast to Al‐FUM, increases at higher gas pressure.

Water has also an impact on the stable crystalline phase of Al‐TFS, which expands when the material is completely evacuated (no traces of water adsorbed). However, stability to water is a weak point for Al‐TFS, since it undergoes a partial amorphization when RH exceeds 70%.

The synthesis and advanced characterization of the new Al‐TFS is yet another step in our quest to explore the world of perfluorinated MOFs, further contributing to the understanding of the role of fluorination in endowing the framework with peculiar adsorption properties.

## 4 Experimental Section

### Materials

All chemicals are commercially available and used as received from the specified vendors without further purification. Aluminum nitrate nonahydrate (Al(NO_3_)_3_⋅9H_2_O) and tetrafluorosuccinic acid (H_2_TFS) were purchased from Honeywell Fluka and from Fluorochem, respectively. Acetone was purchased from Carlo Erba.

### Synthesis

750 mg (2 mmol) of Al(NO_3_)_3_·9H_2_O and 360 mg (2 mmol) of H_2_TFS were mixed in powder form in a polytetrafluoroethylene (PTFE, Teflon) hydrothermal reactor. The bomb was heated in a static oven at 393 K for 24 h. The obtained white powder was washed twice using deionized water (20 mL) for 15 min and once in acetone (20 mL) for 10 min. The recovered product was dried overnight in a static oven at 333 K. Yield: 218 mg (44%). ICP‐OES analysis: Al wt% obs 8.09%, calc 10.42%.

### Powder X‐Ray Diffraction (PXRD)

The powder X‐ray diffraction pattern of as‐synthesized Al‐TFS was collected in reflection geometry in the 2−90° 2θ range, with a 115.20 s·step^−1^ counting time and with a step size of 0.025° on a BRUKER D8 ADVANCE diffractometer, equipped with an LYNXEYE XE‐T detector and using a Cu‐K_α_ radiation source. The ceramic tube operated at 40 kV and 40 mA. The PXRD patterns in controlled atmosphere were collected on a PANalytical X′Pert diffractometer using Cu Kα radiation transmission geometry and equipped with an X'celerator strip detector. The sample was loaded in a borosilicate glass capillary (internal diameter 0.8 mm). The diffractogram of evacuated Al‐TFS was recorded after degassing the capillary with a vacuum pump (residual pressure < 10^−3^ mbar) and heating up to 393 K overnight. In a second capillary, 1.7 bar of CO_2_ was dosed on evacuated Al‐TFS.

### Field Emission Scanning Electron Microscopy (FE‐SEM)

FE‐SEM images were obtained using a LEO 1525 Gemini SEM (Zeiss/LEO) with an acceleration voltage of 5.00 kV. The sample was sputter‐coated under vacuum with chromium.

### Thermogravimetric Analysis (TGA)

Thermogravimetric analysis (TGA) was performed using a Netzsch STA 2500 Regulus thermoanalyzer under air atmosphere (N_2_:O_2_ = 70:30) and with a heating rate of 5 K·min^−1^.

### Inductively Coupled Plasma Optical Emission Spectroscopy (ICP‐OES)

ICP analysis was carried out using a Varian 700‐ES series. A calibration curve was obtained with four standard aluminum solutions (0.5, 1.0, 2.5, and 5.0 mg·L^−1^, respectively). The sample was prepared dissolving 4.4 mg of MOF in 3.8 mL of concentrated nitric acid (≈65% wt.) and later diluted to 100 mL in a volumetric flask with deionized water.

### Quantitative Solution ^19^F Nuclear Magnetic Resonance (NMR) spectroscopy

Solution ^19^F NMR spectroscopy was carried out at 298 K on a Jeol JNM‐ECZ500S spectrometer equipped with a RoyalProbe Broadband probe. For the analysis, ≈15 mg of solid was introduced into a glass vial and kept at 393 K for 2 h. Afterward, the vial was capped while still hot and weighed to determine the mass of the desolvated solid (the cap had previously been weighed alongside the vial). 1.0 mL of 1 mol·L^−1^ NaOH in D_2_O, spiked with 0.1 mol·L^−1^ 2,6‐difluorobenzoic acid as a ^19^F internal standard, was then added to the vial and the mixture briefly sonicated and left to digest overnight. The NMR tube was then loaded with the solution, taking care to avoid transferring solid particles in the tube. The ^19^F NMR spectrum was acquired with a relaxation delay of 25 s, accumulating 4 scans.

### Structure Solution and Refinement

PXRD patterns of as‐synthesized, evacuated and CO_2_‐loaded Al‐TFS were indexed by using the program *TREOR*.^[^ ^66^ ^]^ Structure determination of as‐synthesized Al‐TFS was then carried out by using the parallel tempering algorithm implemented in the program *FOX*.^[^ ^47^ ^]^ Two crystallographically independent AlO_6_ octahedra were used as building units. Al atoms of the octahedra were fixed in two special positions with coordinates 0.0, 0.5, 0.5 and 0.5, 0.5, 0.5, respectively, corresponding to inversion centers. The rotational degrees of freedom around the octahedra were maintained to correctly orient the oxygen atoms. Two crystallographically independent molecular fragments of the linker were also included, whose centers of mass were fixed at special positions with coordinates 0.5, 0.0, 0.5 and 0.5, 0.5, 0.0, respectively, corresponding to inversion centers. The following anti‐bump restraints were set: Al‐O = 1.8 Å; Al‐C = 2.5 Å; Al‐F = 3.8 Å; O‐O = 1.8 Å; O‐F = 2.4 Å; C‐C = 1.5 Å; F‐F = 1.9 Å. The scale of the anti‐bump restraints was set to 1000. During the optimization procedure, the atoms belonging to the crystallographically equivalent fragments originating from the inversion center were merged with each other by the “dynamical occupancy correction” function, thus ensuring that the stoichiometry and electroneutrality of the structure were respected.^[^ ^48^, ^67^ ^]^ The crystal structure of the as‐synthesized phase was refined by using the program *TOPAS Academic V6*.^[^ ^48^ ^]^ Background, cell parameters, diffractometer zero error and profile function were first refined. The structural model was refined by using distance and angle restrains. The following restraints on the bond lengths were used: Al‐O = 1.95 Å, C‐C = 1.55 Å, C‐O = 1.27 Å, C‐F = 1.35 Å. The statistical weight of these restraints was maintained high at the first stages of the refinement and then progressively decreased. The asymmetric unit contains two independent half‐fragments of the linker that are generated for symmetry by the inversion point in (0.5, 0.0, 0.0) to form the complete linker. Two water molecules were found by using the Fourier map function in *TOPAS* to identify the residual electron density. One of these water molecules sits on a special position with coordinates 0.5, 0.0, 0.0, corresponding to an inversion center. The positions of water molecules were then allowed to refine freely. The thermal factors (beq) were constrained in three independent groups, one for the light atoms (C, O, F), one for the Al atoms and one for water and left free to refine. To better model the intensity of some reflections, spherical harmonics (order 4) were introduced in the refinement. At the end of the refinement, the shifts in all parameters were less than their standard deviations. Crystallographic data are summarized in Table S1 (Supporting Information), while Rietveld plots are shown in Figure S8 (Supporting Information).

A similar approach was used for the evacuated phase; however, attempts to refine the structure with the Rietveld method led to strong distortions of the angles defining the [AlO_6_] octahedra and to some unrealistic Al‐O distances. The problems could not be solved even by using strong restraints or rigid body approaches. Therefore, the structure found with *FOX* was only used as starting model for DFT calculations.

### IR Spectroscopy

Transmission IR spectra were collected in the 5000–500 cm^−1^ spectral range, using a Bruker Vertex 70 spectrophotometer, equipped with a MCT (mercury cadmium tellurium) cryogenic detector. The resolution was 2.0 cm^−1^ and an average of 32 scans was used to increase the signal to noise ratio. Before the analysis, the sample, in form of self‐supported pellet, mechanically protected by a gold envelope, was inserted in a special home‐made quartz cell with KBr windows. The cell was connected to a conventional high‐vacuum glass line, equipped with mechanical and turbo molecular pumps (residual pressure *p* < 10^−4^ mbar). Consecutive spectra were recorded while outgassing the sample (delay between acquisitions: 180 s). Finally, the cell was connected to a second high‐vacuum glass line and inserted into an oven to activate the material overnight at 353 K (ramp 3 K·min^−1^).

### Diffuse Reflectance Infrared Fourier Transform (DRIFT)

DRIFT spectra were acquired in the 4000–600 cm^−1^ spectral range using a Bruker Invenio R spectrophotometer, equipped with a MCT (mercury cadmium tellurium) cryogenic detector. The resolution was 2.0 cm^−1^, and an average of 32 scans was used to increase the signal to noise ratio. The sample was inserted into a PIKE DIFFUSIRTM cell equipped with an environmental chamber that can be used up to 1273 K. The environmental cell was coupled with the PIKE Temperature Control Module and TempPRO software. The sample was diluted to 1% in Si powder to maximize the detected signal. Si was used as diluent and as reference (blank) instead of the more common KBr. Being highly hygroscopic, KBr absorbs moisture from the atmosphere very easily. Since the entire DRIFT experiment has been carried out by changing the relative humidity inside the environmental chamber, there would have been a mismatch between blank and sample spectra. The use of Si powder avoids this problem. To activate the sample, the DRIFT cell was connected to a conventional high‐vacuum glass line and the sample was heated up to 353 K (heating ramp of 3 K·min^−1^), while the instrument recorded the spectra. The time‐resolved DRIFT experiment has been carried out by keeping the same conditions described above, but reducing the average scan from 32 to 8 (acquisition time ≈8 s) during sample outgassing.

### Solid‐State NMR (SSNMR) Spectroscopy

SSNMR experiments were carried out on a Bruker Avance Neo spectrometer working at Larmor frequencies of 500.13, 470.59, 130.32, and 125.77 MHz for ^1^H, ^19^F, ^27^Al, and ^13^C nuclei, respectively, equipped with a 4 mm double‐channel (H/F‐X) cross polarization – magic angle spinning (CP‐MAS) probe. 90° pulse durations of 3.0, 3.2, and 3.8 µs were employed for ^1^H, ^19^F, and ^13^C, respectively. ^1^H and ^19^F spectra were recorded by Direct Excitation (DE), accumulating 32 scans with a recycle delay between consecutive transients of 1 s for evacuated Al‐TFS. For as‐synthesized Al‐TFS the recycle delay was 2 s for both ^1^H and ^19^F experiments. For CO_2_‐loaded Al‐TFS a recycle delay of 7 s and 8 s was used for ^1^H and ^19^F spectra, respectively.

For as‐synthesized and evacuated Al‐TFS, ^19^F‐^13^C CP experiments were performed under high power ^19^F‐decoupling using a contact time of 1 ms and a recycle delay of 1 s; 400 scans were accumulated for each spectrum. For the CO_2_‐loaded sample, ^19^F‐^13^C CP spectra were recorded with contact time values of 1 and 8 ms, a recycle delay of 5 s and 400 scans. ^1^H‐^13^C CP experiments were performed under high power ^1^H‐decoupling using a contact time of 8 ms and a recycle delay of 1 s for as‐synthesized and evacuated Al‐TFS, and 6 s for CO_2_‐loaded Al‐TFS, accumulating 2000 scans. A ^13^C DE MAS spectrum was recorded on CO_2_‐loaded Al‐TFS with a recycle delay of 5 s and accumulating 512 scans. ^27^Al DE spectra were acquired using an excitation pulse with a duration of 0.2 µs (i.e., a flip angle of π/6) and accumulating 4000 scans with a recycle delay of 1 s.

All spectra were recorded at room temperature under MAS conditions at a frequency of 15 kHz using air as spinning gas. For ^19^F experiments, spectra were recorded at multiple spinning frequencies to identify the isotropic peak. The chemical shift scale was referenced to the ^13^C signal of adamantane at 38.48 ppm and calculated from the same value for all the other nuclei using the unified scale recommended by IUPAC.^[^ ^68^ ^]^

The evacuated and CO_2_‐loaded Al‐TFS samples for SSNMR measurements were prepared using a home‐made cell provided with a mechanical lever operated from outside, enabling the capping of the rotor without disturbing the cell atmosphere. In the first case, Al‐TFS powder, packed into the NMR rotor (4 mm external diameter), was evacuated inside the cell by heating 6 h under vacuum (0.1 mbar) at a temperature of 393 K and then sealed. For the preparation of the CO_2_‐loaded sample, the cell containing the evacuated sample was loaded with CO_2_ at 1 bar pressure at RT, and the rotor was sealed after equilibration under the gas atmosphere. ^27^Al spectral line shapes were simulated using the solid line shape analysis tool “SOLA” of the Bruker software *TopSpin*.

### Adsorption/Desorption Isotherms

N_2_ isotherms were collected at 77 K using a Micromeritics 3FLEX sorption analyzer, using liquid nitrogen for the cooling bath. About 44 mg of powder was weighed and activated overnight at 353 K (heating ramp of 3 K·min^−1^). Brunauer–Emmett–Teller (BET) surface area was evaluated in the 1.5·10^−6^–1.6·10^−5^ p/p_o_ range by following the Rouquerol consistency criteria.^[^ ^69^ ^]^

Ar isotherm was collected at 87 K using a Micromeritics ASAP2020 sorption analyzer. The dewar was filled up with liquid argon. About 55 mg of sample was weighed and activated overnight at 353 K (3 K·min^−1^). The BET surface area was evaluated following the Rouquerol consistency criteria in the 6·10^−6^–8·10^−5^ p/p_o_ range.CO_2_, N_2_, and CH_4_ isotherms were collected at different temperatures (between 273 and 308 K) using a Micromeritics ASAP2020 sorption analyzer. To keep isothermal conditions for each analysis, the sample was inserted in a home‐made patented glass coating cell^[^ ^70^ ^]^ in which a coolant or heating fluid, connected to a thermostatic bath (JULABO F25), can recirculate. About 85 mg of sample was weighed and activated at 353 K (3 K·min^−1^) overnight.

In the adsorption/desorption isotherms of N_2_ at 77 K, Ar at 87 K and CO_2_ at 273 K, the p_0_ was experimentally determined by the sorption analyzer, using the dedicated saturation pressure tube.

Starting from the Ar and CO_2_ adsorption isotherms collected on Al‐TFS at 87 and 273 K, respectively, the pore size distribution (PSD) and the cumulative pore volume (CPV) of Al‐TFS were evaluated by applying the non‐local density functional theory (NL‐DFT) method by using the *MicroActive* Software provided by Micromeritics. For each experimental adsorption curve, the kernel of isotherms with the best fit was selected. In particular: i) for Ar at 87 K a cylindrical pore geometry was selected and the isotherm was fitted with the model “Ar@87‐Zeolites, H‐Form, NLDFT”; ii) for CO_2_ at 273 K a slit geometry was selected and the curves were fitted selecting the model “CO_2_@273 Carbon Slit Pores, 10 atm, NLDFT”. The textural properties of Al‐TFS were also evaluated theoretically using the software *Zeo++*.^[^ ^71^ ^]^ The accessible surface area (ASA) and non‐accessible surface area (NASA) were evaluated using different probe diameters from 2 to 3.4 Å (Ar kinetic diameter). PSD and CPV were evaluated using a pore diameter of 2.8 Å.

CO_2_/N_2_ and CO_2_/CH_4_ selectivity values were predicted starting from the experimental isotherms collected at 303 K for each probe gas using the ideal adsorbed solution theory (IAST), as implemented in the software *IAST++*.^[^ ^72^ ^]^ The CO_2_/N_2_ selectivity of Al‐FUM was predicted in the same way starting from single‐component isotherms available in the NIST database^[^ ^63^ ^]^ and reported in the paper of Coelho et al.^[^ ^30^ ^]^

Water adsorption isotherms were collected on a Surface Measurement Systems – DVS Advantage gravimetric sorption analyzer. N_2_ was used as the carrier gas at a flow rate of 200 sccm·min^−1^ and the temperature was kept constant at 298 K. Data were collected in 5% steps of relative humidity (RH) with a stop criterion per step of 0.01 dm/dt (wt%·min^−1^) for 5 min and a minimum time per step of 10 min.

### DFT Simulations

DFT calculations were conducted within the plane‐wave formalism by exploiting the version 6.2.0 of the VASP code.^[^ ^72^, ^73^ ^]^ The PBE exchange‐correlation functional^[^ ^74^ ^]^ was employed, using the DFT‐D3^[^ ^75^ ^]^ dispersion scheme with Becke‐Johnson damping^[^ ^76^ ^]^ to consider dispersion forces. Core electrons were treated according to the PAW formalism,^[^ ^77^ ^]^ while the energy cutoff was set to 500 eV. The electronic minimization was carried out with a slight Gaussian smearing (*σ* = 0.01 eV), setting the convergence threshold to 10^−7^ eV. Geometries were optimized with the conjugate‐gradient algorithm until the residual force on each atom was below 0.005 eV Å^−1^. This computational setup recently proved its effectiveness in providing good geometric and energetic parameters in the context of flexible MOFs, with a balanced accuracy versus computational cost.^[^ ^78^, ^79^ ^]^

The minimum energy structures of the as‐synthesized and evacuated phases of Al‐TFS (optimized output structures are reported in Section S3, Supporting Information) were obtained by simultaneously optimizing the atomic coordinates and the cell shape and volume, adopting the experimental structures from PXRD as an initial structural guess. Notably, the minimization leads to minimal modifications of the cell parameters and atomic coordinates also for the evacuated phase (see Figure S37, Supporting Information), testifying to the reliability of the structure determination by FOX, even though Rietveld refinement was not performed. Harmonic vibrational frequencies and corresponding IR intensities were computed on the minimum structures by employing the DFPT approach,^[^ ^80^, ^81^ ^]^ reporting no imaginary frequencies greater than 2 cm^−1^. The final simulated IR spectra were computed by performing a convolution of the discrete spectra with a Gaussian broadening function, whose width was arbitrarily chosen to maximize the agreement with the experimental spectra. Finally, the simulated spectra were shifted with a scaling factor of 1.025, in agreement with existing benchmark studies.^[^ ^82^, ^83^ ^]^

The computed vibrational frequencies (excluding values below 100 cm^−1^) were post‐treated through the laws of statistical thermodynamics to extract the vibrational contribution to the free energy (G_vib_). The Δ*G* of adsorption of H_2_O on Al‐TFS at 300 K was computed according to the equation:

(1) $$\begin{array}{c} \Delta \;G_{\mathrm{ads}}=\frac{\left(E^{as}+G_{vib}^{as}\right)-\left[E^{ev}+G_{vib}^{ev}+3\left(E^{H_{2}O}+G_{vib}^{H_{2}O}+G_{rot}^{H_{2}O}+G_{tr}^{H_{2}O}\right)\right]}{3}\;\;\; \end{array}$$

where the apexes “*ev*” and “*as*” refer to the evacuated and as‐synthesized phase, respectively.

## Supporting Information

PXRD patterns, FE‐SEM images, TGA curve, NMR spectra, Rietveld plots and Pawley refinements, crystal structures, IR and SSNMR spectra, DFT optimized structures, physisorption curves, BET and DFT fit, heat of adsorption and CO_2_/N_2_ – CO_2_/CH_4_ selectivity (PDF); volumetric isotherms (aif), crystallographic data (cif).

## Author Contributions

M.S.N., D.M.V., and M.C. performed Al‐TFS synthesis and its basic characterization (PXRD, TGA, ICP‐OES, FE‐SEM). M.S.N. and M.S. acquired the PXRD patterns for structure solution and refinement. M.T., D.M.V., and F.C. performed ab initio structure solution and Rietveld refinement from PXRD data. M.T. performed solution NMR data collection and analysis. L.C. and F.N. carried out the SSNMR experiments and data analysis. V.G. and A.R. performed the time‐resolved DRIFT experiments. A.R. and M.S. carried out the DFT simulations. V.G. and V.C. carried out the IR experiments and data analysis. V.G. collected volumetric adsorption isotherms of N_2_, Ar and CO_2_. D.M.V. and C.M. collected the gravimetric water adsorption isotherms. V.G. and V.C. carried out the data analysis of all adsorption isotherms. V.G. and M.S. employed *Zeo++* software for textural properties evaluation. V.G. wrote the manuscript with contributions from L.C., M.T., F.C., and V.C. M.T., F.C., and V.C. coordinated and designed the research.

## Accession Codes

CCDC 2 280 453 (as‐synthesized) contains the crystallographic data of Al‐TFS.

## Conflict of Interests

The authors declare that they have no known competing financial interests or personal relationships that could have appeared to influence the work reported in this paper.

## Supporting information



Supporting Information

Supporting Information

## Data Availability

The data that support the findings of this study are available from the corresponding author upon reasonable request. DFT structures have been made available in a Materials Cloud record with the identifier: https://doi.org/10.24435/materialscloud:d6‐dy. All adsorption isotherms are available as supporting material in the AIF format.

## References

[1] A. U. Czaja , N. Trukhan , U. Müller , Chem. Soc. Rev. 2009, 38, 1284. 19384438 10.1039/b804680h

[2] P. Silva , S. M. F. Vilela , J. P. C. Tomé , F. A. Almeida Paz , Chem. Soc. Rev. 2015, 44, 6774. 26161830 10.1039/c5cs00307e

[3] D. Bazer‐Bachi , L. Assié , V. Lecocq , B. Harbuzaru , V. Falk , Powder Technol. 2014, 255, 52.

[4] J. R. Li , J. Sculley , H. C. Zhou , Chem. Rev. 2012, 112, 869. 21978134 10.1021/cr200190s

[5] G. Férey , C. Serre , T. Devic , G. Maurin , H. Jobic , P. L. Llewellyn , G. De Weireld , A. Vimont , M. Daturi , J.‐S. Chang , Chem. Soc. Rev. 2011, 40, 550. 21180728 10.1039/c0cs00040j

[6] S. Choi , J. H. Drese , C. W. Jones , ChemSusChem 2009, 2, 796. 19731282 10.1002/cssc.200900036

[7] V. I. Isaeva , L. M. Kustov , Pet. Chem. 2010, 50, 167.

[8] D. Yang , B. C. Gates , ACS Catal. 2019, 9, 1779.

[9] P. Horcajada , C. Serre , M. Vallet‐Regí , M. Sebban , F. Taulelle , G. Férey , Angew. Chem., Int. Ed. 2006, 45, 5974. 10.1002/anie.200601878 16897793

[10] P. Horcajada , R. Gref , T. Baati , P. K. Allan , G. Maurin , P. Couvreur , G. Férey , R. E. Morris , C. Serre , Chem. Rev. 2012, 112, 1232. 22168547 10.1021/cr200256v

[11] P. A. Julien , C. Mottillo , T. Friščić , Green Chem. 2017, 19, 2729.

[12] S. Wang , C. Serre , ACS Sustain. Chem. Eng. 2019, 7, 11911.

[13] M. Rubio‐Martinez , C. Avci‐Camur , A. W. Thornton , I. Imaz , D. Maspoch , M. R. Hill , Chem. Soc. Rev. 2017, 46, 3453. 28530737 10.1039/c7cs00109f

[14] T. Loiseau , C. Serre , C. Huguenard , G. Fink , F. Taulelle , M. Henry , T. Bataille , G. Férey , Chem. – A Eur. J. 2004, 10, 1373. 10.1002/chem.200305413 15034882

[15] M. Latroche , S. Surblé , C. Serre , C. Mellot‐Draznieks , P. L. Llewellyn , J.‐H. Lee , J.‐S. Chang , S. H. Jhung , G. Férey , Angew. Chem., Int. Ed. 2006, 45, 8227. 10.1002/anie.200600105 17121399

[16] C. Volkringer , D. Popov , T. Loiseau , N. Guillou , G. Ferey , M. Haouas , F. Taulelle , C. Mellot‐Draznieks , M. Burghammer , C. Riekel , Nat. Mater. 2007, 6, 760. 17873864 10.1038/nmat1991

[17] A. Boutin , F.‐X. Coudert , M.‐A. Springuel‐Huet , A. V. Neimark , G. Férey , A. H. Fuchs , J. Phys. Chem. C. 2010, 114, 22237.

[18] W. P. Mounfield , K. S. Walton , J. Colloid Interface Sci. 2015, 447, 33. 25697686 10.1016/j.jcis.2015.01.027

[19] P. Mishra , H. P. Uppara , B. Mandal , S. Gumma , Ind. Eng. Chem. Res. 2014, 53, 19747.

[20] L. Hamon , C. Serre , T. Devic , T. Loiseau , F. Millange , G. Férey , G. De Weireld , J. Am. Chem. Soc. 2009, 131, 8775. 19505146 10.1021/ja901587t

[21] F. Jeremias , A. Khutia , S. K. Henninger , C. Janiak , J. Mater. Chem. 2012, 22, 10148.

[22] T. Steenhaut , Y. Filinchuk , S. Hermans , J. Mater. Chem. A 2021, 9, 21483.

[23] T. Steenhaut , L. Fusaro , K. Robeyns , S. Lacour , X. Li , J. G. Mahy , V. Louppe , N. Grégoire , G. Barozzino‐Consiglio , J.‐F. Statsyns , C. Aprile , Y. Filinchuk , S. Hermans , Inorg. Chem. 2021, 60, 16666. 34652917 10.1021/acs.inorgchem.1c02568

[24] R. Rojas‐Luna , J. Amaro‐Gahete , D. G. Gil‐Gavilán , M. Castillo‐Rodríguez , C. Jiménez‐Sanchidrián , J. R. Ruiz , D. Esquivel , F. J. Romero‐Salguero , Microporous Mesoporous Mater. 2023, 355, 112565.

[25] E. Leung , U. Muller , N. Trukhan , H. Mattenheimer , G. Cox , S. Blei , Process for Preparing Porous Metal–Organic Frameworks Based on Aluminum Fumarate. 2013, 8524932.

[26] E. Alvarez , N. Guillou , C. Martineau , B. Bueken , B. Van de Voorde , C. Le Guillouzer , P. Fabry , F. Nouar , F. Taulelle , D. de Vos , J.‐S. Chang , K. H. Cho , N. Ramsahye , T. Devic , M. Daturi , G. Maurin , C. Serre , Angew. Chem., Int. Ed. 2015, 54, 3664. 10.1002/anie.201410459 25655768

[27] M. Gaab , N. Trukhan , S. Maurer , R. Gummaraju , U. Müller , Microporous Mesoporous Mater. 2012, 157, 131.

[28] Z. Huang , P. Hu , J. Liu , F. Shen , Y. Zhang , K. Chai , Y. Ying , C. Kang , Z. Zhang , H. Ji , Sep. Purif. Technol. 2022, 286, 120446.

[29] Q. Liu , Y. Ding , Q. Liao , X. Zhu , H. Wang , J. Yang , Colloids Surf A Physicochem Eng Asp. 2019, 579, 123645.

[30] J. A. Coelho , A. M. Ribeiro , A. F. P. Ferreira , S. M. P. Lucena , A. E. Rodrigues , D. C. S. de Azevedo , Ind. Eng. Chem. Res. 2016, 55, 2134.

[31] E. Elsayed , R. AL‐Dadah , S. Mahmoud , P. Anderson , A. Elsayed , Desalination. 2020, 475, 114170.

[32] E. Moumen , L. Bazzi , S. El Hankari , Process Saf. Environ. Prot. 2022, 160, 502.

[33] A. Nuhnen , C. Janiak , in New Trends in Macromolecular and Supramolecular Chemistry for Biological Applications (Eds.: M. J.M. Abadie , M. Pinteala , A. Rotaru ), Springer International Publishing, Cham, pp. 87.

[34] F. Jeremias , D. Fröhlich , C. Janiak , S. K. Henninger , RSC Adv. 2014, 4, 24073.

[35] G. Férey , Dalton Trans. 2016, 45, 4073. 26537002 10.1039/c5dt03547c

[36] A. Ebadi Amooghin , H. Sanaeepur , R. Luque , H. Garcia , B. Chen , Chem. Soc. Rev. 2022, 51, 7427. 35920324 10.1039/d2cs00442a

[37] J. Zheng , R. S. Vemuri , L. Estevez , P. K. Koech , T. Varga , D. M. Camaioni , T. A. Blake , B. P. McGrail , R. K. Motkuri , J. Am. Chem. Soc. 2017, 139, 10601. 28702994 10.1021/jacs.7b04872

[38] A. Cadiau , Y. Belmabkhout , K. Adil , P. M. Bhatt , R. S. Pillai , A. Shkurenko , C. Martineau‐Corcos , G. Maurin , M. Eddaoudi , Science 2017, 356, 731. 28522529 10.1126/science.aam8310

[39] M. Cavallo , C. Atzori , M. Signorile , F. Costantino , D. Morelli Venturi , A. Koutsianos , K. A. Lomachenko , L. Calucci , F. Martini , A. Giovanelli , M. Geppi , V. Crocellà , M. Taddei , J. Mater. Chem. A. 2023, 11, 5568. 10.1039/d2ta09746j PMC10012411 36936468

[40] D. Morelli Venturi , F. Costantino , RSC Adv. 2023, 13, 29215. 37809027 10.1039/d3ra04940j PMC10551664

[41] R. D'Amato , A. Donnadio , M. Carta , C. Sangregorio , D. Tiana , R. Vivani , M. Taddei , F. Costantino , ACS Sustain. Chem. Eng. 2019, 7, 394.

[42] D. Morelli Venturi , M. S. Notari , R. Bondi , E. Mosconi , W. Kaiser , G. Mercuri , G. Giambastiani , A. Rossin , M. Taddei , F. Costantino , ACS Appl. Mater. Interfaces 2022, 14, 40801. 36039930 10.1021/acsami.2c07640 PMC9478941

[43] D. Morelli Venturi , V. Guiotto , R. D'Amato , L. Calucci , M. Signorile , M. Taddei , V. Crocellà , F. Costantino , Mol. Syst. Des. Eng. 2023, 8, 586.

[44] D. Znidar , D. Dallinger , C. O. Kappe , ACS Chem. Health Safety. 2022, 29, 165.

[45] K.‐W. Jung , B. H. Choi , S. Y. Lee , K.‐H. Ahn , Y. J. Lee , J. Industrial Eng. Chem. 2018, 67, 316.

[46] M. R. Mani , R. Chellaswamy , Y. N. Marathe , V. K. Pillai , RSC Adv. 2016, 6, 1907.

[47] V. Favre‐Nicolin , R. Černý , Powder Diffr. 2005, 20, 359.

[48] A. A. Coelho , Coelho Software, Brisbane, Australia. 2007;

[49] K. I. Hadjiivanov , D. A. Panayotov , M. Y. Mihaylov , E. Z. Ivanova , K. K. Chakarova , S. M. Andonova , N. L. Drenchev , Chem. Rev. 2021, 121, 1286. 33315388 10.1021/acs.chemrev.0c00487

[50] K. Charles , in Introduction to Solid State Physics, John Wiley & Sons, NewYork, NY 2024.

[51] M. Haouas , F. Taulelle , C. Martineau , Prog. Nucl. Magn. Reson. Spectrosc. 2016, 94–95, 11. 10.1016/j.pnmrs.2016.01.003 27247283

[52] C. Lieder , S. Opelt , M. Dyballa , H. Henning , E. Klemm , M. Hunger , J. Phys. Chem. C 2010, 114, 16596.

[53] Y. Zhang , B. E. G. Lucier , S. M. McKenzie , M. Arhangelskis , A. J. Morris , T. Friščić , J. W. Reid , V. V. Terskikh , M. Chen , Y. Huang , ACS Appl. Mater. Interfaces 2018, 10, 28582. 30070824 10.1021/acsami.8b08562

[54] J. W. M. Osterrieth , J. Rampersad , D. Madden , N. Rampal , L. Skoric , B. Connolly , M. D. Allendorf , V. Stavila , J. L. Snider , R. Ameloot , J. Marreiros , C. Ania , D. Azevedo , E. Vilarrasa‐Garcia , B. F. Santos , X. H. Bu , Z. Chang , H. Bunzen , N. R. Champness , S. L. Griffin , B. Chen , R. B. Lin , B. Coasne , S. Cohen , J. C. Moreton , Y. J. Colón , L. Chen , R. Clowes , F. X. Coudert , Y. Cui , et al., Adv. Mater. 2022, 34, 2201502. 10.1002/adma.202201502 35603497

[55] M. Thommes , K. Kaneko , A. V. Neimark , J. P. Olivier , F. Rodriguez‐Reinoso , J. Rouquerol , K. S. W. Sing , Pure Appl. Chem. 2015, 87, 1051.

[56] A. Datar , S. Yoon , L. C. Lin , Y. G. Chung , Langmuir. 2022, 38, 11631. 36095324 10.1021/acs.langmuir.2c01390

[57] B. Bozbiyik , J. Lannoeye , D. E. De Vos , G. V. Baron , J. F. M. Denayer , Phys. Chem. Chem. Phys. 2016, 18, 3294. 26752453 10.1039/c5cp06342f

[58] T. J. Matemb Ma Ntep , W. Wu , H. Breitzke , C. Schlüsener , B. Moll , L. Schmolke , G. Buntkowsky , C. Janiak , Aust. J. Chem. 2019, 72, 835. 10.1021/acs.inorgchem.9b01408 31364846

[59] M. L. Foo , R. Matsuda , Y. Hijikata , R. Krishna , H. Sato , S. Horike , A. Hori , J. Duan , Y. Sato , Y. Kubota , M. Takata , S. Kitagawa , J. Am. Chem. Soc. 2016, 138, 3022. 26876504 10.1021/jacs.5b10491

[60] J. A. Mason , J. Oktawiec , M. K. Taylor , M. R. Hudson , J. Rodriguez , J. E. Bachman , M. I. Gonzalez , A. Cervellino , A. Guagliardi , C. M. Brown , P. L. Llewellyn , N. Masciocchi , J. R. Long , Nature 2015, 527, 357. 26503057 10.1038/nature15732

[61] Z. Li , K. Shi , L. Zhai , Z. Wang , H. Wang , Y. Zhao , J. Wang , Sep. Purif. Technol. 2023, 307, 122725.

[62] B. Bozbiyik , T. Van Assche , J. Lannoeye , D. E. De Vos , G. V. Baron , J. F. M. Denayer , Adsorption. 2017, 23, 185.

[63] D. W. Siderius , V. K. Shen , R. Johnson, III , R. D. van Zee , NIST/ARPA‐E Database of Novel and Emerging Adsorbent Materials, NIST Standard Reference Database Number 205, n.d http://Adsorbents.Nist.Gov.

[64] Y. Moroi , H. Yano , O. Shibata , T. Yonemitsu , Bull. Chem. Soc. Jpn. 2001, 74, 667.

[65] M. R. Popović‐Nikolić , G. V. Popović , K. Stojilković , M. Dobrosavljević , D. D. Agbaba , J. Chem. Eng. Data. 2018, 63, 3150.

[66] P.‐E. Werner , L. Eriksson , M. Westdahl , J. Appl. Crystallogr. 1985, 18, 367.

[67] R. K. Harris , E. D. Becker , S. M. Cabral de Menezes , R. Goodfellow , P. Granger , Solid State Nucl. Magn. Reson. 2002, 22, 458. 12637147 10.1006/snmr.2002.0063

[68] J. Rouquerol , P. Llewellyn , F. Rouquerol , Stud. Surf. Sci. Catal. 2007, 160, 49.

[69] V. Crocellà , C. Atzori , G. Latini , M. Signorile , A Kit for Volumetric Measurements of Gas Adsorption. 2021, WO Pat. 2021181211A1.

[70] T. F. Willems , C. H. Rycroft , M. Kazi , J. C. Meza , M. Haranczyk , Microporous Mesoporous Mater. 2012, 149, 134.

[71] S. Lee , J. Lee , J. Kim , Korean J. Chem. Eng. 2017, 35, 214.

[72] G. Kresse , J. Hafner , Phys. Rev. B. 1994, 49, 14251. 10.1103/physrevb.49.14251 10010505

[73] G. Kresse , J. Furthmüller , Comput. Mater. Sci. 1996, 6, 15.

[74] J. P. Perdew , K. Burke , M. Ernzerhof , Phys. Rev. Lett. 1996, 77, 3865. 10062328 10.1103/PhysRevLett.77.3865

[75] S. Grimme , J. Antony , S. Ehrlich , H. Krieg , J. Chem. Phys. 2010, 132, 154104. 20423165 10.1063/1.3382344

[76] S. Grimme , S. Ehrlich , L. Goerigk , J. Comput. Chem. 2011, 32, 1456. 21370243 10.1002/jcc.21759

[77] G. Kresse , D. Joubert , Phys. Rev. B. 1999, 59, 1758.

[78] J. Wieme , K. Lejaeghere , G. Kresse , V. Van Speybroeck , Nat. Commun. 2018, 9, 4899. 30464249 10.1038/s41467-018-07298-4 PMC6249296

[79] A. E. J. Hoffman , L. Vanduyfhuys , I. Nevjestić , J. Wieme , S. M. J. Rogge , H. Depauw , P. Van Der Voort , H. Vrielinck , V. Van Speybroeck , J. Phys. Chem. C. 2018, 122, 2734. 10.1021/acs.jpcc.7b11031 PMC5808359 29449906

[80] S. Baroni , S. de Gironcoli , A. Dal Corso , P. Giannozzi , Rev. Mod. Phys. 2001, 73, 515.

[81] P. Giannozzi , S. Baroni , J. Chem. Phys. 1994, 100, 8537.

[82] J. P. Merrick , D. Moran , L. Radom , J. Phys. Chem. A 2007, 111, 11683. 17948971 10.1021/jp073974n

[83] I. M. Alecu , J. Zheng , Y. Zhao , D. G. Truhlar , J. Chem. Theory Comput. 2010, 6, 2872. 26616087 10.1021/ct100326h

