# Supplementary material for: A Flexible, Perfluorinated Analog of Aluminum Fumarate Metal–Organic Framework
Source: Chemistry. 2025 May 29;31(35):e202500130. doi: 10.1002/chem.202500130 (PMC12188177; doi:10.1002/chem.202500130)
Supplement: Supplementary file 1 — Supporting Information [file CHEM-31-e202500130-s001.docx]

**A Flexible, Perfluorinated Analog of Aluminum Fumarate Metal-Organic Framework**

Virginia Guiotto^[a]^, Maria Sole Notari^[b]^, Diletta Morelli Venturi^[c,d]^, Alberto Ricchebuono^[a]^, Melissa Castagnoli^[b]^, Christoph Meier^[c]^, Francesca Nardelli^[e]^, Lucia Calucci^[e,f]^, Matteo Signorile^[a]^, Marco Taddei^[f,g]^*, Valentina Crocellà^[a]^*, Ferdinando Costantino^[b]^*

[a] V. Guiotto, Dr. A. Ricchebuono, Dr. M. Signorile, Prof. V. Crocellà

Dipartimento di Chimica, Centro di Riferimento NIS,

Unità di Ricerca INSTM, Università degli Studi di Torino,

Via G. Quarello 15/A and Via P. Giuria 7, I-10125 Torino, Italy.

E-mail: [valentina.crocella@unito.it](mailto:valentina.crocella@unito.it)

[b] M.S. Notari, M. Castagnoli, Prof. F. Costantino

Dipartimento di Chimica, Biologia e Biotecnologie,

Unità di Ricerca INSTM, Università di Perugia,

Via Elce di Sotto 8, 06123 Perugia, Italy.

E-mail: [ferdinando.costantino@unipg.it](mailto:ferdinando.costantino@unipg.it)

[c] C. Meier, Dr. D. Morelli Venturi

Institute of Inorganic Chemistry,

Christian-Albrechts University of Kiel

Max-Eyth Straße 2, Kiel, Germany

[d] Dr. D. Morelli Venturi

Kiel Nano, Surface and Interface Science KiNSIS,

Christian-Albrecht University of Kiel,

Christian-Albrechts-Platz 4, 24118 Kiel, Germany

[e] Dr. F. Nardelli, Dr. L. Calucci

Istituto di Chimica dei Composti Organo Metallici,

Unità di Ricerca INSTM, Consiglio Nazionale delle Ricerche,

Via Giuseppe Moruzzi 1, 56124 Pisa, Italy

[f] Dr. L. Calucci, Prof. M. Taddei

Centro per l’Integrazione della Strumentazione Scientifica dell’Università di Pisa (CISUP),

Lungarno Pacinotti 43/44, 56126 Pisa, Italy

[g] Prof. M. Taddei

Dipartimento di Chimica e Chimica Industriale,

Unità di Ricerca INSTM, Università di Pisa,

Via Giuseppe Moruzzi 13, 56124 Pisa, Italy.

E-mail: [marco.taddei@unipi.it](mailto:marco.taddei@unipi.it)

**Contents**

1. Synthesis and basic characterization 3

2. Crystal structure 8

3. *In situ* IR spectroscopy 16

4. SSNMR spectroscopy 20

5. Evaluation of the textural properties 23

6. Evaluation of Al-TFS CO_2_ sorption performances 26

7. H_2_O sorption properties of Al-TFS 30

1. Synthesis and basic characterization


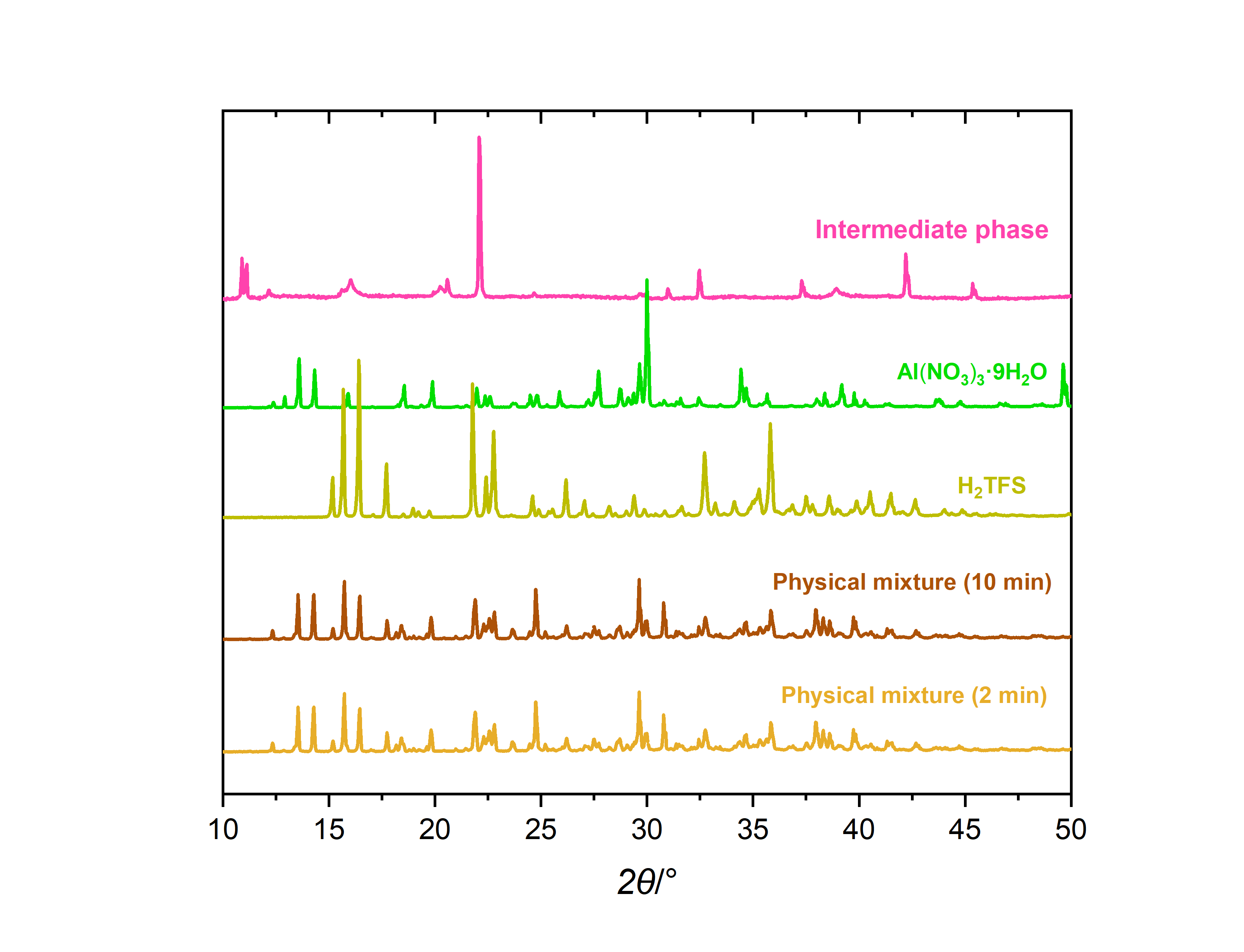


**Figure S1** PXRD patterns of the physical mixture of the reagents ground for two minutes (orange) and ten minutes (light brown), of pure H_2_TFS ligand (dark yellow), of pure Al(NO_3_)_3_·9H_2_O salt (bright green) and of the “intermediate phase” after 24 h in oven at 393 K and before the workup (magenta).


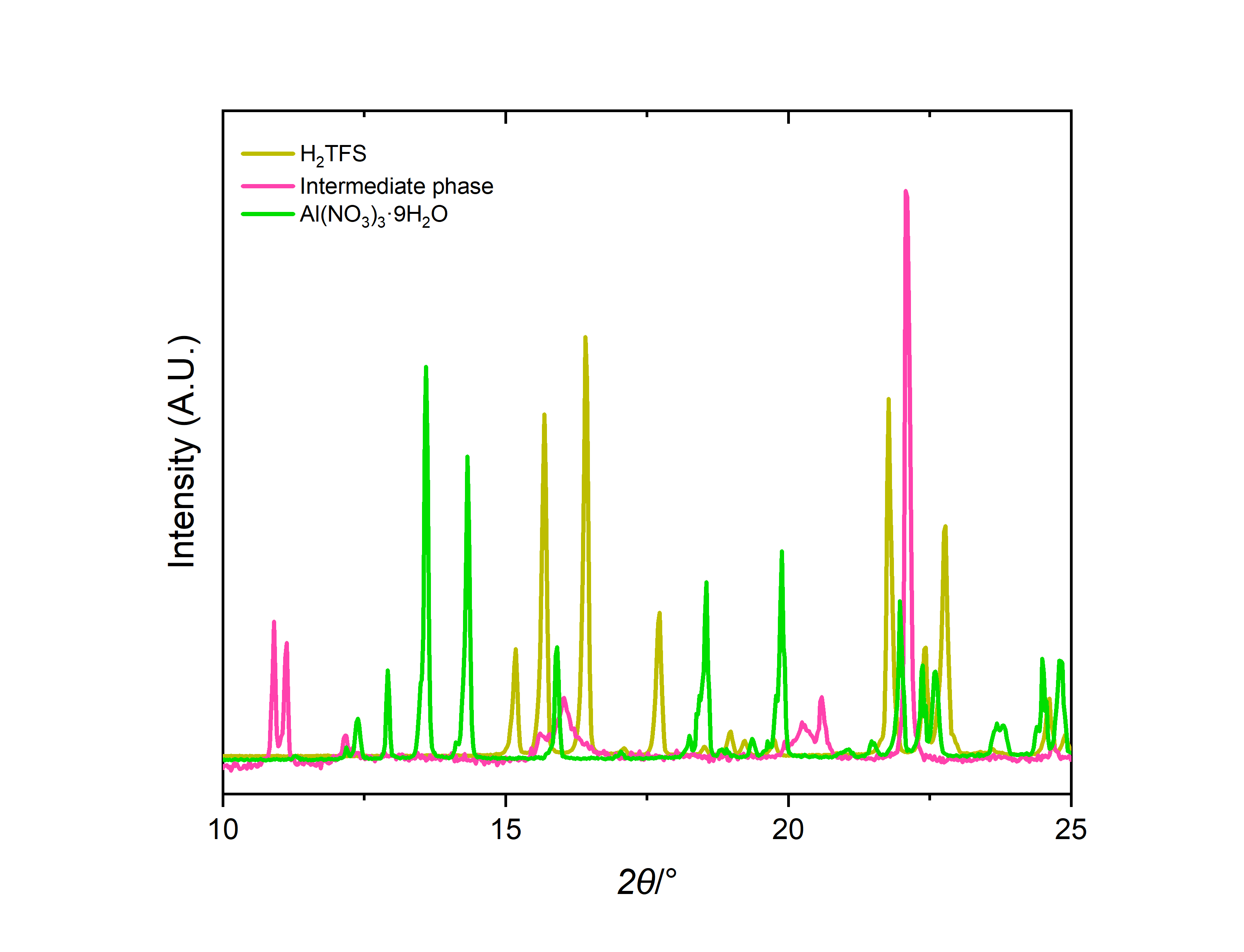


**Figure S2** Comparison between the PXRD patterns of the pure reagents (dark yellow and bright green) and the “intermediate phase” after heating and before workup (magenta).


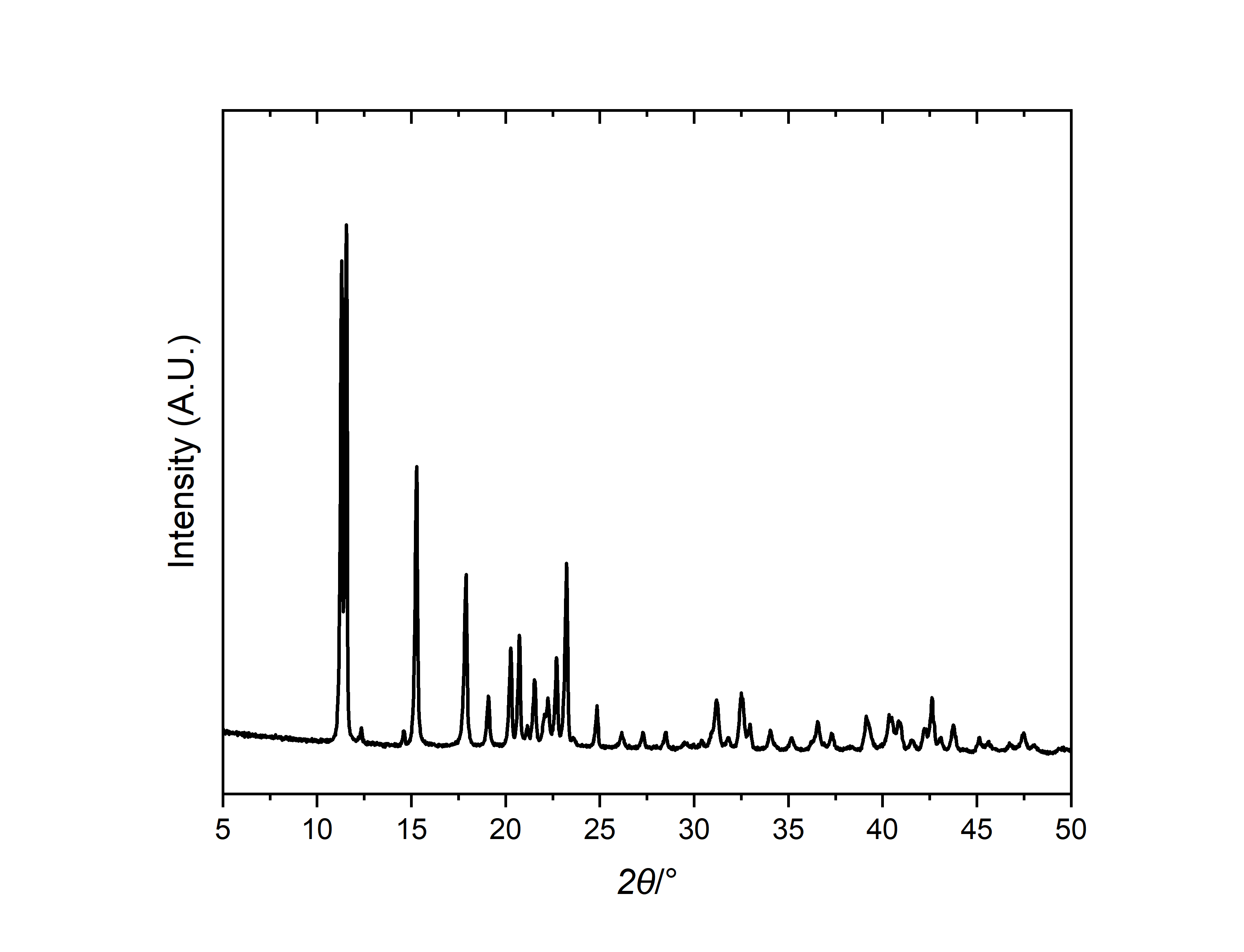


**Figure S3** PXRD pattern of as-synthesized Al-TFS.







**Figure S4** FE-SEM images of as-synthesized Al-TFS.


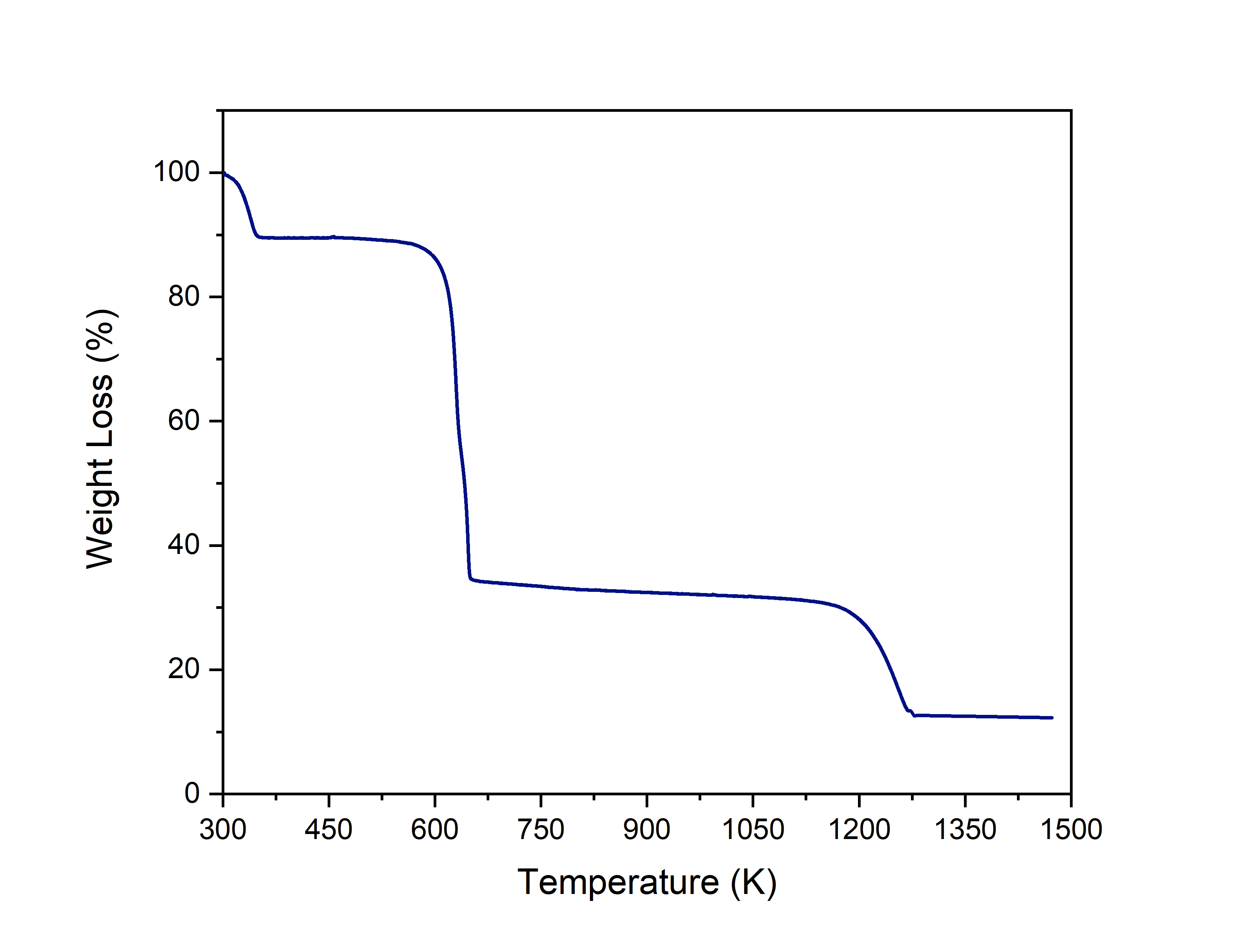


**Figure S5** Thermogravimetric curve of as-synthesized Al-TFS.





**Figure S6** PXRD pattern of Al-TFS calcined at 873 K for 4 hours (green), compared with the calculated patterns for Al_2_(OH)_3_F_3_ (red) and α-Al_2_O_3_ (black).


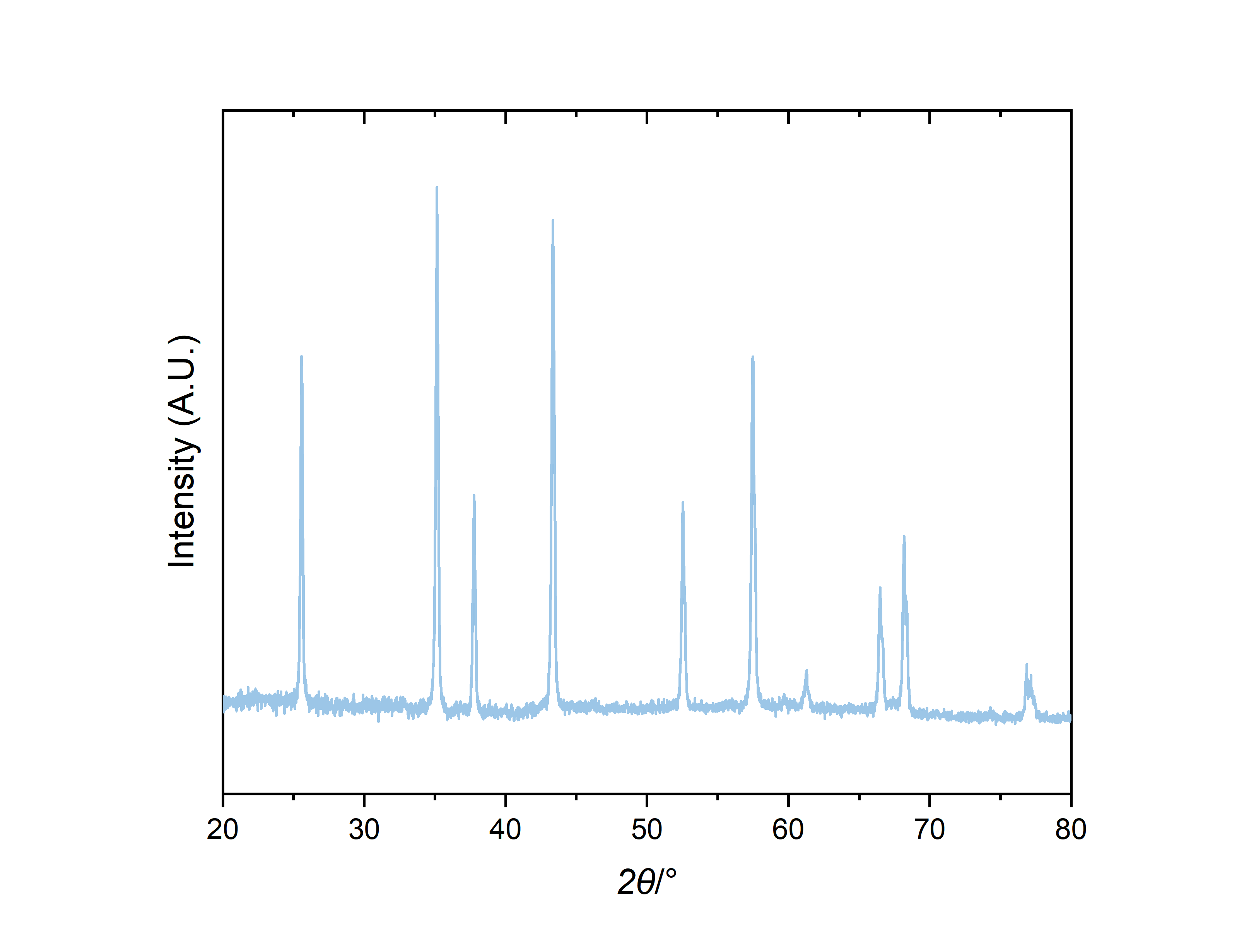


**Figure S7** PXRD pattern of residual α-Al_2_O_3_ after the TGA analysis of Al-TFS (sample heated up to T = 1473 K)


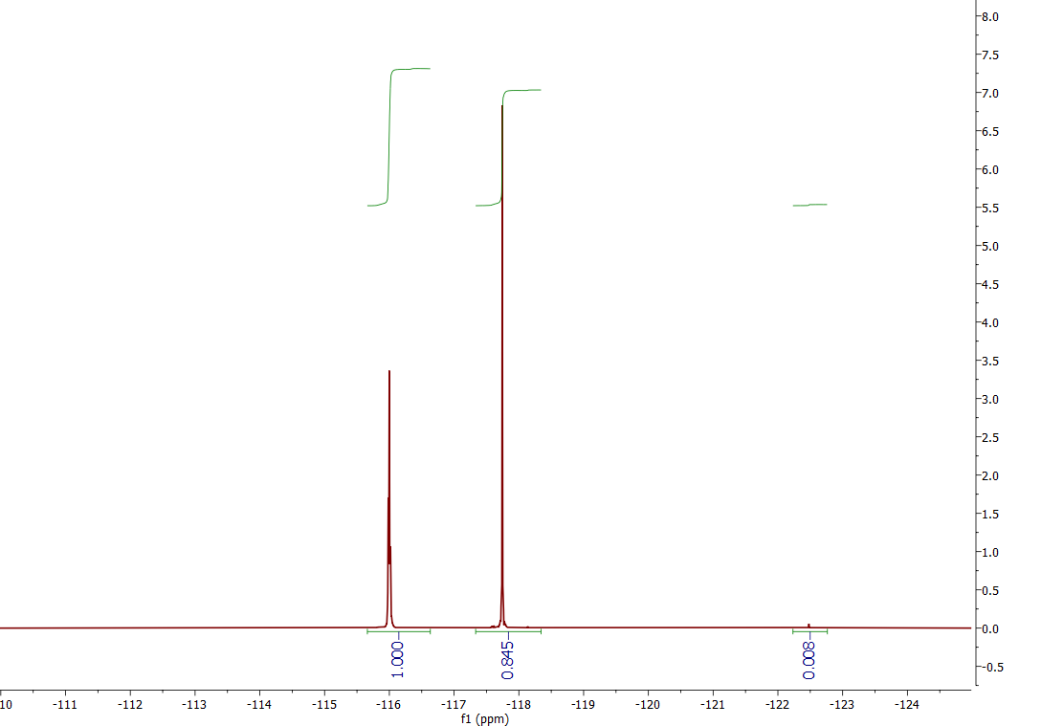


**Figure S8** Solution ^19^F NMR spectrum showing signals of TFS^2-^ (-117.8 ppm) and 2,6-difluorobenzoic acid (-116.0 ppm) recorded on 10.1 mg of evacuated Al-TFS digested in 1 mL of 1 M NaOH in D_2_O in the presence of 0.100 M 2,6-difluorobenzoic acid as standard. The signal of TFS^2-^ is found at –117.8 ppm and accounts for four fluorides, while the signal of 2,6-difluorobenzoic acid is found at –116.0 ppm and accounts for two fluorides. The negligible signal at –122.5 ppm is probably arising from fluoride derived from linker decomposition during synthesis. By dividing the signal of TFS^2-^ by two, a normalised integral of 0.423 is obtained. The concentration of TFS^2-^ is derived to be 0.0423 M, corresponding to 7.94 mg of TFS^2-^ in the original solid (78.6 wt% vs 81.0 wt% calculated for the formula Al(OH)TFS).

1. Crystal structure

**Table S1.** Crystallographic data for as-synthesized Al-TFS.

| Compound | **Al-TFS** |
| --- | --- |
| CCDC number | **2280453** |
| Empirical formula | Al_2_C_8_O_13_F_8_H_8_ |
| Formula weight/g mol^−1^ | 518.04 |
| Crystal system | Triclinic |
| Space Group | *P*-1 |
| *a*/Å | 6.6310(16) |
| *b*/Å | 8. 8686(17) |
| *c*/Å | 8.9162(19) |
| *a*/° | 107.5447(16) |
| *β*/° | 109.2433(19) |
| *γ*/° | 103.979(2) |
| Volume/Å^3^ | 436.78(2) |
| *Z* | 1 |
| Calc. density/g cm^-3^ | 1.97 |
| Pattern Range, 2θ/° | 5-120 |
| N. of data | 13764 |
| N. of reflections | 4306 |
| N. of variables | 90 |
| N. of restraints | 67 |
| *R_p_* | 5.85 |
| *R_wp_* | 8.06 |
| *R_bragg_* | 1.59 |
| *GoF* | 3.14 |


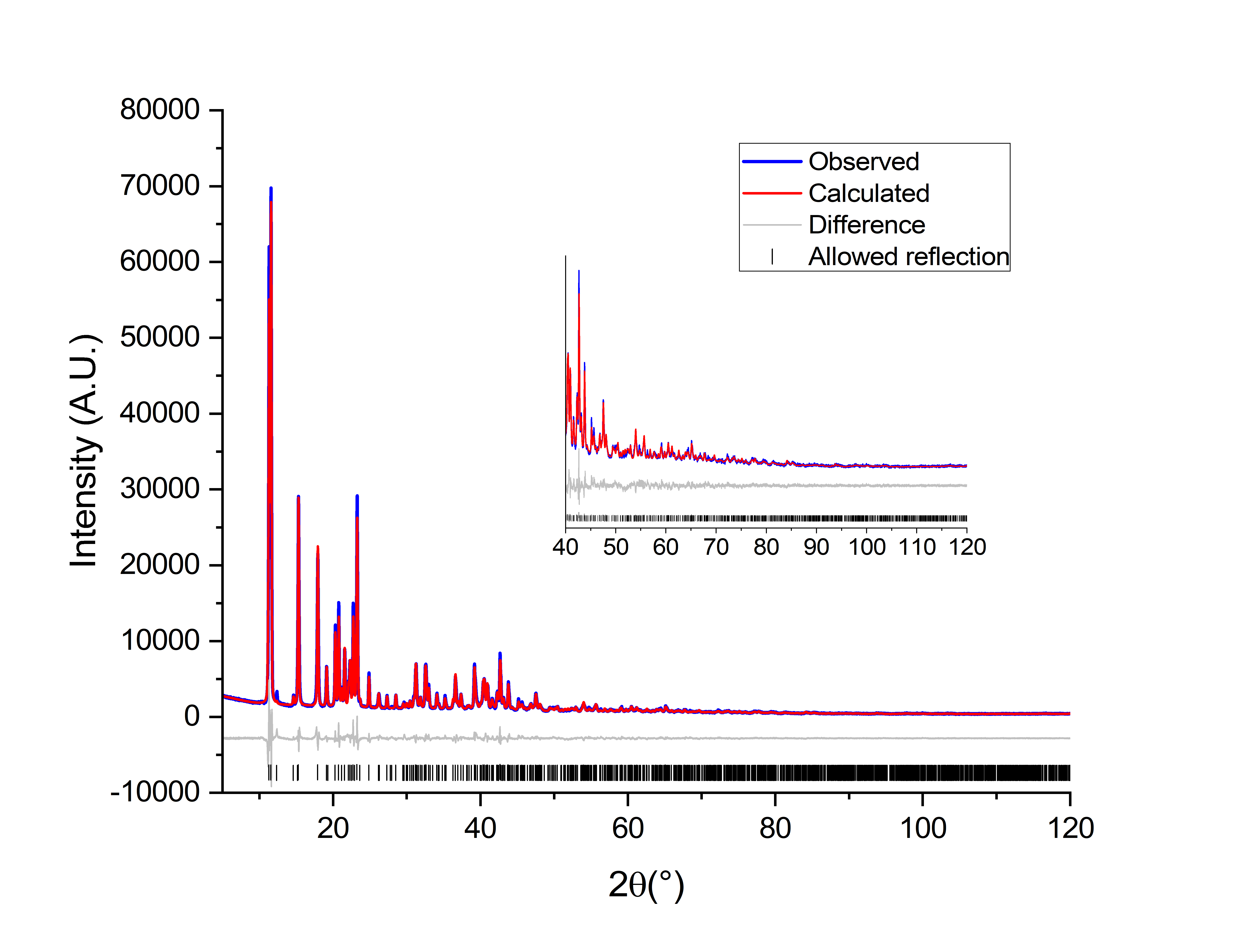


**Figure S9** Rietveld refinement carried out on the PXRD pattern (λ= 1.5401 Å) of as-synthesized Al-TFS. The experimental PXRD pattern is shown in blue, the calculated in red and the difference (experimental - calculated) is given in grey. The allowed reflection positions are given in black.


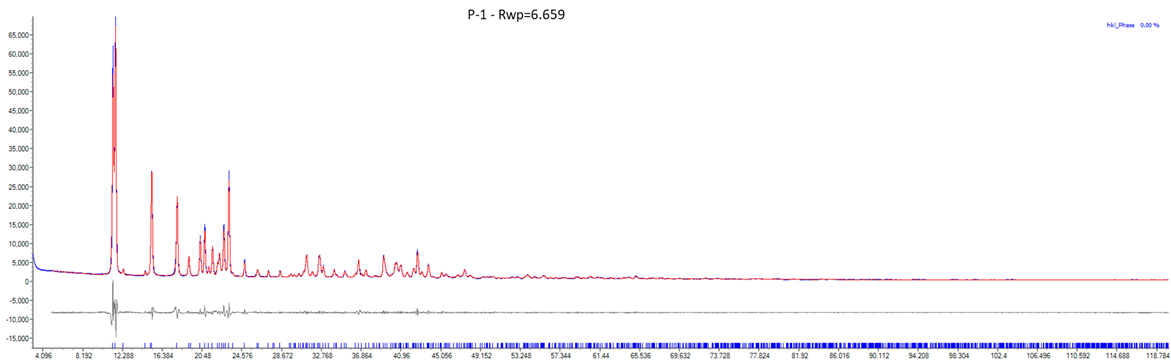


**Figure S10** Pawley refinement of the PXRD pattern of as-synthesized Al-TFS in the triclinic P-1 space group.


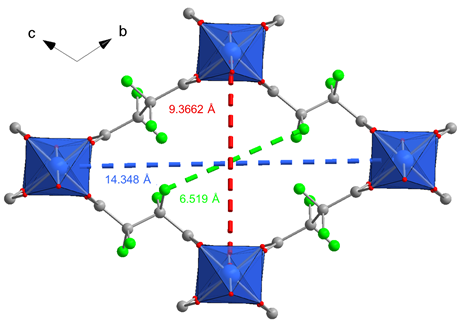


**Figure S11** The F-F (green dashed line) and Al-Al distances (red and blue dashed lines) defining the size of the channels in as-synthesized Al-TFS. Colour code: Al, blue; C, grey; O, red; F, green.


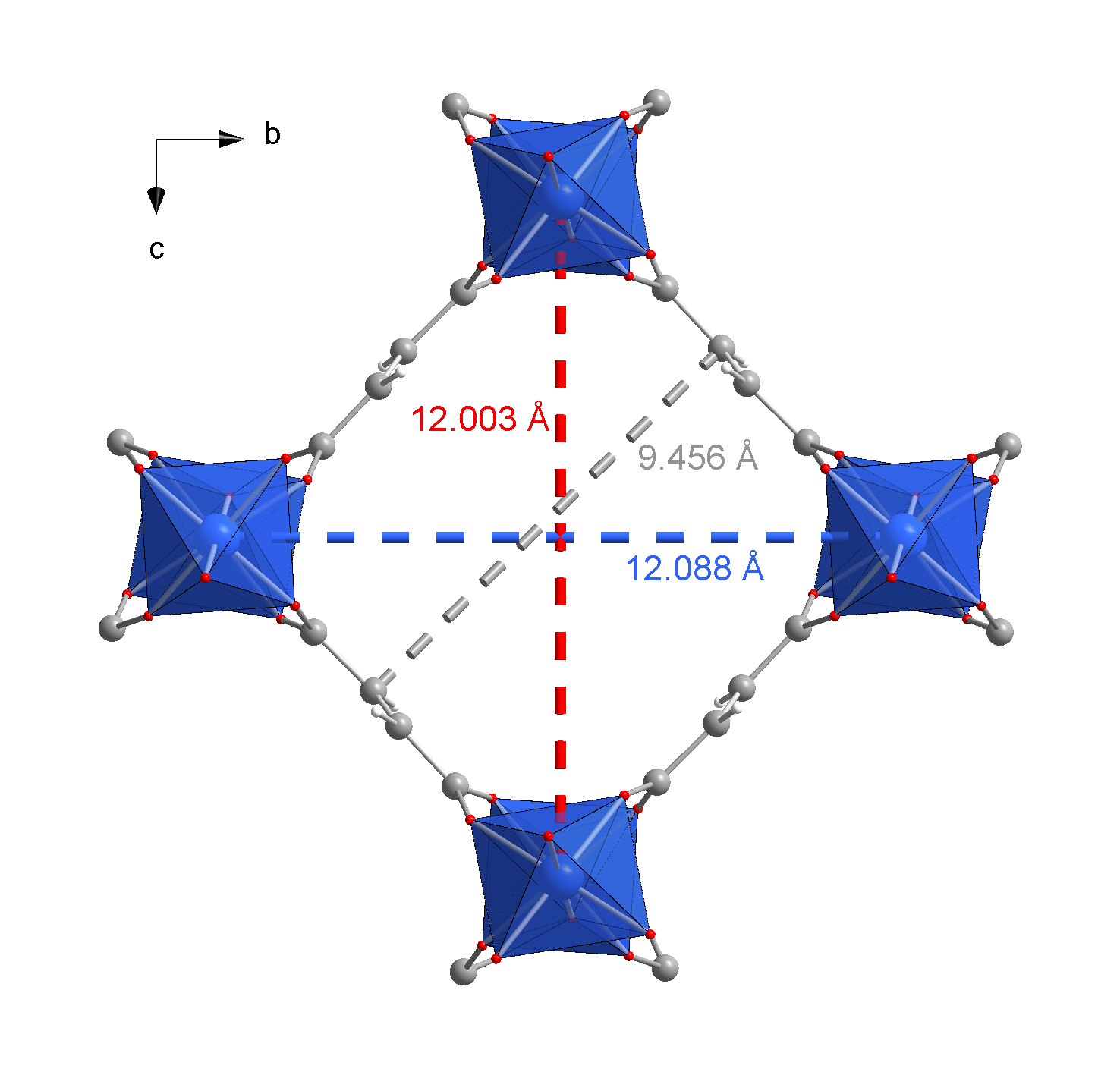


**Figure S12** The C-C (grey dashed line) and Al-Al distances (red and blue dashed lines) defining the size of the channels in Al-FUM. Colour code: Al, blue; C, grey; O, red; H, white.


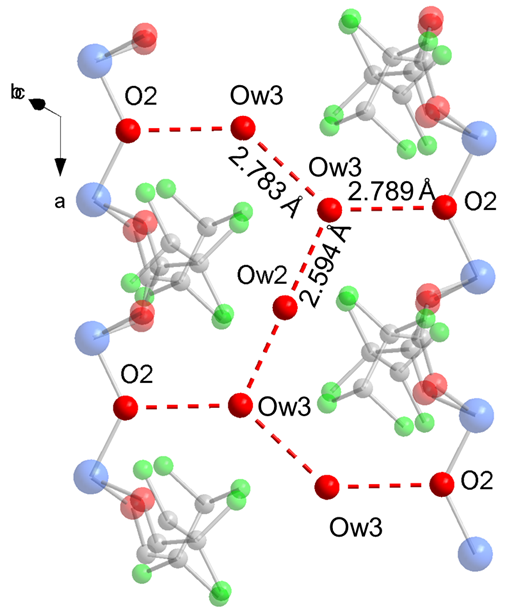


**Figure S13** The hydrogen bonding network involving Ow3, Ow2 and O2 in as-synthesized Al-TFS. Atoms not involved in the interactions are shaded. Colour code: Al, blue; C, grey; O, red; F, green.

**
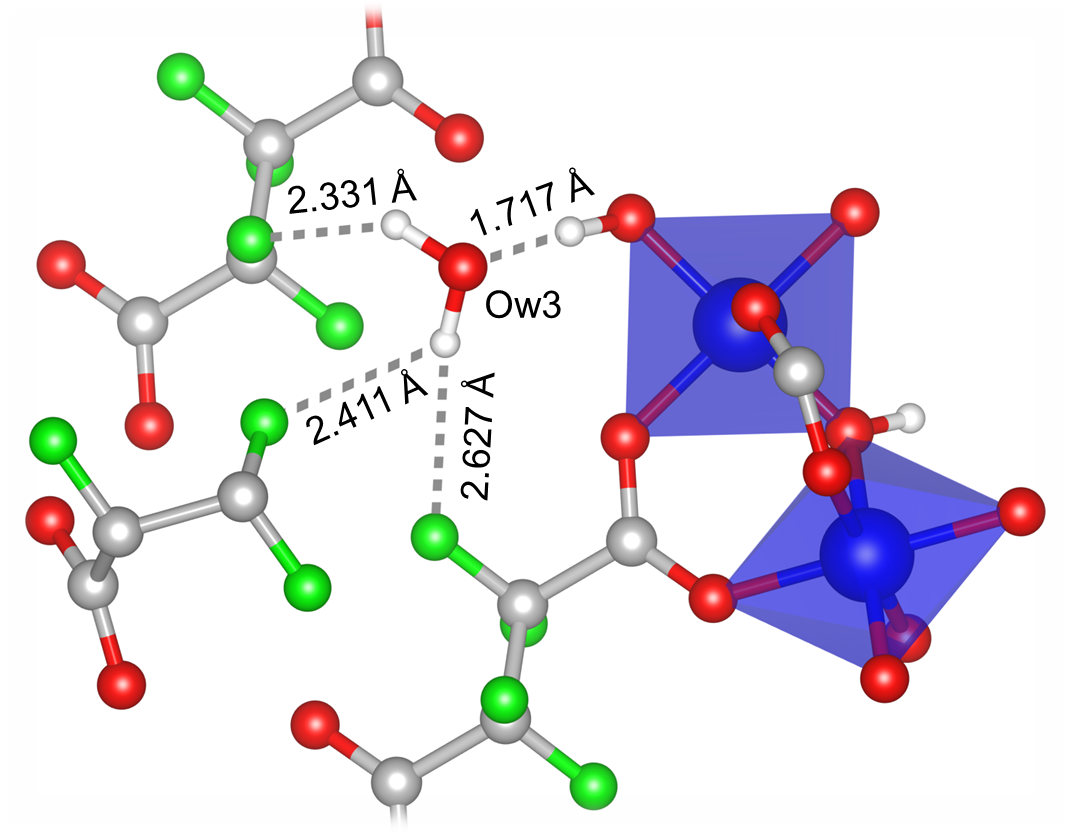
**

**Figure S14** The hydrogen bonding network involving Ow3 in as-synthesized Al-TFS from DFT-optimized models. Colour code: Al, blue; C, grey; O, red; F, green, H, white.


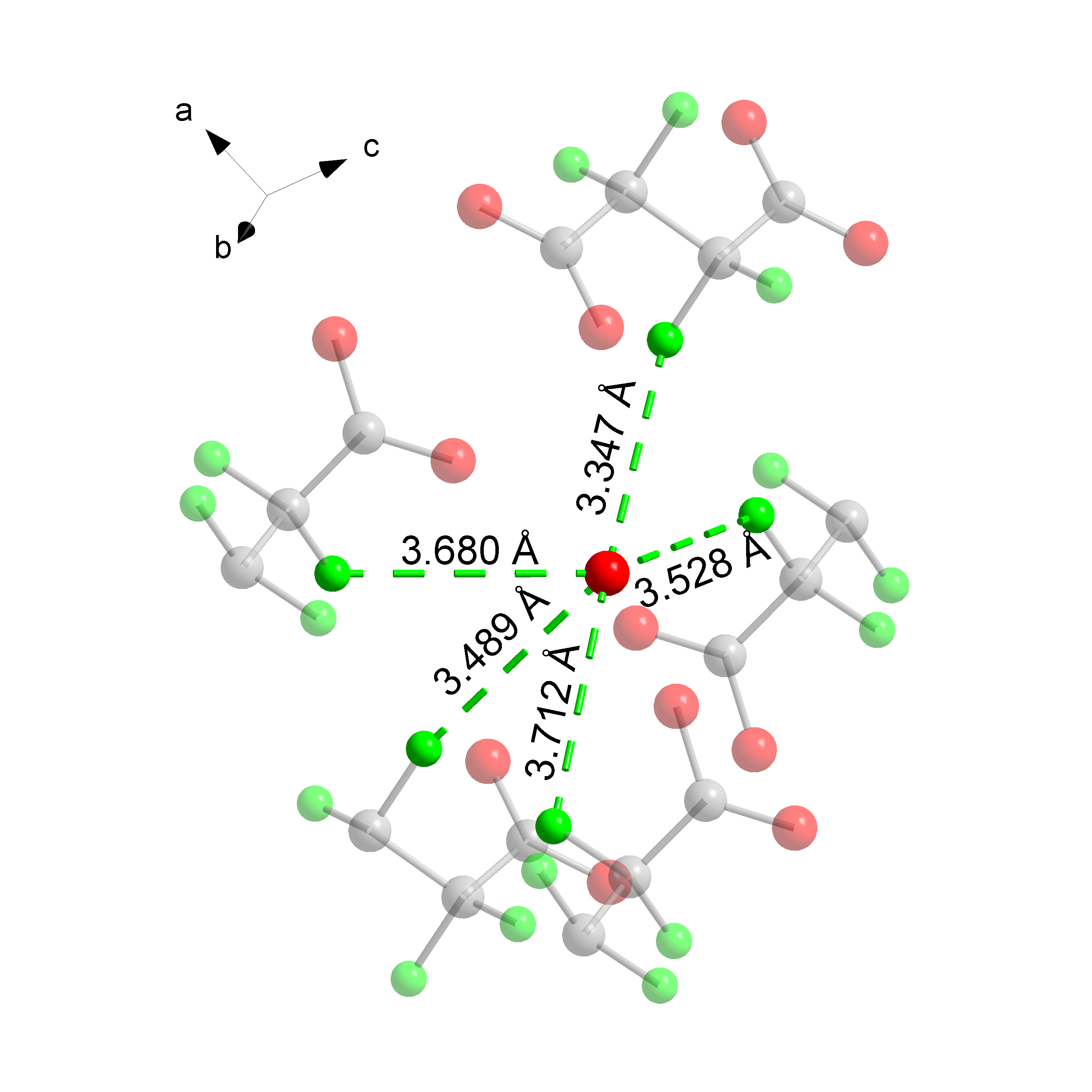


**Figure S15** Distances between Ow3 and the surrounding F atoms in as-synthesized Al-TFS. Atoms not involved in the interactions are shaded. Colour code: C, grey; O, red; F, green.


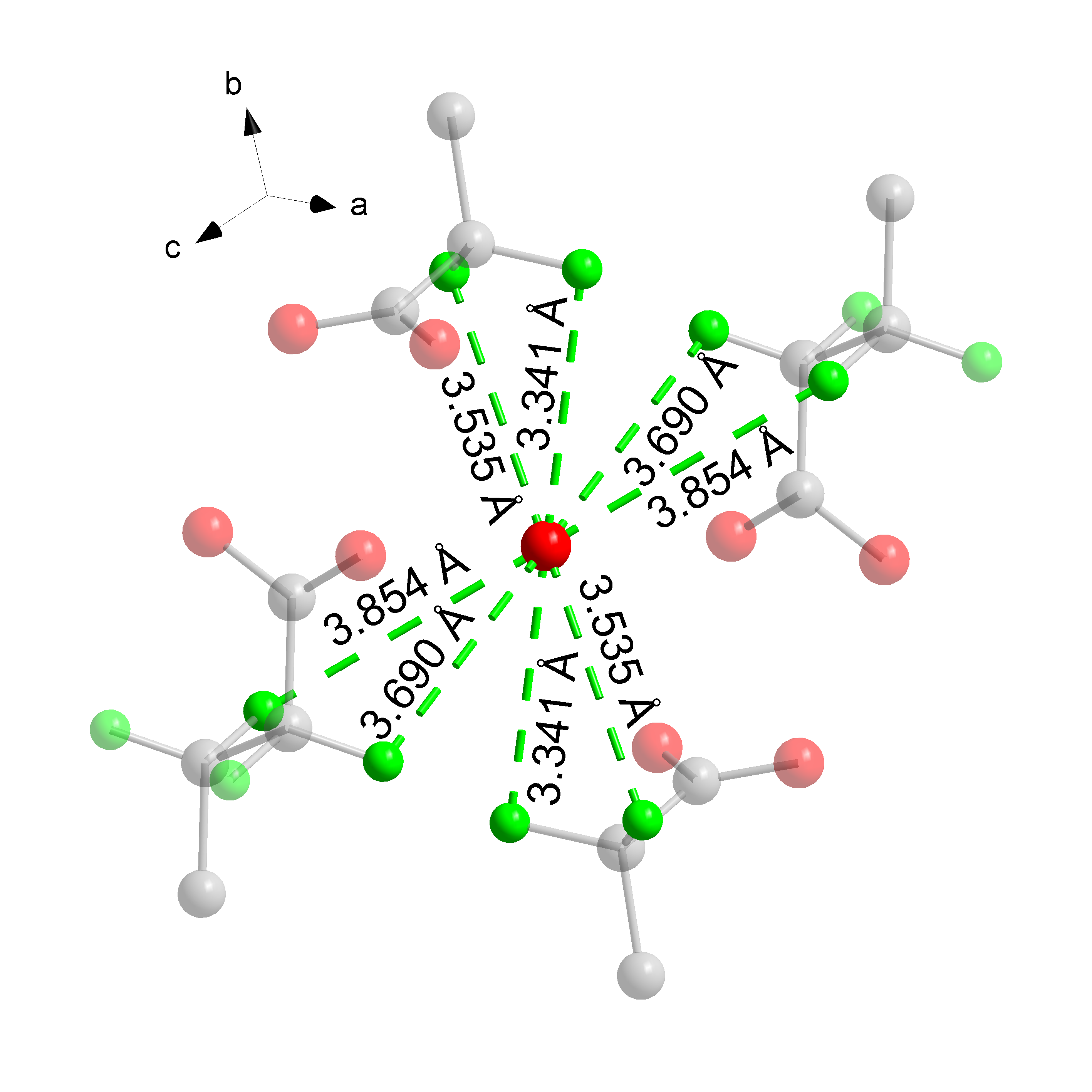


**Figure S16** Distances between Ow2 and the surrounding F atoms in as-synthesized Al-TFS. Atoms not involved in the interactions are shaded. Colour code: C, grey; O, red; F, green.





**Figure S17** VT-PXRD patterns in the 313-713 K range. A pattern at 303 K was collected at the end of the analysis. The small bump at about 7 °2θ is due to the Kapton foil of the high-temperature chamber.


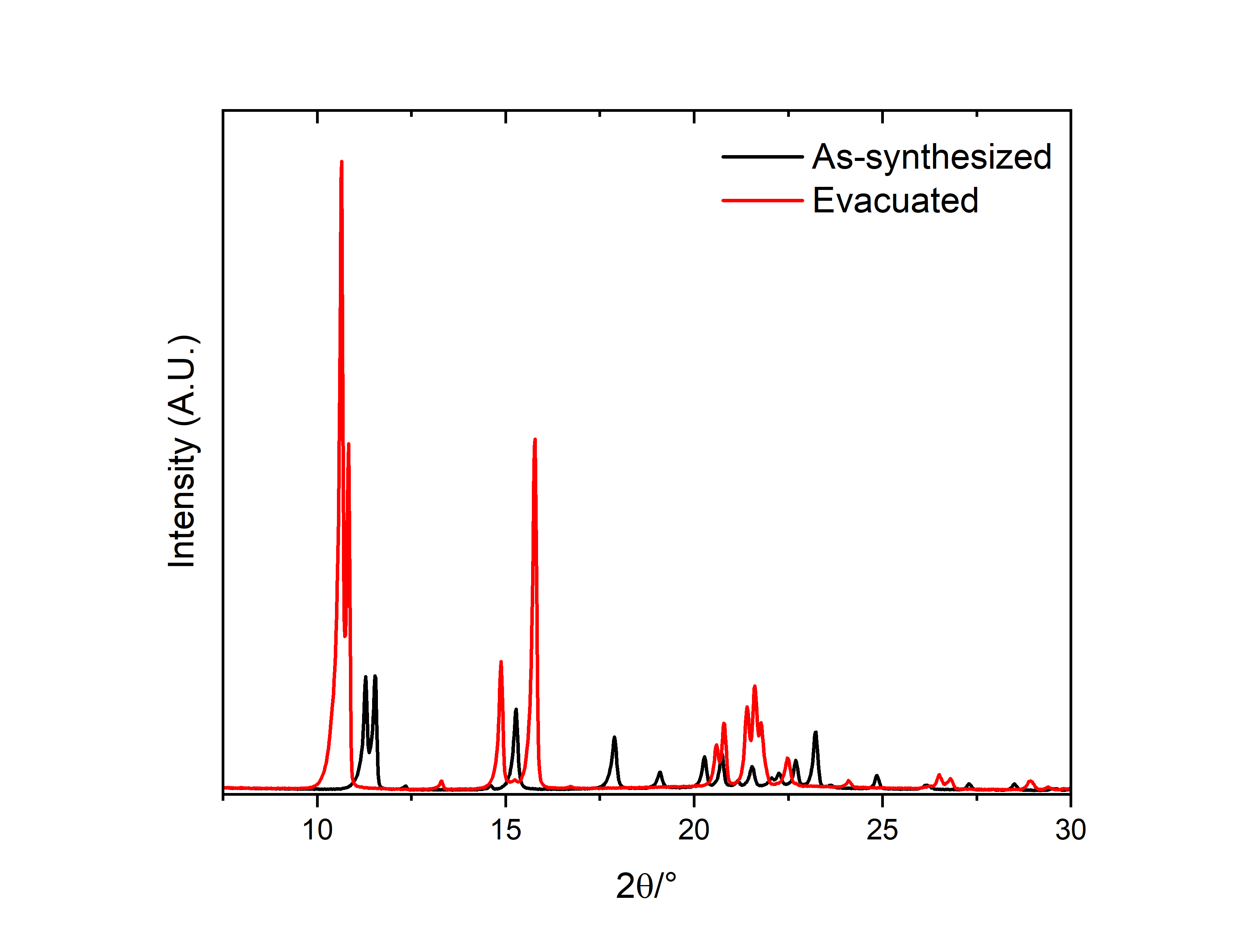


**Figure S18** Comparison of the PXRD patterns of as-synthesized (black) and evacuated (red) Al-TFS.


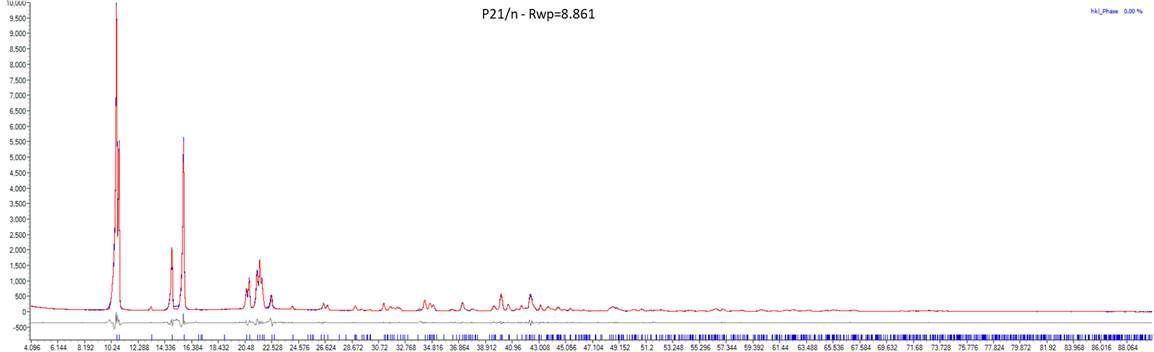


**Figure S19** Pawley refinement of the PXRD pattern of evacuated Al-TFS in the monoclinic P2_1_/n space group.


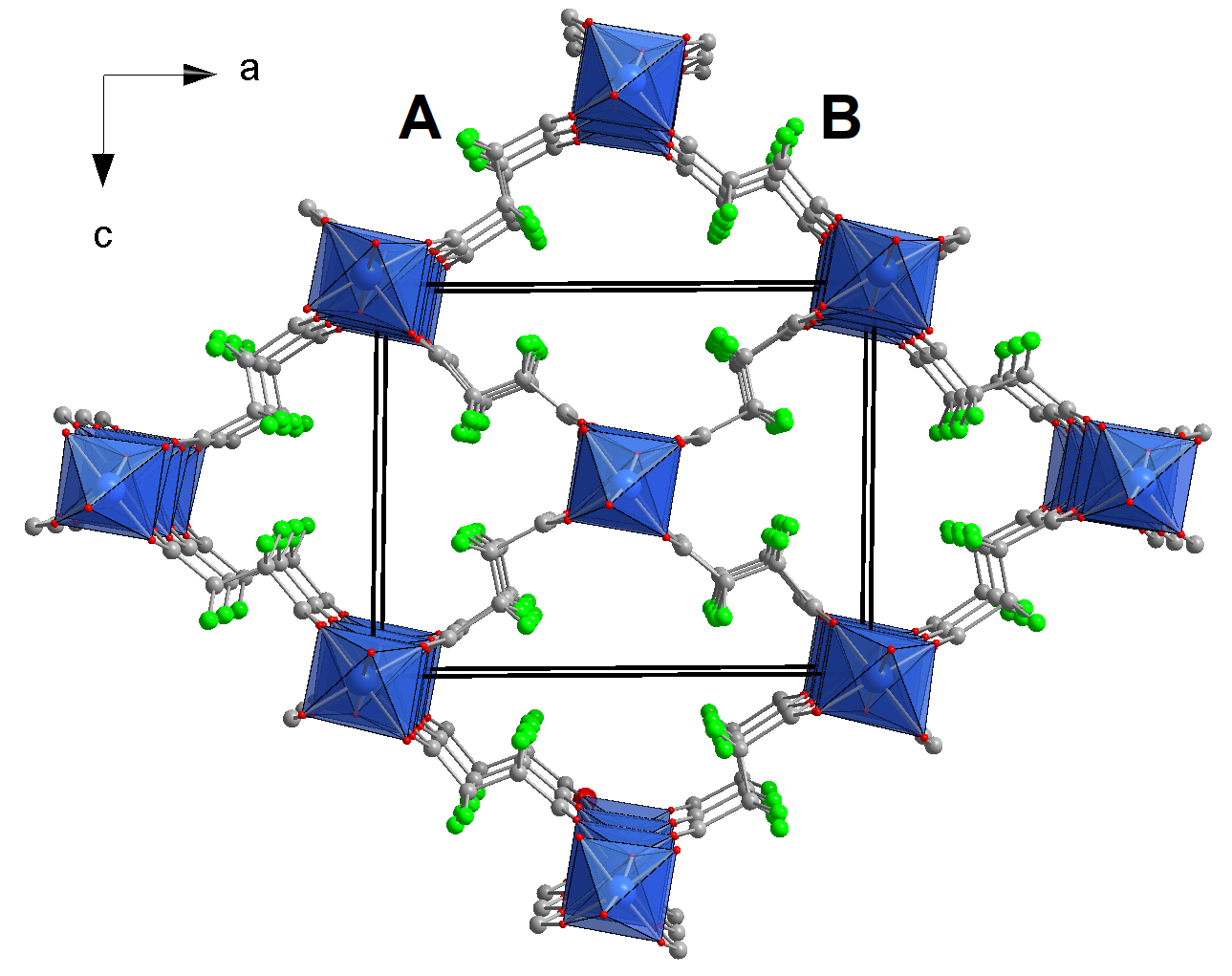





**Figure S20** Top: structural model of evacuated Al-TFS obtained from FOX viewed along the a axis. Color code: Al, blue; C, grey; O, red; F, green. Bottom: comparison of the experimental PXRD pattern (black) and that calculated from the proposed structural model (red).

1. *In situ* IR spectroscopy


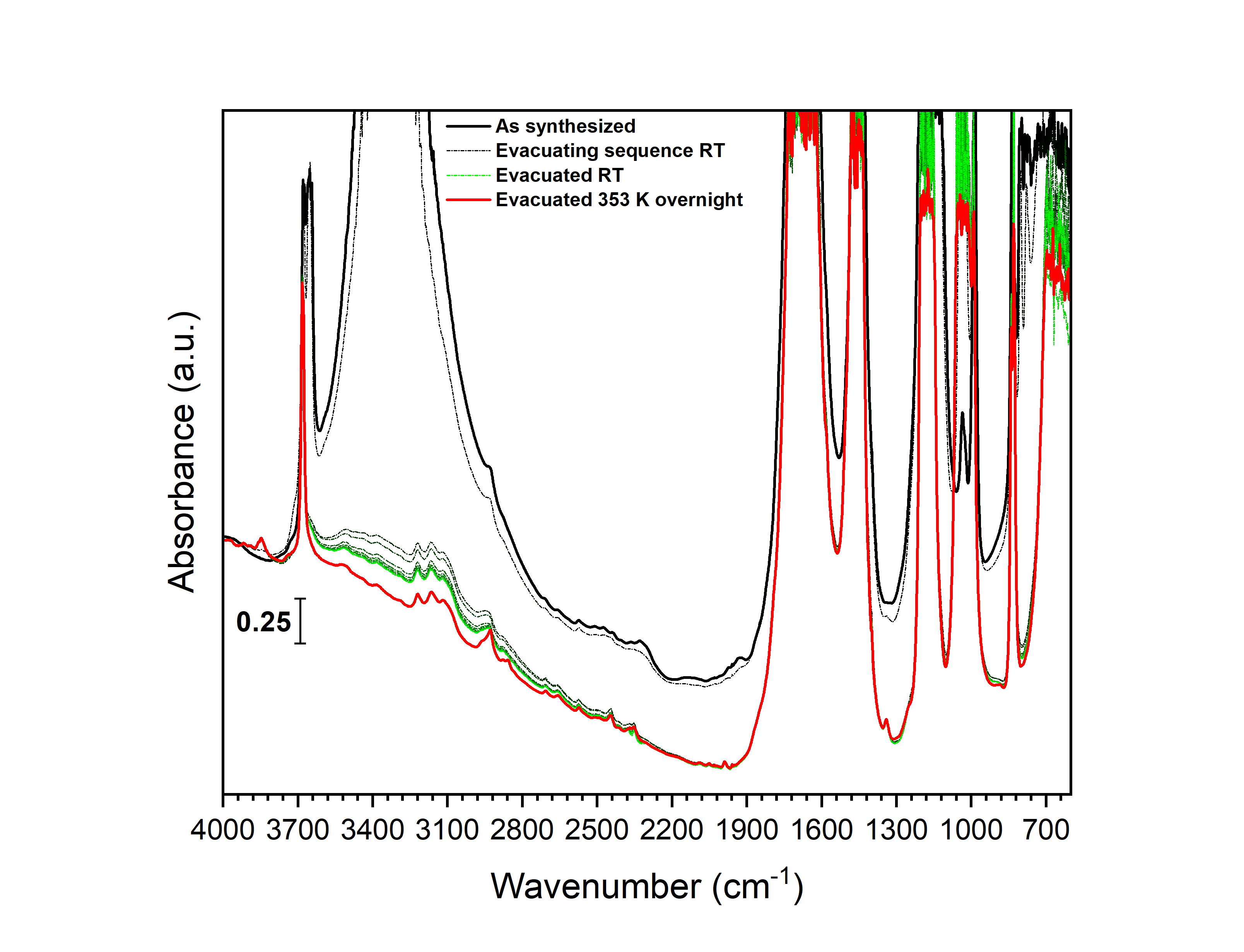


**Figure S21** IR spectra in the mid-IR range collected at room temperature on Al-TFS during activation: as-synthesized material (black curve); outgassing sequence performed at room temperature (RT) (grey dotted curves); last spectrum of the evacuating sequence at RT (light green curve); sample activated at 353 K overnight (red curve).


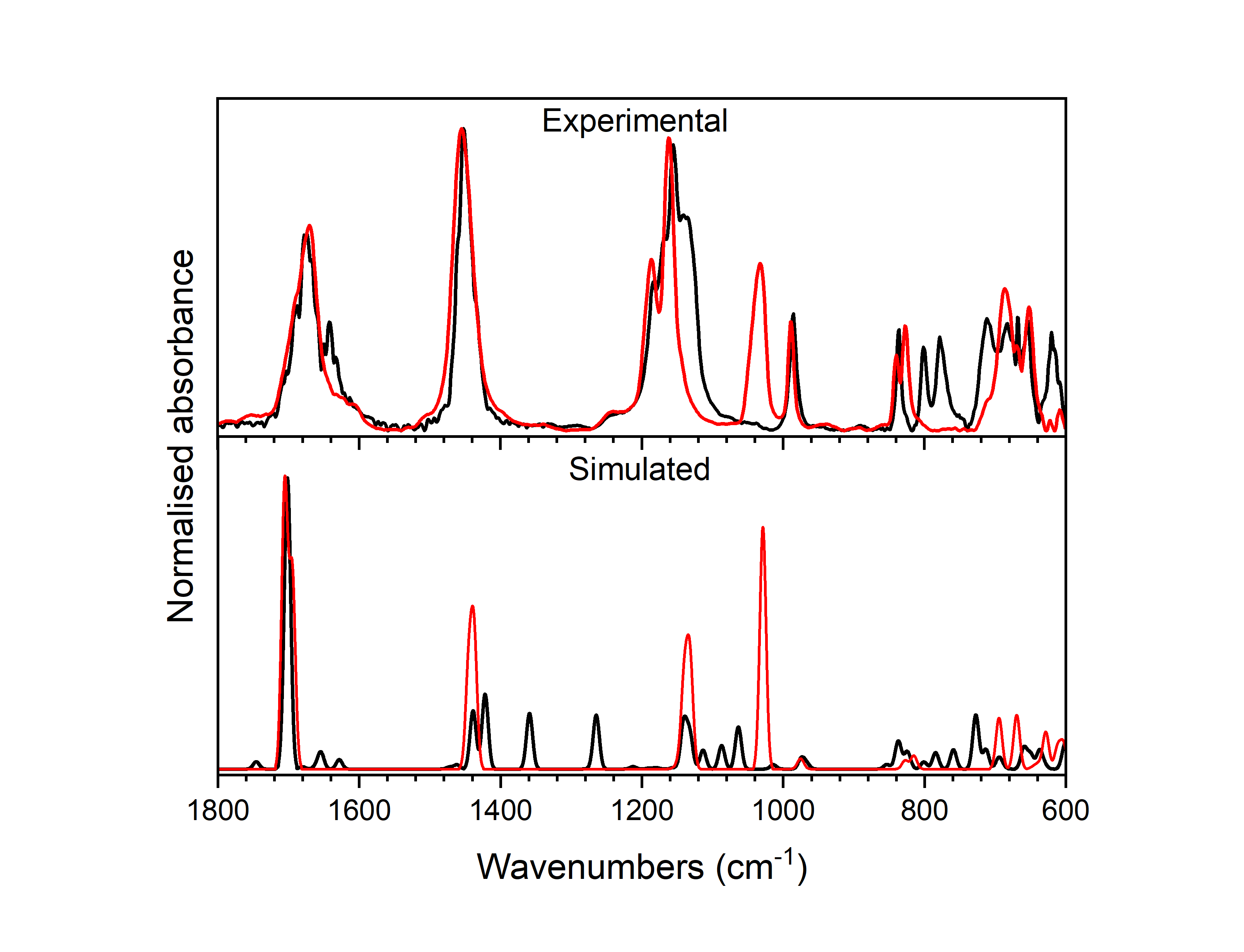


**Figure S22.** Comparison of experimental DRIFT (top) and simulated IR (bottom) spectra for the as-synthesized (black) and evacuated (red) phases of Al-TFS in the 1800 - 600 cm^−1^ spectral range.

Only two contributions in the simulated spectra of as-synthesized Al-TFS, located at 1265 and 1359 cm^-1^, respectively (see Figure S22), strongly deviate from the experimental spectrum. The discrepancy is ascribable to the strong anharmonicity of the corresponding vibrational modes, which cannot be reproduced within the harmonic approximation framework. Indeed, the mentioned contributions arise from bending modes of the -OH group strongly interacting with the water molecules network through hydrogen bonding. The differences between the spectra of the evacuated and as-synthesized phases that are not discussed in the main text are due to vibrational modes strongly influenced by the perturbation caused by the water molecule proximity (e.g., the sharp band located at 1031 cm^-1^ in the spectrum of the evacuated phase corresponds to the bending mode of the free -OH group of Al-TFS, which rises as water is removed from the pores). These features are therefore indicative of the hydration state rather than a phase transition.

**Optimized structure of as-synthesized Al-TFS in VASP POSCAR format:**

As-synthesized Al-TFS

1.00000000000000

6.6600460744190197 -0.0039406299934812 0.0810361493713436

-2.1842942769856344 8.7141684128995784 0.0691960288717906

-3.0196759037138277 -4.0739000220397825 7.2005713237852493

H C O F Al

6 8 12 8 2

Direct

0.9030533041982033 0.7161255531593756 0.6867007809156856

0.0969467178017912 0.2838744768406197 0.3132992490843160

0.0447980983087607 0.0089120513428516 0.1372601307370131

0.9552019166912357 -0.0089120733428526 0.8627398842629846

0.8630832293692686 0.0169881022536953 0.2081644294205838

0.1369167856307282 0.9830118897463036 0.7918355705794166

0.6541830084996244 0.2217473686450938 0.4954501726589440

0.3458170215003769 0.7782526173549049 0.5045497983410557

0.6261242773004179 0.0590205468218424 0.5267678608260801

0.3738756926995793 0.9409794301781519 0.4732321091739233

0.4142309657586311 0.5830151416070144 0.8180760515461136

0.5857690342413677 0.4169848283929841 0.1819239634538839

0.5054585610284700 0.5905444159736989 0.0082650811114280

0.4945414389715314 0.4094556140262964 0.9917349428885734

0.8301966047008064 0.2746397945884414 0.4729132460004875

0.1698034252991954 0.7253602054115580 0.5270867839995017

0.5040050958091028 0.2833339333895358 0.4979566090321778

0.4959948741909022 0.7166660666104644 0.5020433609678199

0.5564574989756749 0.5903011492340250 0.7528901482655270

0.4435425010243247 0.4096988507659740 0.2471098367344758

0.2059411804692251 0.5653893963399428 0.7486128346259696

0.7940588345307728 0.4346106036600581 0.2513871653740313

0.8172306224162466 0.5935887085624804 0.5836858115132265

0.1827693925837506 0.4064112914375197 0.4163141584867712

0.9405928349262339 0.0669187325078064 0.1504666921012600

0.0594071800737639 0.9330812904921917 0.8495333228987371

0.7142127239305264 0.9607958865273870 0.4321296974981303

0.2857872760694734 0.0392041434726085 0.5678702725018746

0.7614376104395936 0.1191900699471299 0.7119146712553097

0.2385623895604059 0.8808099600528730 0.2880853287446897

0.7349122226346707 0.7100120922917416 0.1198435274499611

0.2650877623653244 0.2899879377082614 0.8801564725500384

0.3762815287829727 0.6406898220229953 0.0916685259495734

0.6237184712170192 0.3593101779770049 0.9083314890504253

0.5000000000000000 0.5000000000000000 0.5000000000000000

0.0000000000000000 0.5000000000000000 0.5000000000000000

**Optimized structure of evacuated Al-TFS in VASP POSCAR format:**

Evacuated Al-TFS

1.00000000000000

13.3022905826520876 0.0000000000000000 -0.0432806789851063

0.0000000000000000 6.6957616936261237 0.0000000000000000

-0.2223840100504613 0.0000000000000000 10.3291547403876116

H C O F Al

4 16 20 16 4

Direct

0.5470169064892760 0.6813543797188309 0.3421921631881920

0.4529830935107244 0.1813543797188308 0.6578078368118082

0.9529491207605570 0.6531330981490628 0.1578284958793043

0.0470508682394455 0.1531330981490629 0.8421714741207004

0.1586736636815715 0.6535364122712541 0.8972516613305915

0.8413263513184263 0.1535364122712541 0.1027483386694082

0.2594709239663683 0.6543424818381709 0.8251559009650975

0.7405290460336360 0.1543424818381709 0.1748440990349023

0.2410020783626026 0.6788586683885421 0.6758176149714803

0.7589979366373951 0.1788586683885424 0.3241823850285127

0.3416118566782577 0.6803795346628484 0.6034242895969945

0.6583881133217393 0.1803795346628482 0.3965757104030055

0.3907189194277023 0.1794835547487469 0.3137684954919623

0.6092811105722931 0.6794835407487458 0.6862314745080424

0.3012601664524590 0.1763242785454955 0.2147743212881441

0.6987398335475409 0.6763242935454931 0.7852256937118537

0.1986086994592411 0.1572558491999999 0.2852462005201246

0.8013913305407621 0.6572558641999978 0.7147537704798768

0.1090578272116727 0.1544411614492907 0.1863771320231312

0.8909421647883268 0.6544411464492931 0.8136228529768709

0.1231120173727813 0.8235750207962801 0.9213186354948391

0.8768879826272187 0.3235750207962803 0.0786813645051610

0.1214394977993631 0.4839396404718546 0.9182128639152259

0.8785605242006388 0.9839396104718523 0.0817871360847735

0.3786493034311253 0.8501321627831677 0.5823762575455984

0.6213506965688673 0.3501321627831676 0.4176237424544016

0.3772723027875998 0.5105144131202879 0.5791345230031314

0.6227276972124003 0.0105144131202877 0.4208654769968684

0.4218596995503067 0.0102290076890768 0.3502780589992743

0.5781403304496957 0.5102290226890814 0.6497219710007284

0.4201267348040612 0.3501081166354355 0.3497195971961886

0.5798732651959387 0.8501081166354354 0.6502804028038114

0.0777456974991796 0.3237477711588654 0.1501628883027795

0.9222542875008223 0.8237477711588579 0.8498371116972205

0.0796713614038206 0.9839342097230226 0.1502389463427453

0.9203286385961792 0.4839342247230199 0.8497610536572549

0.9844790993136658 0.6535532975283946 0.0741664643923177

0.0155209056863384 0.1535532975283943 0.9258335276076822

0.4845994961102874 0.1807008818809795 0.5741966267485940

0.5154005038897134 0.6807008818809799 0.4258033732514056

0.3106803272155989 0.4800269100134408 0.8471498418626685

0.6893196727844018 0.9800268800134386 0.1528501581373318

0.3190825423457416 0.8091324235184022 0.8675707302092720

0.6809174876542609 0.3091324235184022 0.1324292697907277

0.1895186595553947 0.8528677302104887 0.6539254315151171

0.8104813254445935 0.3528677302104888 0.3460745684848829

0.1816138112733234 0.5237318998224002 0.6333991083759906

0.8183861747266752 0.0237318998224001 0.3666008916240091

0.3098154877992908 0.0177626402412405 0.1320047453332263

0.6901845122007094 0.5177626182412384 0.8679952396667693

0.2996999485331532 0.3477687981781238 0.1428621684463885

0.7003000514668469 0.8477688281781259 0.8571378465536087

0.2001192944125021 0.9856515217450846 0.3569699984270011

0.7998807055874977 0.4856515137450839 0.6430300015729995

0.1901266933267293 0.3155577996130921 0.3682162749554343

0.8098733066732708 0.8155577996130917 0.6317837550445684

0.9995246117463272 0.4037029600667741 0.9995266816214222

0.0004753772536751 0.9037029310667755 0.0004733313785750

0.4995988870594317 0.4304533888313437 0.4993276613856055

0.5004011129405684 0.9304533888313441 0.5006723676143935

1. SSNMR spectroscopy


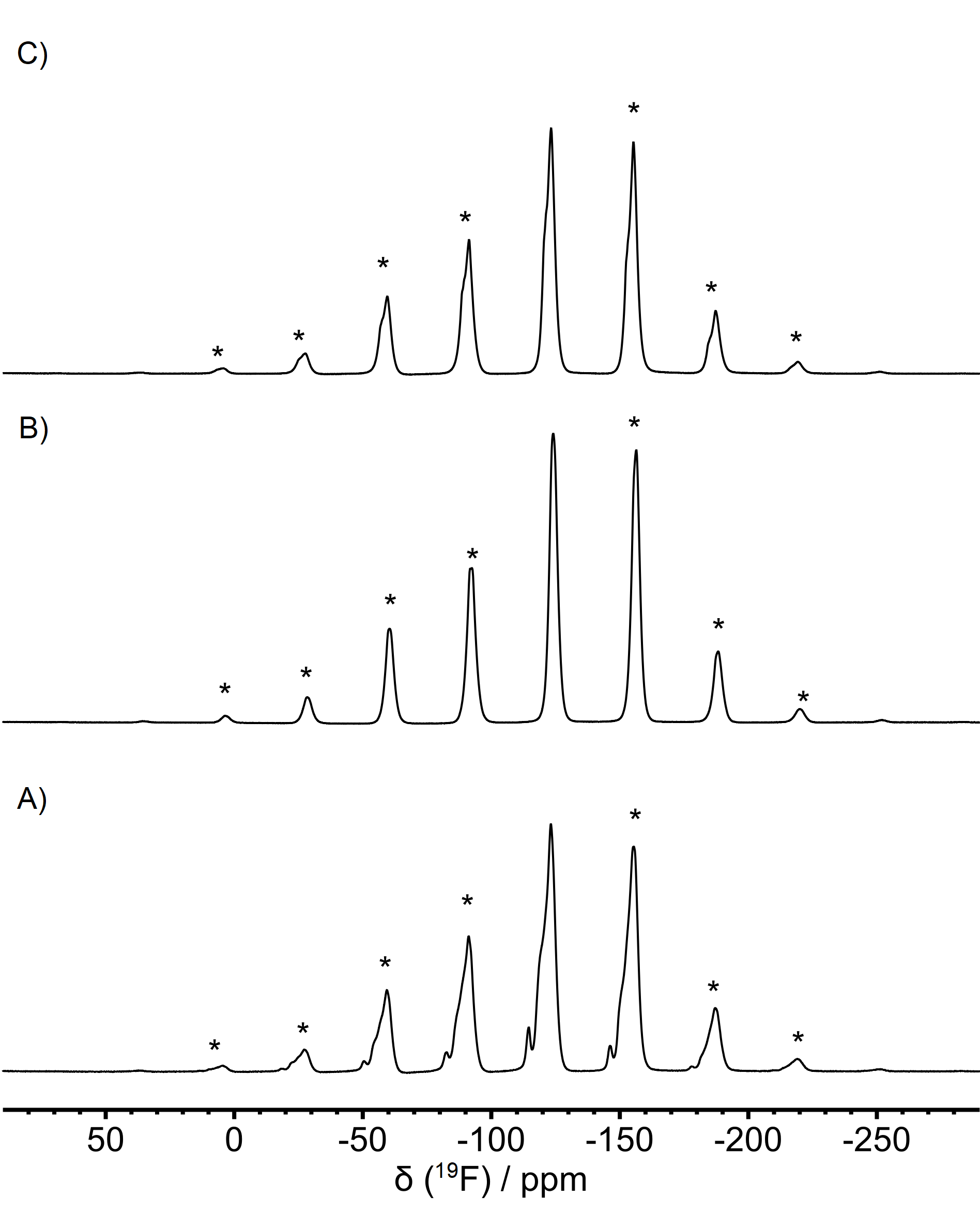


**Figure S23** ^19^F DE MAS spectra of as-synthesized (A), evacuated (B), and CO_2_-loaded (C) Al-TFS. Spectra were recorded at a spinning frequency of 15 kHz. Asterisks indicate spinning sidebands.


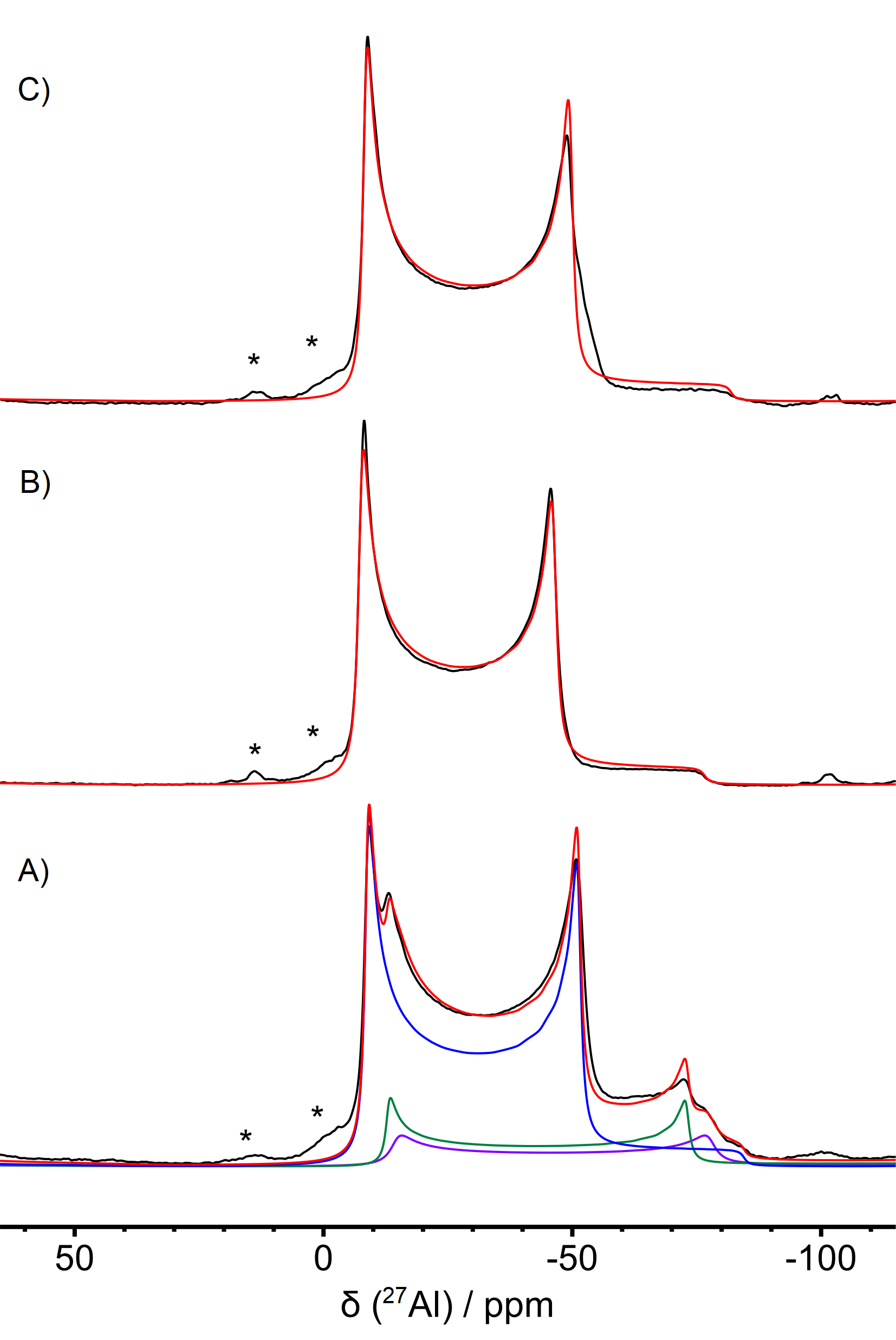


**Figure S24** Experimental (black) and simulated (red) ^27^Al DE MAS spectra of as-synthesized (A), evacuated (B), and CO_2_-loaded (C) Al-TFS. Spectra were recorded at a spinning frequency of 15 kHz. For as-synthesized Al-TFS, the three sub-spectra used to reproduce the spectrum are also shown as green, blue and purple lines. Asterisks indicate signals from Al hydroxide/oxide impurities.


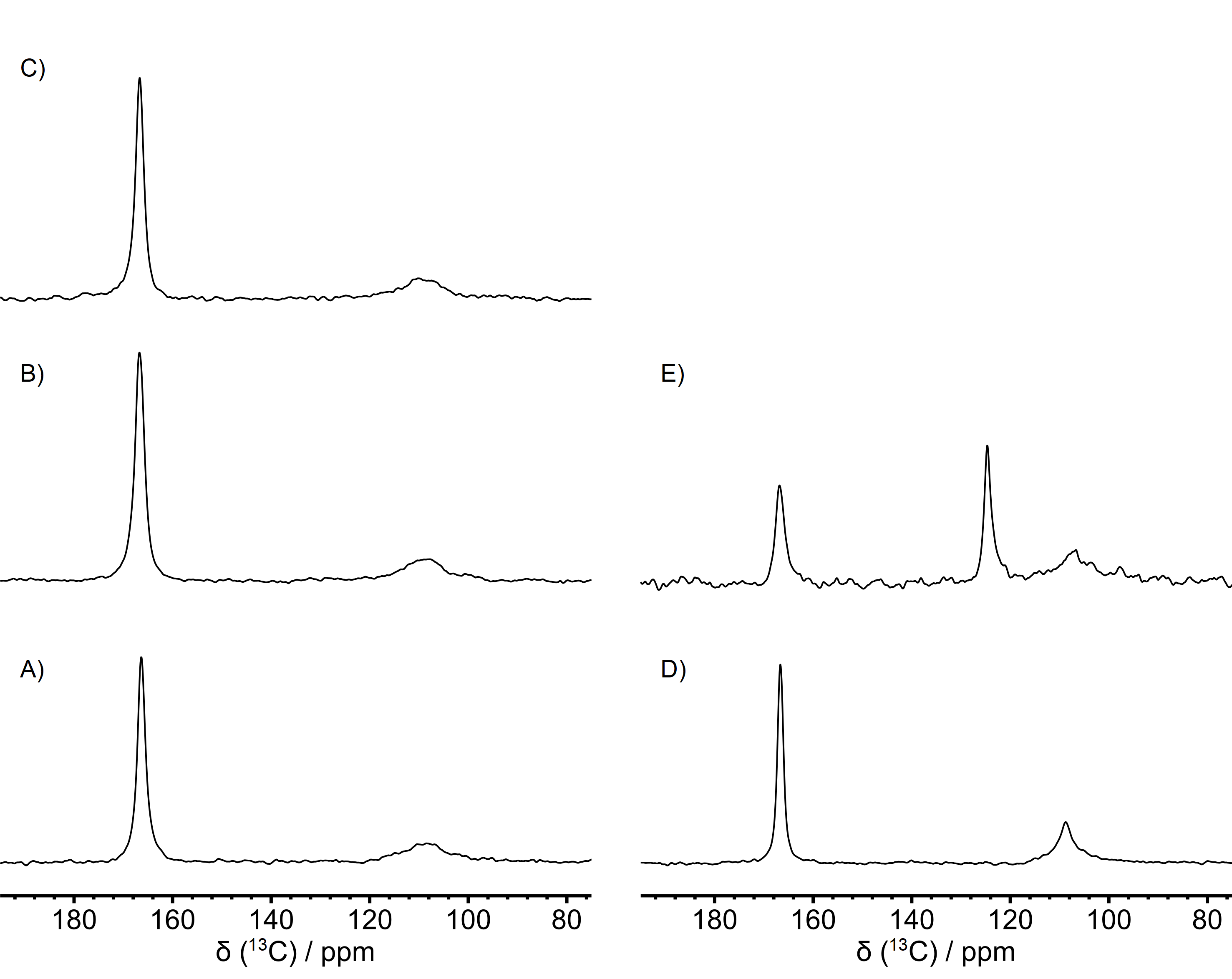


**Figure S25** ^1^H-^13^C CP MAS spectra of as-synthesized (A), evacuated (B), and CO_2_-loaded (C) Al-TFS recorded with a contact time of 8 ms. ^19^F-^13^C CP MAS spectrum of CO_2_-loaded Al-TFS recorded with a contact time of 8 ms (D). ^13^C DE MAS spectrum of CO_2_-loaded Al-TFS recorded with a recycle delay of 5 s (E). All the spectra were recorded at a spinning frequency of 15 kHz.

1. Evaluation of the textural properties


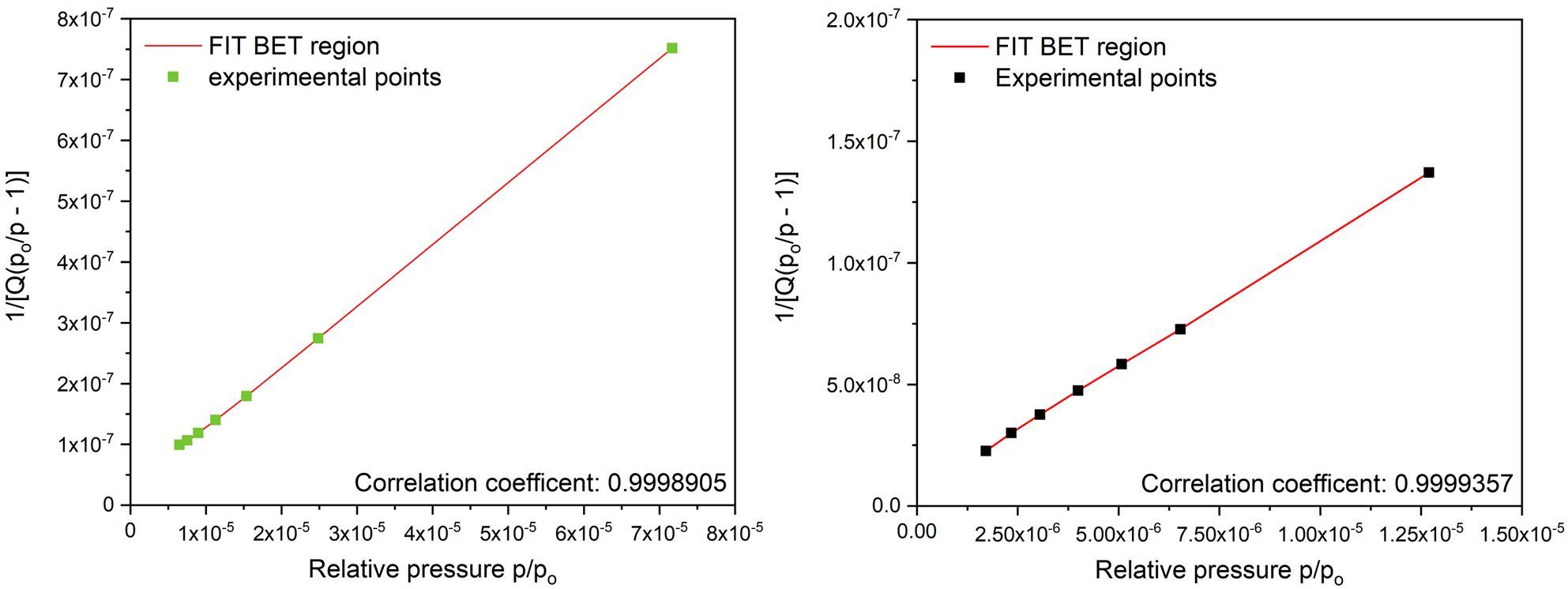


**Figure S26** Left) Linear BET fit for Ar isotherm in the 6·10^-6^ – 8·10^-5^ p/p_o_ range; Right) Linear BET fit for N_2_ isotherm in the 1.5·10^-6^ – 1.6·10^-5^ p/p_o_ range.


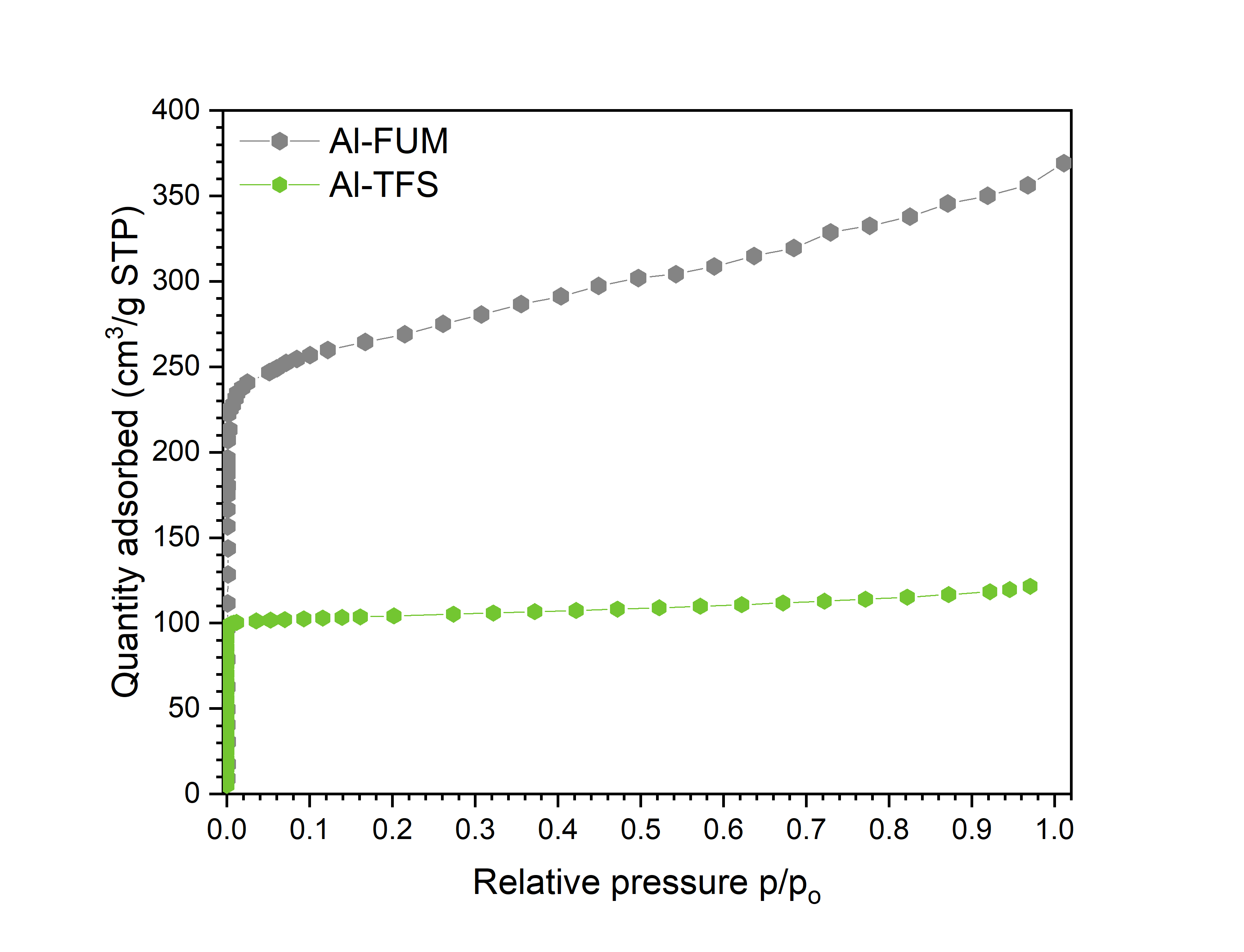


**Figure S27** Ar adsorption isotherms of Al-FUM^[1,2]^ (grey curve) and Al-TFS (green curve) collected at 87 K.

**Table S2** Specific Surface Areas of as-synthesized and evacuated Al-TFS calculated with the software Zeo++ using different probe dimensions. ASA = accessible surface area; NASA = not accessible surface area.


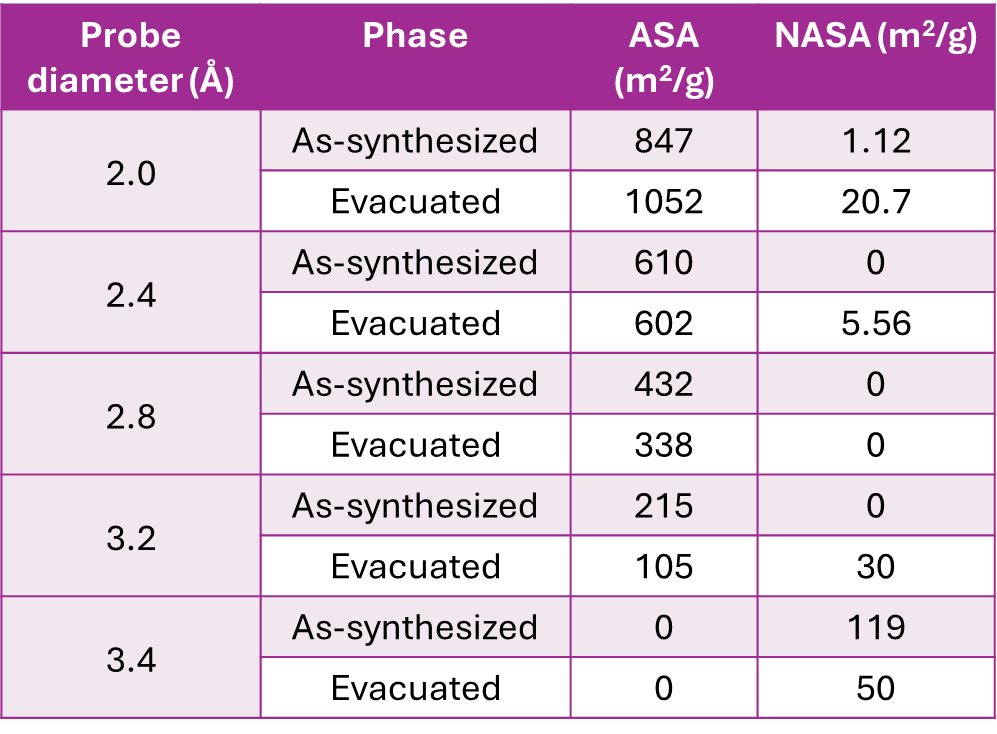


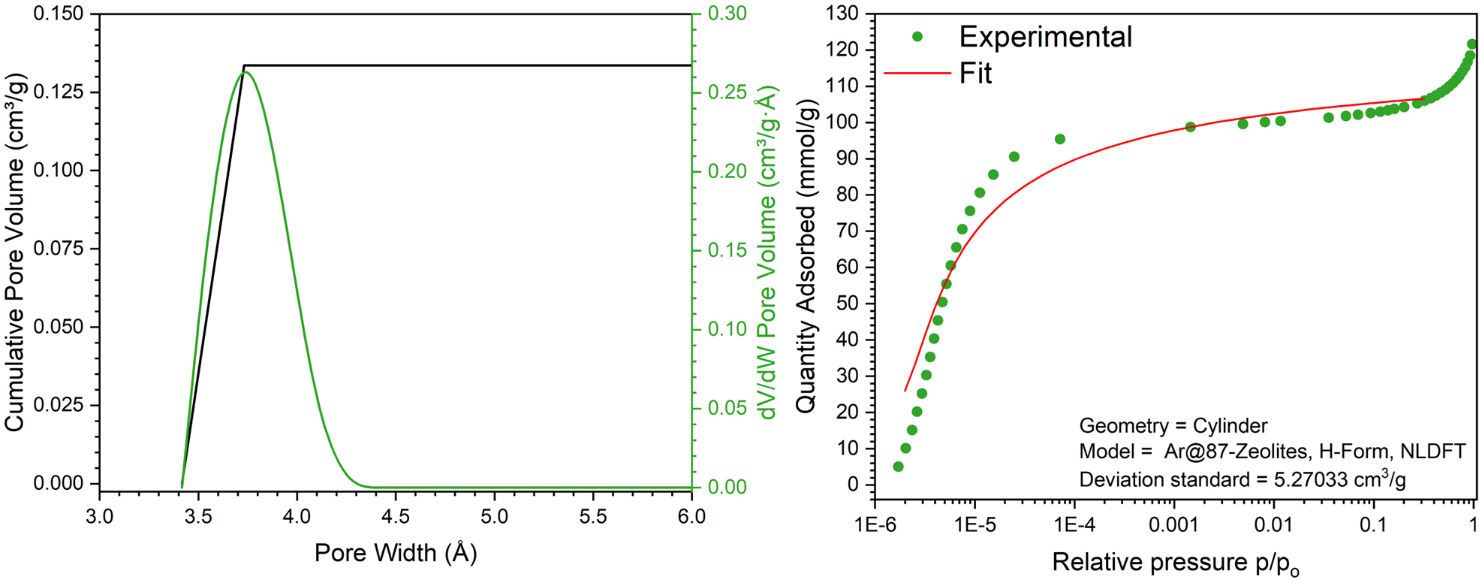


**Figure S28** Left) PSD (green curve) and CPV (black curve) of evacuated Al-TFS computed from the Ar adsorption isotherm at 87 K; Right) DFT Pore size Goodness of Fit Graph in semi-logarithmic scale with relative standard deviation values and models used for NL-DFT. Full green circles represent the experimental isotherm, red curve is the theoretical one.


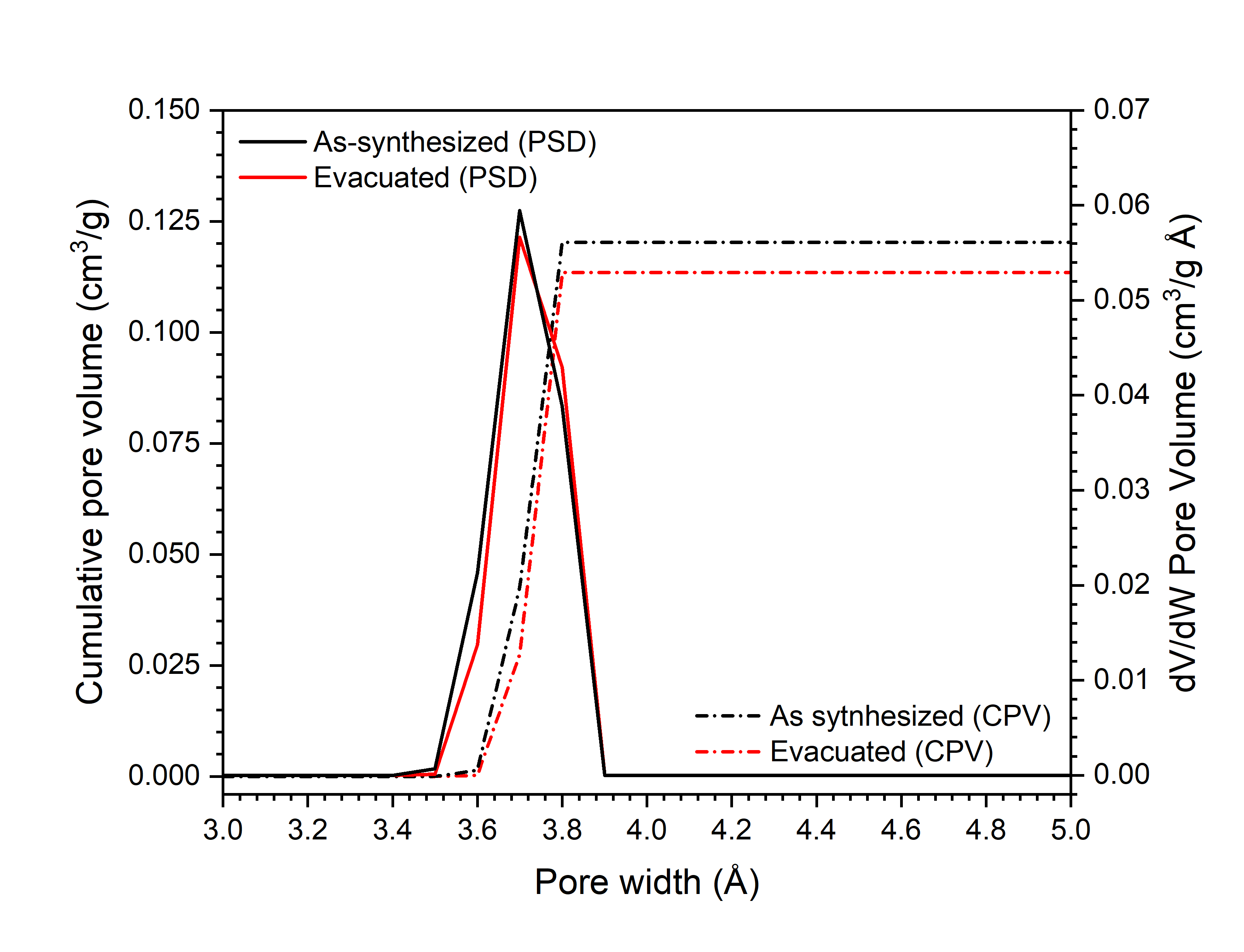


**Figure S29** PSD (full lines) and CPV (dot-dashed lines) of as-synthesized (black) and evacuated (red) Al-TFS calculated with the software Zeo++ using a probe diameter of 2.8 Å.

1. Evaluation of Al-TFS CO_2_ sorption performances

**Table S3** Comparison of the Henry’s constant (K_H_) calculated starting from the isotherm fits reported in Figure 7A of the main text (T = 273 K)

| Region number | *K*_H_ |
| --- | --- |
| I | 5.95 ± 1.71 |
| II | 11.17 ± 0.49 |


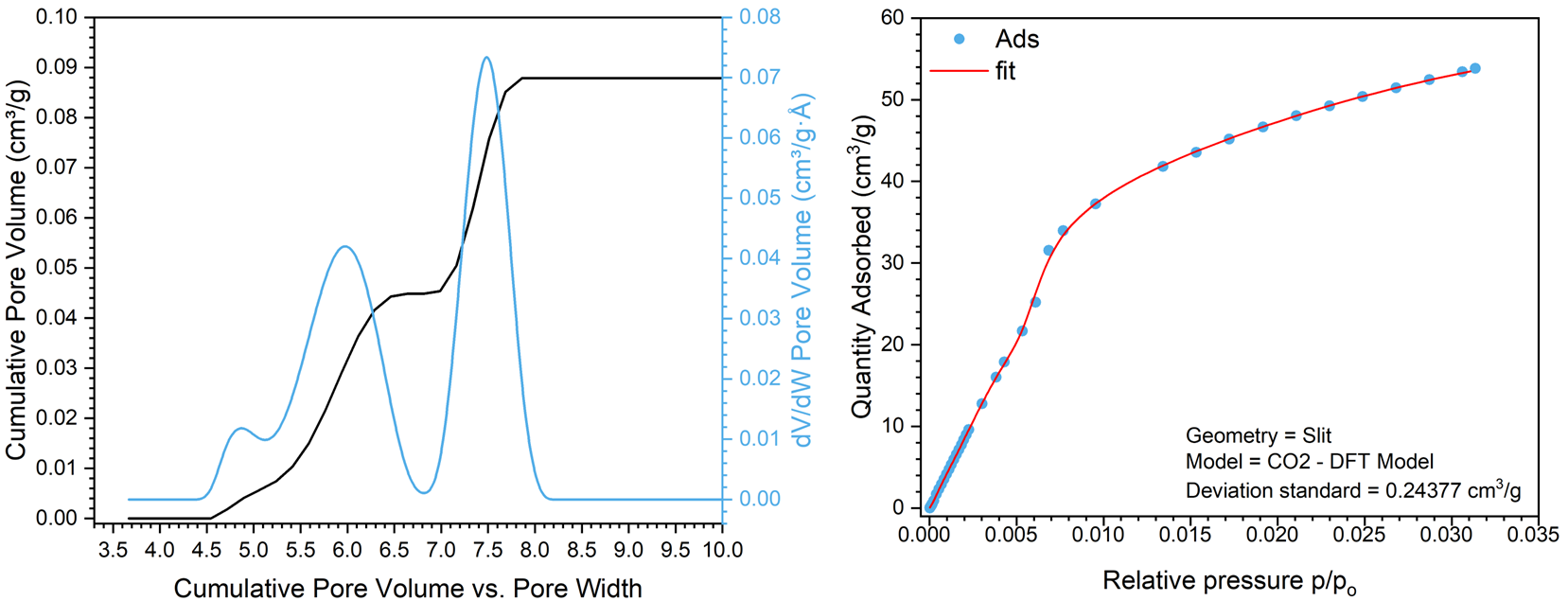


**Figure S30** Left) PSD (light blue curve) and CPV (black curve) of Al-TFS computed from CO_2_ adsorption/desorption isotherms; Right) DFT Pore size Goodness of Fit Graph with relative standard deviation values and models used for NL-DFT. Full light blue circles represent the experimental isotherm, red curve is the theoretical one.


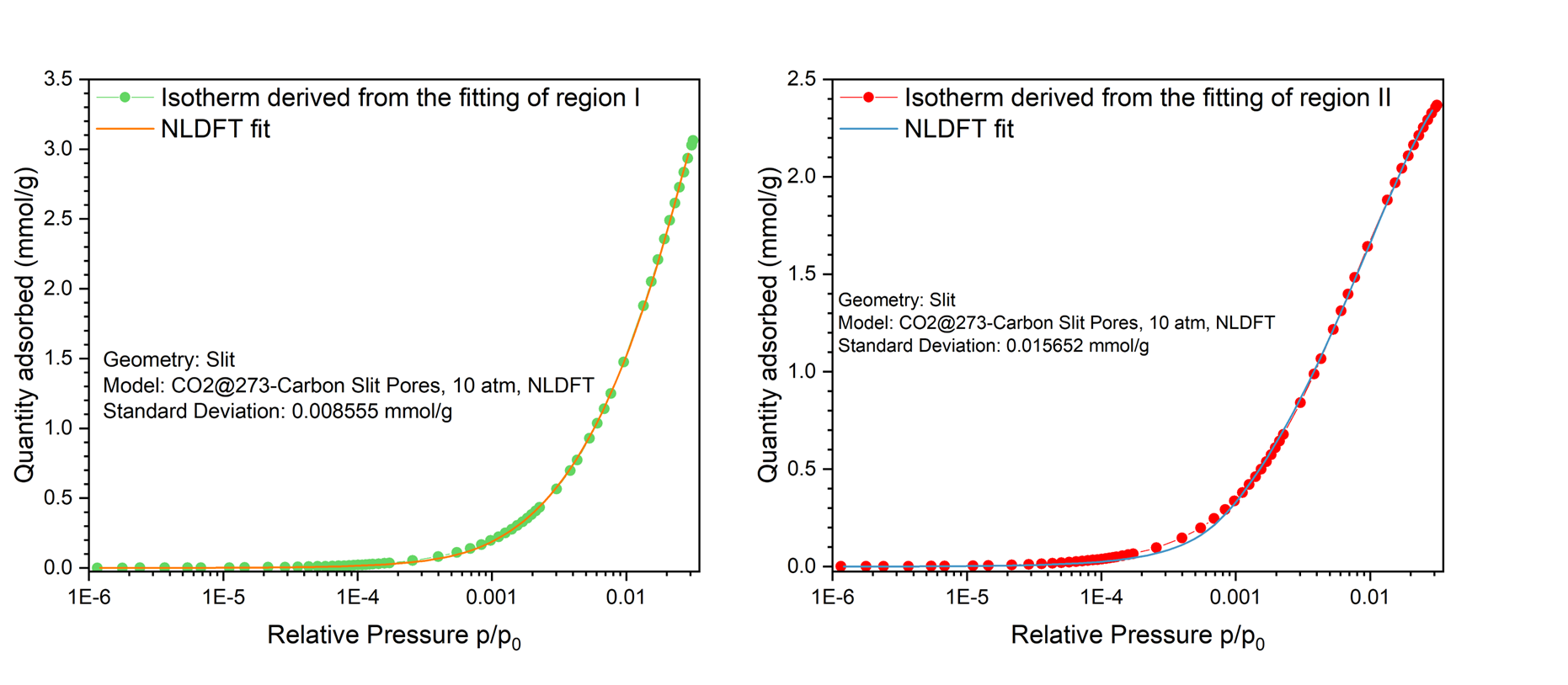


**Figure S31** DFT Pore size Goodness of Fit Graph, in semi-logarithmic scale, with relative standard deviation values and models used for NL-DFT. Full circles represent the experimental isotherms, solid lines represent the theoretical ones. Left) Isotherm derived from the fitting of region I of Figure 7A; Right) Isotherm derived from the fitting of region II of Figure 7A.





**Figure S32** Comparison of the PXRD patterns of as-synthesized (black), evacuated (red), and CO_2_-loaded (green) Al-TFS.


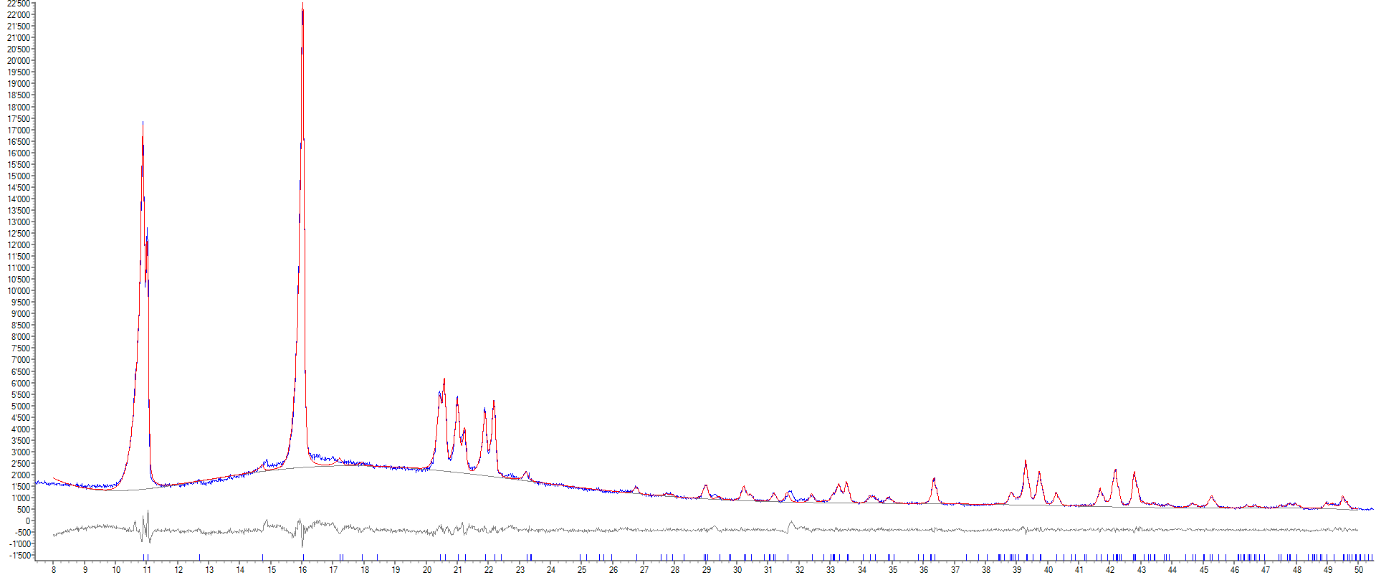


**Figure S33** Pawley refinement of the PXRD pattern of CO_2_-loaded Al-TFS in the P2_1_/n space group (Rwp = 5.02).


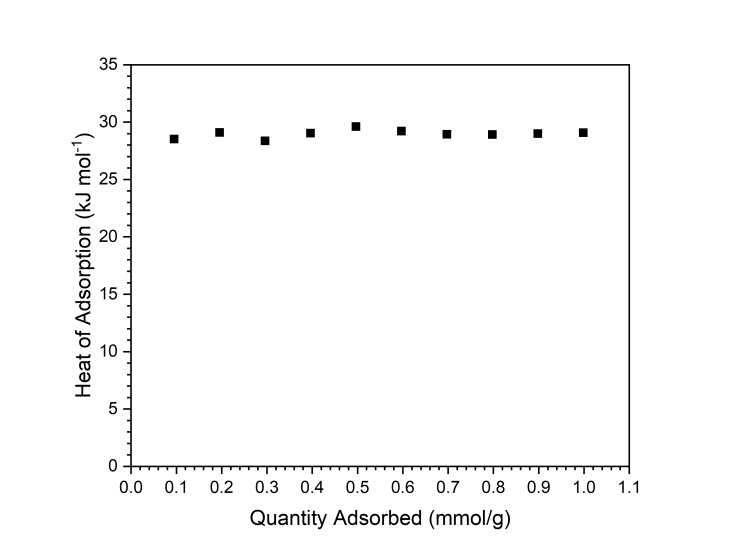


**Figure S34** CO_2_ isosteric heat of adsorption of Al-TFS as a function of the adsorbed CO_2_ amount


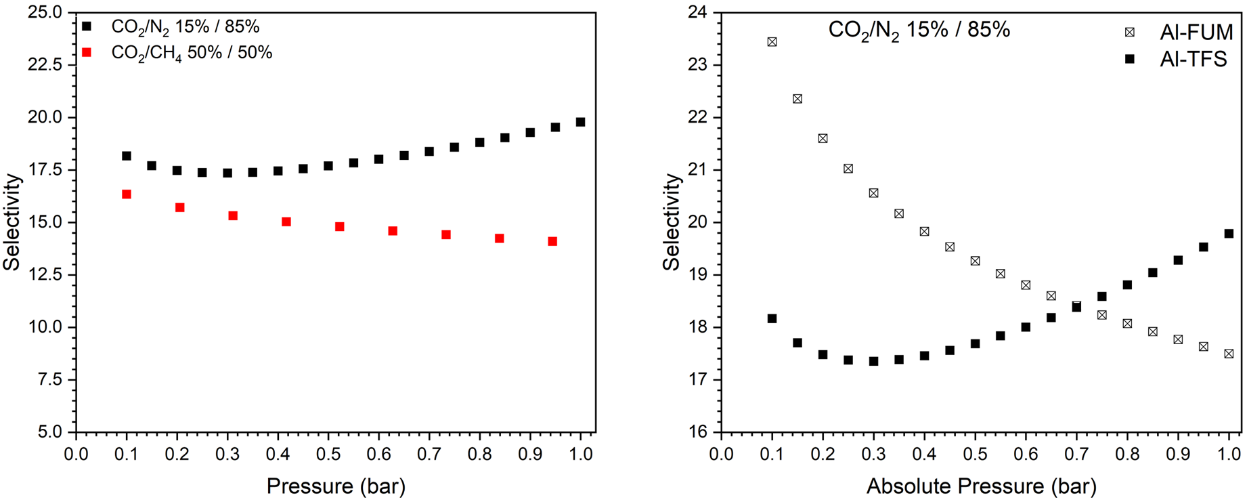


**Figure S35** Left) CO_2_/N_2_ selectivity (black squares) and CO_2_/CH_4_ selectivity (red squares) of Al-TFS. Right) Comparison between Al-TFS (full squares) and Al-FUM (crossed squares) CO_2_/N_2_ selectivity predicted for coal-derived flue gas compositions.

1. H_2_O sorption properties of Al-TFS


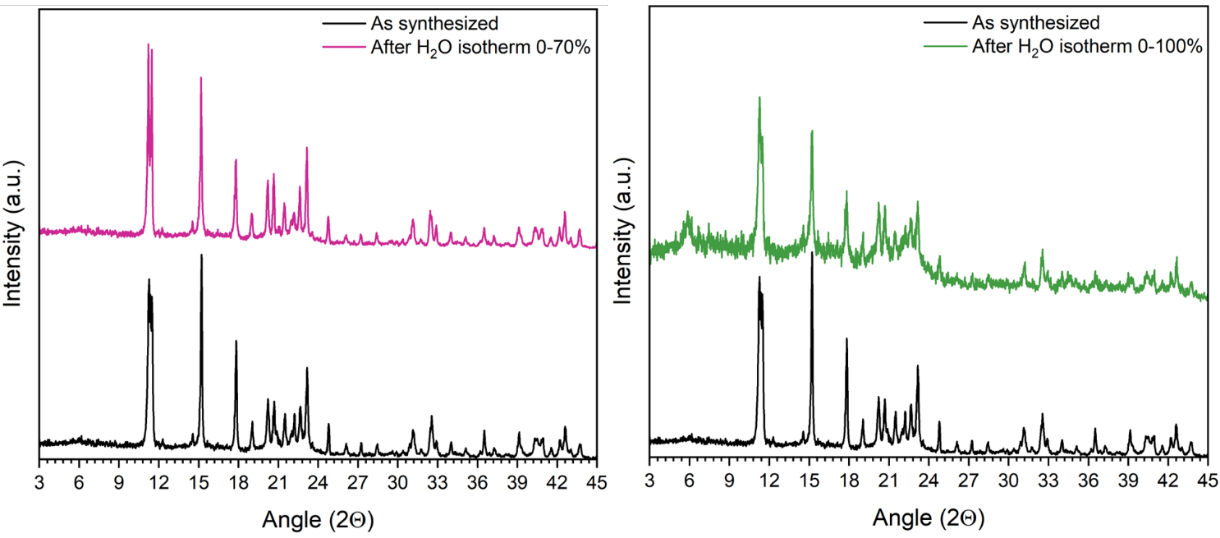


**Figure S36** Left) comparison between PXRD patterns of as-synthesized Al-TFS (black pattern) and of Al-TFS after H_2_O adsorption/desorption in the 0-70 % RH range (magenta pattern); Right) comparison between the PXRD patterns of as-synthesized Al-TFS and of Al-TFS after water adsorption/desorption in the 0-100 % RH range (green pattern).

1. DFT calculations


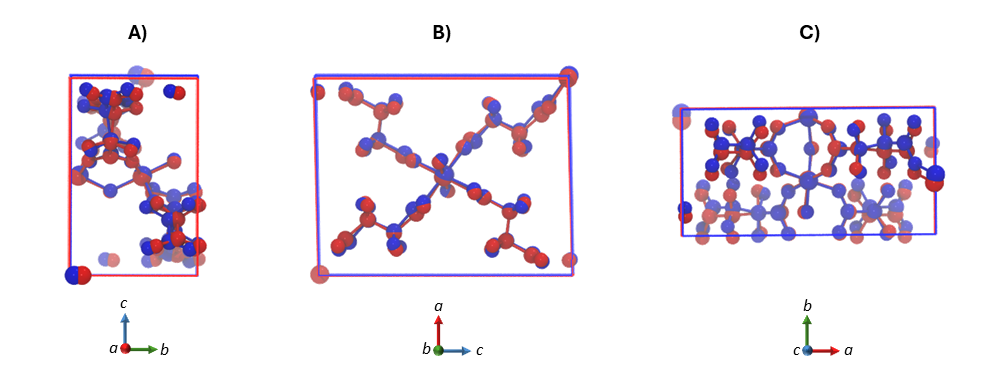


**Figure S37** Unit cell for the structural model of evacuated Al-TFS obtained with FOX (blue) and subsequently optimized by DFT (red), viewed along: A) a axis; B) b axis; and c) c axis.

1. References

[1] B. Bozbiyik, J. Lannoeye, D. E. De Vos, G. V Baron, J. F. M. Denayer, *Physical Chemistry Chemical Physics* **2016**, *18*, 3294–3301.

[2] D. W. Siderius, V. K. Shen, R. Johnson III, R. D. van Zee, *NIST/ARPA-E Database of Novel and Emerging Adsorbent Materials,  NIST Standard Reference Database Number 205*, Http://Adsorbents.Nist.Gov
